# Efficacy and safety of traditional Chinese classic prescriptions combined with metformin in the treatment of type 2 diabetes mellitus: a Bayesian network meta-analysis

**DOI:** 10.3389/fphar.2026.1693378

**Published:** 2026-02-11

**Authors:** Linlin Che, Wei Dong, Ya Liu, Hongwei Guo

**Affiliations:** Heilongjiang University of Traditional Chinese Medicine, Harbin, China

**Keywords:** metformin, network meta-analysis, systematic review, traditional Chinese classic prescriptions, type 2 diabetes mellitus

## Abstract

**Background:**

Type 2 diabetes mellitus (T2DM) is a metabolic disorder characterized by chronic hyperglycemia. While biomedicine (e.g., metformin) serves as the first-line treatment, combination therapies involving botanical drugs are increasingly utilized. However, the comparative efficacy of different botanical formulations remains to be systematically evaluated.

**Aim:**

This study aims to analyze randomized controlled trials (RCTs) to evaluate the clinical efficacy and safety of eight specific classical botanical drug formulas combined with metformin (Met) for T2DM, providing evidence to support integrated clinical management.

**Methods:**

A Bayesian network meta-analysis (NMA) was performed on RCTs sourced from PubMed, Cochrane Library, Embase, Web of Science, and Chinese databases. The taxonomic validation of all botanical drugs was conducted using MPNS. Outcomes including HbA1c, FPG, and lipid profiles were assessed using the SUCRA and GRADE methodology.

**Results:**

Forty RCTs involving 3,088 patients were included. In terms of glycemic control, the combination of *Huanglian Jiedu* Decoction (HLJDD) and Met ranked highest for reducing FPG [MD = −1.46, 95% CrIs=(−2.24, −0.68)], while HLJDD, *Zhibai Dihuang* Decoction (ZBDHD), and Dachaihu Decoction (DCHD) combined with Met emerged as the most effective for reducing HbA1c. Notably, sensitivity analysis restricted to trials ≥8 weeks identified HLJDD [MD = −1.02, 95% CrIs=(−1.39, −0.66)] and ZBDHD [MD = −1.02, 95% CrIs=(−1.28, −0.76)] as the most robust interventions for long-term glycemic control. Safety reporting was limited, with only 8 out of 40 trials (20.0%) providing data on adverse events. While no severe adverse events were reported in these specific trials, the overall safety evidence remains uncertain due to substantial missingness and potential under-reporting.

**Conclusion:**

The current NMA results suggest that HLJDD combined with Met is a consistently effective option for reducing both FPG and HbA1c. For HbA1c improvement, while DCHD combined with Met showed initial potential, sensitivity analysis restricted to trials ≥8 weeks identified HLJDD and ZBDHD as the most physiologically robust interventions. However, given the “Very Low” certainty of evidence for many comparisons, these rankings remain exploratory.

## Introduction

Type 2 diabetes mellitus (T2DM), a metabolic disorder characterized by insulin resistance (IR) and impaired β-cell function, has garnered widespread attention due to its high prevalence and multifaceted complications (Młynarska et al., 2025). According to the 11th Edition of the International Diabetes Federation (IDF) Diabetes Atlas, approximately 589 million adults aged 20–79 years worldwide were affected by diabetes in 2025, representing 11.1% of the global population in this age group, with projections indicating a rise to 853 million (13%) by 2050 (Fiorentino et al., 2025). In recent years, T2DM has emerged as a severe global public health threat, paralleled by an expanding repertoire of pharmacological interventions for its management (Liu C. et al., 2025).

While antidiabetic pharmacotherapy represents an ideal clinical approach, many agents carry risks of hypoglycemia, fluid retention, cardiovascular events, and other adverse reactions, raising concerns regarding optimal treatment selection for maximal efficacy with minimal adverse effects (Susilawati et al., 2023; Khunti et al., 2025; Tassopoulou et al., 2025). Metformin (Met), a first-line pharmacologic intervention for T2DM, demonstrates well-established glucose-lowering efficacy with significant improvement in T2DM-related clinical manifestations despite incomplete mechanistic elucidation (American Diabetes Association Professional Practice Committee, 2025; Somasundaram et al., 2025). However, its benefits on glucose and lipid metabolism are counterbalanced by gastrointestinal side effects (Mohamed, 2024); moreover, prolonged administration increases risks of lactic acidosis, particularly in renally impaired and elderly populations (De Simone et al., 2025; Liu X. et al., 2025). Consequently, despite Met’s undeniable impact on ameliorating T2DM-induced dysmetabolism, its long-term clinical utility remains constrained.

With the modernization of botanical drug preparations, the combination of classical herbal formulas and biomedicine has gained increasing clinical adoption for T2DM management (Wen et al., 2025). Functioning as a multicomponent pharmacotherapeutic system, botanical formulations utilize a multi-target and synergistic strategy. This approach, characterized by personalized phenotype management, has demonstrated distinct efficacy in addressing the multifactorial nature of T2DM and its complications (Xu et al., 2025). The multicomponent and multi-target properties of botanical formulations provide a strategic rationale for addressing the multifactorial pathophysiology of T2DM (Ni et al., 2024). Widespread clinical implementation has generated robust evidence supporting the efficacy of botanical drug therapies in disease management (Gu et al., 2024; Zhang Q. et al., 2024; Zhang M. et al., 2025). Crucially, combining herbal preparations with Met mitigates adverse effects associated with monotherapy, such as gastrointestinal disturbances (Zheng et al., 2025). Nevertheless, the therapeutic heterogeneity among distinct classical botanical formulas remains unaddressed by comprehensive comparative efficacy analyses, resulting in complexities for evidence-based clinical decision-making.

Although several systematic reviews and meta-analyses have substantiated the efficacy of specific botanical formulas combined with Met, most existing studies have focused on pairwise comparisons of a single intervention against Met monotherapy. This fragmented evidence base leaves a critical knowledge gap: there is a lack of head-to-head comparisons between different classical formulas. Consequently, clinicians face a dilemma when selecting the most appropriate prescription from multiple available options. To address this, a Bayesian network meta-analysis (NMA) is necessary. Unlike traditional meta-analyses, NMA allows for the simultaneous comparison of multiple interventions and generates a hierarchy of efficacy using probability rankings (SUCRA), providing precise, evidence-based guidance for personalized clinical decision-making.

## Methods

This network meta-analysis was reported in strict accordance with the PRISMA extension statement (Elsman et al., 2024). The study protocol was prospectively registered with PROSPERO (CRD420251104100), following the PICOS framework (Population, Intervention, Comparison, Outcomes, and Study design) to define eligibility. To ensure the validity of the network comparisons, we rigorously assessed the assumption of transitivity by examining the distribution of potential effect modifiers across treatment arms. Specifically, we conducted a comprehensive assessment of potential effect modifiers to verify transitivity. Key prognostic baseline characteristics—including mean age, disease duration, gender distribution, BMI, and baseline levels of all efficacy outcome—were systematically compared across the treatment nodes using boxplots to detect any significant imbalances.

### Data sources and search strategies

We systematically searched eight electronic databases, including PubMed, Embase, Cochrane Library, Web of Science, CNKI, Chinese biomedicine, VIP and WANFANG, from their inception up to December 2025. Regarding grey literature, we manually screened the reference lists of included studies and relevant systematic reviews to identify potential eligible trials. While unpublished reports and conference abstracts were not included, academic dissertations and master’s theses from these databases were explicitly included to minimize potential publication bias, as they undergo rigorous institutional review and represent a substantial portion of clinical evidence in China. The specific search strategies for all databases, constructed based on the PICOS principle using a combination of Medical Subject Headings (MeSH) and free-text terms, are detailed in Table 1 and Supplementary Appendix S1. The search strategy specifically targeted randomized controlled trials (RCTs) investigating classical Chinese herbal formulas combined with Met for T2DM management. Included formulas comprised: *Shenling Baizhu* powder (SLBZP), *Zhibai Dihuang* decoction (ZBDHD), *Huanglian Jiedu* decoction (HLJDD), *Huanglian Wendan* decoction (HLWDD), *Gegen Qinlian* decoction (GGQLD), *Dachaihu* decoction (DCHD), *Baihu Renshen* decoction (BHRSD), and *Linggui Zhugan* decoction (LGZGD). All search procedures were independently executed by two investigators.

**TABLE 1:** Search strategy.

Query	Search terms
#1	MeSH descriptor: [Diabetes Mellitus, Type 2] explode all trees
#2	(Diabetes Mellitus, Type 2):ti,ab,kw OR (Type 2 diabetes mellitus):ti,ab,kw OR (Non-insulin-dependent diabetes mellitus):ti,ab,kw OR (Diabetes mellitus, non-insulin-dependent):ti,ab,kw OR (Diabetes mellitus):ti,ab,kw OR (T2DM):ti,ab,kw OR (NIDDM):ti,ab,kw OR (DM):ti,ab,kw
#3	#1 OR #2
#4	MeSH descriptor: [Medicine, Chinese Traditional] explode all trees
#5	(Medicine, Chinese Traditional):ti,ab,kw OR (TCM):ti,ab,kw OR (traditional Chinese medicine):ti,ab,kw OR (Chinese medicinal herb):ti,ab,kw OR (Chinese herbal medicine):ti,ab,kw
#6	(Medicine, Chinese Traditional):ti,ab,kw OR (decoction):ti,ab,kw OR (formula):ti,ab,kw OR (prescription):ti,ab,kw OR (Chinese patent medicine):ti,ab,kw
#7	(Medicine, Chinese Traditional):ti,ab,kw OR (Chinese patent drug):ti,ab,kw OR (Chinese herbal compound prescription):ti,ab,kw
#8	#4 OR #5 OR #6 OR #7
#9	MeSH descriptor: [Clinical Trial] explode all trees
#10	(Clinical Trials):ti,ab,kw OR (Clinical Trials, Randomized):ti,ab,kw OR (Trials, Randomized Clinical):ti,ab,kw OR (Controlled Clinical Trials, Randomized):ti,ab,kw OR (Intervention Study):ti,ab,kw
#11	#9 OR #10
#12	#3 AND #8 AND #11

Abbreviations: ti, title; ab, abstract; kw, keywords.

English search terms included: “Diabetes Mellitus, Type 2”, “Type 2 diabetes mellitus”, “Non-insulin-dependent diabetes mellitus”, “Diabetes mellitus, non-insulin-dependent”, “Diabetes mellitus”, “T2DM”, “NIDDM”, “DM”, “Medicine, Chinese Traditional”, “TCM”, “traditional Chinese medicine”, “Chinese medicinal herb”, “Chinese herbal medicine”, “decoction”, “formula”, “prescription”, “Chinese patent medicine”, “Chinese patent drug”, “Chinese herbal compound prescription”, “Clinical Trial”, “Clinical Trials, Randomized”, “Trials, Randomized Clinical”, “Controlled Clinical Trials, Randomized”, “Intervention Study”.

Chinese search terms included: “2型糖尿病”, “糖尿病”, “二甲双胍”, “中药”, “中医药”, “中药方剂”, “方剂”, “中药汤剂”, “汤剂”, “中药复方”, “临床研究”, “临床观察”, “临床试验”, “干预实验”.

### Eligibility criteria

Studies meeting the following PICOS criteria were eligible for inclusion (Amir-Behghadami and Janati, 2020):

1. Participants: Adults aged ≥18 years diagnosed with T2DM, with or without complications/comorbidities, irrespective of demographic characteristics (e.g., age, ethnicity, sex).
2. Interventions: Clinical trials investigating combination therapy of specified classical Chinese herbal formulas (SLBZP, ZBDHD, HLJDD, HLWDD, GGQLD, DCHD, BHRSD, LGZGD) with Met for T2DM management, including both canonical formulations and modified variants.
3. Comparators: Met monotherapy.
4. Outcomes: Primary outcomes (Confirmatory): Fasting plasma glucose (FPG) and glycated hemoglobin (HbA1c) were pre-specified as the primary confirmatory outcomes to test the study’s central hypothesis, serving as the principal basis for evaluating the efficacy of herbal formulas; Secondary outcomes (Exploratory): Total cholesterol (TC), triglycerides (TG), low-density lipoprotein cholesterol (LDL-c), high-density lipoprotein cholesterol (HDL-c), 2-h postprandial glucose (2hPG), fasting insulin (FINS), and homeostatic model assessment of insulin resistance (HOMA-IR) were categorized as secondary exploratory outcomes. These parameters provided supplementary evidence and were not used as the sole determinants for the final clinical conclusions.
5. Study design: RCTs suitable for systematic review and meta-analysis.

### Exclusion criteria

1. Secondary analyses or duplicate publications (for multilingual publications, only the earliest version was retained).
2. Full-text articles that could not be retrieved after contacting corresponding authors.
3. Studies with inappropriate randomization methods.
4. Missing baseline characteristics data.
5. Absence of clinically validated diagnostic criteria.
6. Incompatibility with prespecified outcome measures.
7. Interventions deviating from the protocol-specified combination of classical Chinese herbal formulas and Met.

### Trial selection

Following duplicate removal using EndNote 20, two investigators independently screened all titles and abstracts against predefined inclusion and exclusion criteria, excluding non-eligible studies; full-text review of potentially eligible studies was conducted to determine final inclusion, with disagreements resolved through discussion or by consulting an arbitrator when consensus was not achieved, and the investigators involved in the screening process held professional qualifications as a physician and a pharmacist respectively.

### Taxonomic validation and nomenclature

The botanical nomenclature of all herbal ingredients included in the analyzed formulas has been validated taxonomically using the Medicinal Plant Names Services (MPNS) (http://mpns.kew.org) and Plants of the World Online (POWO) (http://www.plantsoftheworldonline.org). All plant names are presented with their full scientific names, authorities, families, and pharmacopeial drug names. The complete taxonomic details for all eight formulas are listed in Table 2. Since some studies utilized modified versions of these formulas based on syndrome differentiation, the specific herbal modifications (additions or subtractions) reported in each included study are provided in Supplementary Appendix S2.

**TABLE 2:** Incorporate traditional Chinese medicine components.

Formula name	Scientific name	Family	Latin drug name	Part used
*Shenling Baizhu* powder	*Panax ginseng* C.A.Mey.	Araliaceae	Ginseng Radix et Rhizoma	Root and Rhizome
​	*Atractylodes macrocephala* Koidz.	Asteraceae	Atractylodis Macrocephalae Rhizoma	Rhizome
​	*Wolfiporia cocos* (Schwein.) Ryvarden & Gilb.	Polyporaceae	Poria	Sclerotium
​	*Glycyrrhiza uralensis* Fisch. ex DC.	Fabaceae	Glycyrrhiza Radix et Rhizoma	Root and Rhizome
​	*Dioscorea polystachya* Turcz.	Dioscoreaceae	Dioscoreae Rhizoma	Rhizome
​	*Lablab purpureus* (L.) Sweet	Fabaceae	Lablab Semen Album	Mature Seed
​	*Nelumbo nucifera* Gaertn.	Nelumbonaceae	Nelumbinis Semen	Mature Seed
​	*Coix lacryma-jobi* var. *ma-yuen* (Rom.Caill.) Stapf	Poaceae	Coicis Semen	Dried Ripe Kernel
​	*Wurfbainia villosa* (Lour.) Skornick. and A.D.Poulsen	Zingiberaceae	Amomi Fructus	Dried Ripe Fruit
​	*Platycodon grandiflorus* (Jacq.) A.DC.	Campanulaceae	Platycodonis Radix	Root
*Zhibai Dihuang* decoction	*Rehmannia glutinosa* (Gaertn.) Steud.	Orobanchaceae	Processed Root Tuber	Processed Root Tuber
​	*Cornus officinalis* Siebold & Zucc.	Cornaceae	Sarcocarp (Fruit flesh)	Sarcocarp (Fruit flesh)
​	*Dioscorea polystachya* Turcz.	Dioscoreaceae	Rhizome	Rhizome
​	*Alisma plantago-aquatica* subsp. *orientale* (Sam.) Sam.	Alismataceae	Tuber/Rhizome	Tuber/Rhizome
​	*Paeonia* × *suffruticosa* Andrews	Paeoniaceae	Root Bark	Root Bark
​	*Wolfiporia cocos* (Schwein.) Ryvarden & Gilb.	Polyporaceae	Sclerotium	Sclerotium
​	*Anemarrhena asphodeloides* Bunge	Asparagaceae	Rhizome	Rhizome
​	*Phellodendron chinense* C.K.Schneid.	Rutaceae	Bark	Bark
*Huanglian Jiedu* decoction	*Coptis chinensis* Franch.	Ranunculaceae	Rhizome	Rhizome
​	*Scutellaria baicalensis* Georgi	Lamiaceae	Root	Root
​	*Phellodendron chinense* C.K.Schneid.	Rutaceae	Bark	Bark
​	*Gardenia jasminoides* J.Ellis	Rubiaceae	Fruit	Fruit
*Huanglian Wendan* decoction	*Coptis chinensis* Franch.	Ranunculaceae	Rhizome	Rhizome
​	*Pinellia ternata* (Thunb.) Makino	Araceae	Tuber (Rhizome)	Tuber (Rhizome)
​	*Citrus reticulata* Blanco	Rutaceae	Pericarp (Peel)	Pericarp (Peel)
​	*Phyllostachys nigra* var. *henonis* (Mitford) Stapf ex Rendle	Poaceae	Shavings of caulis	Shavings of caulis
​	*Citrus* × *aurantium* L.	Rutaceae	Immature Fruit	Immature Fruit
​	*Wolfiporia cocos* (Schwein.) Ryvarden & Gilb.	Polyporaceae	Sclerotium	Sclerotium
​	*Glycyrrhiza uralensis* Fisch. ex DC.	Fabaceae	Root and Rhizome	Root and Rhizome
​	*Zingiber officinale* Roscoe	Zingiberaceae	Rhizome (Fresh)	Rhizome (Fresh)
​	*Ziziphus jujuba* Mill.	Rhamnaceae	Fruit	Fruit
*Gegen Qinlian* decoction	*Pueraria montana* var. *lobata* (Willd.) Maesen and S.M.Almeida ex Sanjappa & Predeep	Fabaceae	Root	Root
​	*Scutellaria baicalensis* Georgi	Lamiaceae	Root	Root
​	*Coptis chinensis* Franch.	Ranunculaceae	Rhizome	Rhizome
​	*Glycyrrhiza uralensis* Fisch. ex DC.	Fabaceae	Root and Rhizome	Root and Rhizome
*Dachaihu* decoction	*Bupleurum chinense* DC.	Apiaceae	Root	Root
​	*Scutellaria baicalensis* Georgi	Lamiaceae	Root	Root
​	*Rheum palmatum* L.	Polygonaceae	Root and Rhizome	Root and Rhizome
​	*Citrus* × *aurantium* L.	Rutaceae	Immature Fruit	Immature Fruit
​	*Paeonia lactiflora* Pall.	Paeoniaceae	Root	Root
​	*Pinellia ternata* (Thunb.) Makino	Araceae	Tuber (Rhizome)	Tuber (Rhizome)
​	*Zingiber officinale* Roscoe	Zingiberaceae	Rhizome (Fresh)	Rhizome (Fresh)
​	*Ziziphus jujuba* Mill.	Rhamnaceae	Fruit	Fruit
*Baihu Renshen* decoction	*Anemarrhena asphodeloides* Bunge	Asparagaceae	Rhizome	Rhizome
​	*Panax ginseng* C.A.Mey.	Araliaceae	Root and Rhizome	Root and Rhizome
​	*Glycyrrhiza uralensis* Fisch. ex DC.	Fabaceae	Root and Rhizome	Root and Rhizome
​	*Oryza sativa* L.	Poaceae	Seed (Caryopsis)	Seed (Caryopsis)
​	*Gypsum Fibrosum* (Mineral)	(Mineral)	Mineral	Mineral
*Linggui Zhugan* decoction	*Wolfiporia cocos* (Schwein.) Ryvarden & Gilb.	Polyporaceae	Sclerotium	Sclerotium
​	*Cinnamomum cassia* (L.) J.Presl	Lauraceae	Dried Twig/Young Branch	Dried Twig/Young Branch
​	*Atractylodes macrocephala* Koidz.	Asteraceae	Rhizome	Rhizome
​	*Glycyrrhiza uralensis* Fisch. ex DC.	Fabaceae	Root and Rhizome	Root and Rhizome

### Data extraction and quality assessment

Two investigators independently extracted data and assessed the risk of bias for included studies using a predefined Excel form. Data extraction encompassed: first author, publication year, intervention details, participant characteristics (age, sex, disease duration), sample size, randomization methodology, blinding implementation, outcome measures, intervention/follow-up duration, therapeutic protocols, quantitative outcome values, and additional relevant data. Discrepancies were resolved through discussion with a third reviewer. The Risk of Bias 2 (RoB 2) tool was employed to assess the risk of bias across five domains: (1) bias arising from the randomization process; (2) bias due to deviations from intended interventions; (3) bias due to missing outcome data; (4) bias in measurement of the outcome; and (5) bias in selection of the reported result. Quality assessment was independently performed by two researchers, with disagreements resolved *via* consensus discussions or by consulting a third arbitrator. Each domain was signaled as “Low risk”, “Some concerns”, or “High risk” according to the RoB 2 algorithmic guidance. The overall risk of bias for each trial was determined by the highest risk level assigned to any individual domain.

### Data synthesis and analysis

The assumption of transitivity underpins the validity of NMA. To verify this prerequisite, we assessed not only mean age and disease duration but also the distribution of key effect modifiers across the included RCTs. Furthermore, regarding comorbidities and concomitant medications, strict eligibility criteria were applied during study selection. Studies involving patients with severe cardiovascular, renal, or hepatic complications were excluded, ensuring a clinically homogenous population of T2DM patients. A systematic comparison of these baseline characteristics (summarized in Supplementary Appendix S3) confirmed that there were no clinically important imbalances that could threaten the transitivity assumption.

Network geometry was visualized using Stata 17.0, where node sizes were scaled proportionally to the total sample size of each intervention, and edge thicknesses represented the number of studies for each direct comparison.

The Bayesian NMA was performed using the gemtc package in R. We employed non-informative (vague) prior distributions (Normal distribution *N* (0, 10,000) for effects; Uniform distribution *U* (0, 5) for heterogeneity) to minimize the influence of initial assumptions. The simulation was set up with four chains, 50,000 iterations per chain, a thinning interval of 10, and a burn-in of 20,000 iterations. Model convergence was rigorously assessed using trace plots, density plots, and the Brooks-Gelman-Rubin (BGR) diagnostic, with a Potential Scale Reduction Factor (PSRF) <1.05 indicating satisfactory convergence. We compared the Deviance Information Criterion (DIC) between fixed- and random-effects models; the random-effects model yielded a lower DIC and was selected for the final analysis. Consequently, continuous outcomes were analyzed using mean differences (MDs) with 95% Credible Intervals (CrIs).

We formally assessed the assumption of consistency using both global (DIC comparison) and local approaches. Locally, the node-splitting method was used, with significant inconsistency defined as a *P*-value <0.05. Heterogeneity was quantified using the *I* ^2^ statistic (*I* ^2^ ≥ 50% indicating substantial heterogeneity). Treatment rankings were established by SUCRA values. Publication bias was assessed using comparison-adjusted funnel plots. Quantitative tests for asymmetry were not performed because most comparisons involved fewer than 10 studies, in accordance with Cochrane guidelines. Considering the physiological characteristics of HbA1c (reflecting 8–12 weeks of glycemia) and the significant impact of intervention duration identified in our meta-regression (*P* = 0.028), a pre-specified sensitivity analysis was performed for the HbA1c outcome by excluding trials with a duration of less than 8 weeks.

## Results

### Literature search and characteristics

**FIGURE 1:**
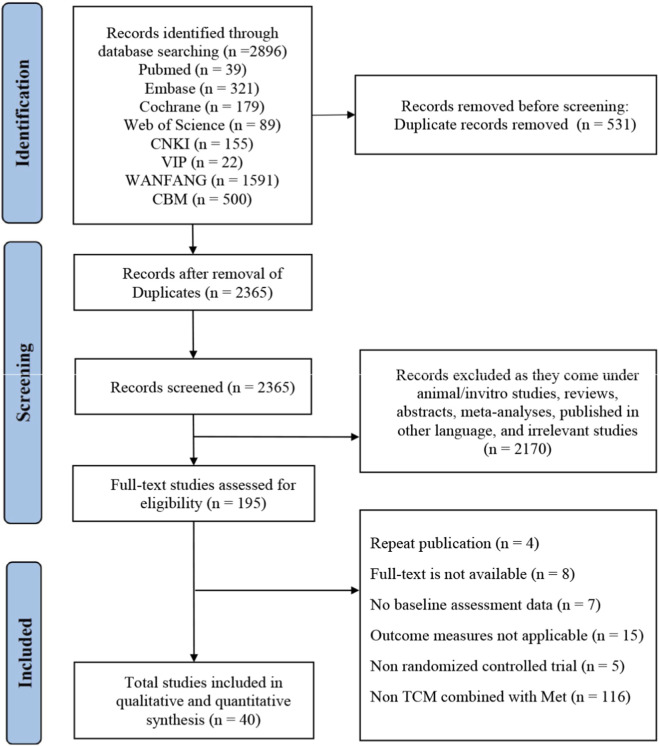
PRISMA flowchart of the selection process.

The initial literature search retrieved 2,896 records. After screening, 195 articles were identified as potentially eligible for full-text review. Following detailed assessment, 155 studies were excluded (see Supplementary Appendix S4 for exclusion reasons). Ultimately, 40 RCTs meeting the inclusion criteria underwent quality assessment and statistical analysis. Chinese references for all included trials are provided in Supplementary Appendix S5. The study selection process is detailed in Figure 1.

All included studies were conducted in China and published in Chinese, enrolling a total of 3,088 patients (mean age range: 34.18–66.14 years). Females accounted for 46.02% (n = 1,421) of the participants. The studies had a mean sample size of 39 (range: 20–100) and covered eight Chinese herbal formulas: SLBZP, ZBDHD, HLJDD, HLWDD, GGQLD, DCHD, BHRSD, and LGZGD. All these formulas were co-administered with Met for treatment. Among the included studies, the combination of GGQLD with Met was the most commonly used therapy (27.50%). Detailed information for each trial is presented in Table 3.

**TABLE 3:** Characteristics of the included studies.

References	Group	Sample size (M/F)	Types of intervention	Age (years)	Duration of intervention	Outcome indicator	Cochrane collaboration risk of bias
Zeng (2022)	T	40 (22/18)	SLBZP + Met	49.38 ± 7.89	8 weeks	①②③④⑤⑥	Unclear; Unclear; Unclear; Unclear; Low; Unclear; Unclear
	C	40 (26/14)	Met	50.68 ± 7.83			
Chen (2015)	T	50 (22/28)	ZBDHD + Met	56.26	2 months	①②③⑨	Low; Low; Low; Unclear; Low; Unclear; Unclear
	C	50 (27/23)	Met	55.90			
Chen and Wang (2021)	T	50 (28/22)	HLJDD + Met	54.37 ± 2.56	12 weeks	①②③④⑤⑥⑦⑧⑨	Low; Unclear; Unclear; Unclear; Low; Unclear; Unclear
	C	49 (25/24)	Met	54.41 ± 2.24			
Chen (2018)	T	30 (12/18)	HLWDD + Met	61.07 ± 9.20	2 months	①②③④⑤⑥⑧	Unclear; Unclear; Unclear; Unclear; Low; Unclear; Unclear
	C	30 (12/18)	Met	58.90 ± 10.27			
Chen (2022)	T	43 (22/21)	HLWDD + Met	57.32 ± 3.23	4 months	①②③⑧	Unclear; Unclear; Unclear; Unclear; Low; Unclear; Unclear
	C	43 (23/20)	Met	57.14 ± 3.64			
Cheng (2018)	T	30 (13/17)	GGQLD + Met	54.17 ± 6.02	4 weeks	①②③④⑤	Low; Unclear; Unclear; Unclear; Low; Unclear; Unclear
	C	30 (17/13)	Met	55.93 ± 5.95			
Cui and Chen (2015)	T	60 (36/24)	DCHD + Met	42.50 ± 11.40	8 weeks	①②③	Unclear; Unclear; Unclear; Unclear; Low; Unclear; Unclear
	C	60 (20/40)	Met	40.30 ± 12.50			
Dai (2022)	T	39 (22/17)	GGQLD + Met	53.16 ± 6.20	8 weeks	①②	Unclear; Unclear; Unclear; Unclear; Low; Unclear; Unclear
	C	39 (23/16)	Met	53.02 ± 7.01			
Feng (2019)	T	45 (25/20)	HLJDD + Met	64.71 ± 7.25	3 months	①③④⑤⑥⑦⑧	Low; Unclear; Unclear; Unclear; Low; Unclear; Unclear
	C	45 (26/19)	Met	64.15 ± 7.52			
Feng et al. (2020)	T	30 (14/16)	BHRSD + Met	59.54 ± 5.39	24 weeks	①②③④⑤⑥⑦	Low; Unclear; Unclear; Unclear; Low; Unclear; Unclear
	C	30 (12/18)	Met	58.85 ± 6.15			
Gong et al. (2012)	T	32 (16/16)	SLBZP + Met	54.90 ± 6.10	12 weeks	①②③④⑤⑧⑨	Unclear; Unclear; Unclear; Unclear; Low; Unclear; Unclear
	C	32 (17/15)	Met	55.30 ± 5.90			
Fu (2017)	T	30 (16/14)	GGQLD + Met	56.07 ± 8.25	12 weeks	①②③④⑤⑥⑦⑧⑨	Low; Unclear; Unclear; Unclear; Low; Unclear; Unclear
	C	30 (17/13)	Met	57.50 ± 8.19			
Gong et al. (2012)	T	31 (16/15)	SLBZP + Met	55.10 ± 6.40	12 weeks	①②③④⑤⑧⑨	Unclear; Unclear; Unclear; Unclear; Low; Unclear; Unclear
	C	34 (18/16)	Met	54.80 ± 7.30			
Pan et al. (2021)	T	41 (20/21)	HLWDD + Met	50.10 ± 5.50	4 months	①⑧⑨	Unclear; Unclear; Unclear; Unclear; Low; Unclear; Unclear
	C	39 (18/21)	Met	51.20 ± 5.40			
Ji (2017)	T	30 (14/16)	HLWDD + Met	56.17	12 weeks	①②③④⑤⑥⑦⑧⑨	Unclear; Unclear; Unclear; Unclear; Low; Unclear; Unclear
	C	30 (12/18)	Met	54.17			
Ji and Che (2020)	T	20 (10/10)	DCHD + Met	55.25 ± 9.93	1 month	①②③	Low; Unclear; Unclear; Unclear; Low; Unclear; Unclear
	C	20 (11/9)	Met	56.50 ± 10.25			
Li (2018)	T	48 (28/20)	GGQLD + Met	58.10 ± 9.70	2 weeks	①②③	Low; Unclear; Unclear; Unclear; Low; Unclear; Unclear
	C	48 (26/22)	Met	59.40 ± 9.80			
Li et al. (2024)	T	30 (23/7)	LGZGD + Met	34.18 ± 4.27	4 weeks	①②④⑤⑥	Unclear; Unclear; Unclear; Unclear; Low; Unclear; Unclear
	C	30 (21/9)	Met	36.33 ± 3.05			
Li and Feng (2023)	T	31 (17/14)	ZBDHD + Met	45.22 ± 5.19	3 months	①②③ ⑧⑨	Low; Unclear; Unclear; Unclear; Low; Unclear; Unclear
	C	31 (18/13)	Met	45.08 ± 5.54			
Liang and Wang (2016)	T	38 (19/19)	LGZGD + Met	56.50 ± 7.80	12 weeks	①②③	Unclear; Unclear; Unclear; Unclear; Low; Unclear; Unclear
	C	38 (20/18)	Met	55.60 ± 8.60			
Li et al. (2023)	T	40 (29/11)	LGZGD + Met	41.23 ± 10.54	3 months	①②③④⑤⑥⑦⑧⑨	Low; Unclear; Unclear; Unclear; Low; Unclear; Unclear
	C	40 (31/9)	Met	43.73 ± 9.62			
Luo and Zhao (2005)	T	52 (31/21)	ZBDHD + Met	53.50	2 months	①②③	Unclear; Unclear; Unclear; Unclear; Low; Unclear; Unclear
	C	50 (31/19)	Met	54.00			
Ma et al. (2022)	T	55 (26/29)	GGQLD + Met	56.35 ± 2.28	12 weeks	①②③④⑥⑨	Unclear; Unclear; Unclear; Unclear; Low; Unclear; Unclear
	C	55 (28/27)	Met	56.25 ± 2.15			
Peng et al. (2015)	T	30 (13/17)	BHRSD + Met	49.80 ± 7.48	3 months	①②③	Unclear; Unclear; Unclear; Unclear; Low; Unclear; Unclear
	C	30 (16/14)	Met	50.50 ± 7.73			
Rong (2019)	T	40 (23/17)	BHRSD + Met	56.40 ± 5.10	2 months	①②③	Low; Unclear; Unclear; Unclear; Low; Unclear; Unclear
	C	40 (21/19)	Met	55.90 ± 4.80			
Tan (2017)	T	30 (13/17)	SLBZP + Met	59.38 ± 7.07	2 weeks	①②	Low; Unclear; Unclear; Unclear; Low; Unclear; Unclear
	C	30 (14/16)	Met	60.61 ± 7.20			
Tan et al. (2021)	T	100 (52/48)	SLBZP + Met	60.38 ± 6.07	2 weeks	①②	Low; Unclear; Unclear; Unclear; Low; Unclear; Unclear
	C	100 (46/54)	Met	56.38 ± 7.07			
Wang (2022a)	T	40 (25/15)	DCHD + Met	52.08 ± 5.15	4 weeks	①②③	Unclear; Unclear; Unclear; Unclear; Low; Unclear; Unclear
	C	40 (23/17)	Met	51.45 ± 5.73			
Wang (2021)	T	50 (30/20)	GGQLD + Met	49.36 ± 4.64	3 months	①②③④⑤⑥⑦⑧⑨	Low; Unclear; Unclear; Unclear; Low; Unclear; Unclear
	C	50 (29/21)	Met	48.97 ± 4.52			
Wang et al. (2021)	T	30 (15/15)	HLWDD + Met	60.07 ± 7.18	2 months	①②③④⑤⑥⑦⑧	Low; Unclear; Unclear; Unclear; Low; Unclear; Unclear
	C	30 (13/17)	Met	58.70 ± 6.97			
Wang (2022b)	T	25 (16/9)	HLWDD + Met	60.00 ± 1.08	4 months	①②③	Unclear; Unclear; Unclear; Unclear; Low; Unclear; Unclear
	C	25 (15/10)	Met	60.00 ± 1.02			
Wu (2021)	T	27 (13/14)	GGQLD + Met	64.74 ± 10.05	8 weeks	①②③⑧⑨	Low; Unclear; Unclear; Unclear; Low; Unclear; Unclear
	C	29 (15/14)	Met	66.14 ± 9.16			
Xie (2023)	T	20 (12/8)	GGQLD + Met	51.90 ± 2.70	2 months	①②③⑨	Unclear; Unclear; Unclear; Unclear; Low; Unclear; Unclear
	C	20 (11/9)	Met	51.80 ± 2.80			
Yang and Wang (2013)	T	33 (22/11)	HLJDD + Met	42.00 ± 16.00	6 months	①②③⑧⑨	Unclear; Unclear; Unclear; Unclear; Low; Unclear; Unclear
	C	33 (20/13)	Met	40.00 ± 15.00			
Yang (2021)	T	33 (16/17)	GGQLD + Met	46.50 ± 8.70	8 weeks	①②④ ⑤⑥⑦	Unclear; Unclear; Unclear; Unclear; Low; Unclear; Unclear
	C	33 (17/16)	Met	45.60 ± 9.50			
Yu (2023)	T	45 (28/17)	GGQLD + Met	53.75 ± 2.56	60 days	①②③	Unclear; Unclear; Unclear; Unclear; Low; Unclear; Unclear
	C	45 (27/18)	Met	52.85 ± 2.91			
Yuan et al. (2023)	T	43 (23/20)	SLBZP + Met	64.17 ± 6.29	6 months	①②③④⑤⑥⑨	Low; Unclear; Unclear; Unclear; Low; Unclear; Unclear
	C	43 (24/19)	Met	64.24 ± 6.30			
Zhang (2017)	T	37 (22/15)	HLJDD + Met	57.40 ± 5.80	4 weeks	①②③	Unclear; Unclear; Unclear; Unclear; Low; Unclear; Unclear
	C	37 (20/17)	Met	58.40 ± 6.40			
Zhang (2025)	T	36 (19/17)	GGQLD + Met	52.33 ± 2.76	3 months	①②③⑧⑨	Unclear; Unclear; Unclear; Unclear; Low; Unclear; Unclear
	C	36 (20/16)	Met	51.41 ± 2.62			
Zhou (2023)	T	30 (16/14)	DCHD + Met	54.23 ± 9.57	8 weeks	①②⑤⑥⑦⑨	Low; Unclear; Unclear; Unclear; Low; Unclear; Unclear
	C	30 (18/12)	Met	53.90 ± 9.81			

Abbreviations: Met, metformin; SLBZP, *shenling baizhu* powder; ZBDHD:*zhibai dihuang* decoction; HLJDD, *huanglian jiedu* decoction; HLWDD, *huanglian wendan* decoction; GGQLD, *gegen qinlian* decoction; DCHD, *dachaihu* decoction; BHRSD, *baihu renshen* decoction; LGZGD, *linggui zhugan* decoction; M, male; F, female; T, treatment; C, control; ①, FPG; ②, 2hPG; ③, HbA1c; ④, TC; ⑤, TG; ⑥, LDL-c; ⑦, HDL-c; ⑧, FINS; ⑨, HOMA-IR.

Valid NMA requires that potential effect modifiers be balanced across comparisons. We evaluated the baseline comparability of 12 key variables. As shown in Supplementary Appendix S3, the boxplots reveal that the median values and interquartile ranges for demographic factors (Age, Duration, BMI) and metabolic baselines (including HbA1c, FPG, and lipid profiles) were comparable across the eight intervention nodes. No significant systematic differences were observed in the starting severity of diabetes or dyslipidemia among the treatment arms. This balance supports the transitivity assumption and justifies the unadjusted network meta-analysis.

### Assessment of research quality

The risk of bias for each included trial was assessed using the Cochrane risk of bias tool. Regarding the randomization process, 18 studies utilized random number tables for allocation, while the others provided insufficient details. In the domain of deviations from intended interventions, the majority of trials were rated as “Some concerns” due to the lack of blinding of participants and personnel. For missing outcome data and selection of the reported result, no significant concerns were identified in most included studies. Supplementary Appendix S6 presents a summary of the risk of bias within the randomized controlled trials.

### Effects of herbal formulas combined with Met on FPG in patients with T2DM

**FIGURE 2:**
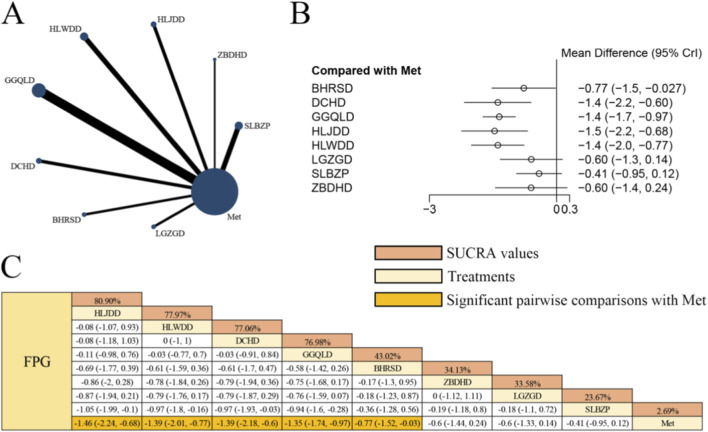
**(A)** Network evidence diagram of FPG **(B)** pairwise meta-analyses: Chinese herbal compound + Met vs. Met **(C)** results of the NMA and SUCRA probability ranking. N*otes:* A negative MD indicates a more significant decrease in FPG levels, representing a clinical advantage of the combined botanical-Met therapy over Met alone.

Figure 2A presents the network evidence graph for FPG from a NMA incorporating 40 randomized controlled trials involving 3,088 participants and evaluating eight integrated botanical and biomedical treatment strategies [SLBZP + Met vs. Met (n = 6), ZBDHD + Met vs. Met (n = 3), HLJDD + Met vs. Met (n = 4), HLWDD + Met vs. Met (n = 6), GGQLD + Met vs. Met (n = 11), DCHD + Met vs. Met (n = 4), BHRSD + Met vs. Met (n = 3), LGZGD + Met vs. Met (n = 3)]; the Met monotherapy group constituted the largest sample size, and comparisons between GGQLD + Met and Met were the most frequently studied; combined herbal compound formula and Met therapy demonstrated significantly superior effects on improving FPG compared to Met alone, specifically HLJDD + Met [MD = −1.46, 95% CrIs=(−2.24, −0.68)], HLWDD + Met [MD = −1.39, 95% CrIs=(−2.01, −0.77)], DCHD + Met [MD = −1.39, 95% CrIs=(−2.18, −0.60)], GGQLD + Met [MD = −1.35, 95% CrIs=(−1.74, −0.97)], and BHRSD + Met [MD = −0.77, 95% CrIs=(−1.52, −0.03)], indicating that HLJDD combined with Met yielded the most significant improvement in FPG levels among patients with T2DM.

### Effects of herbal formulas combined with Met on HbA1c in patients with T2DM

**FIGURE 3:**
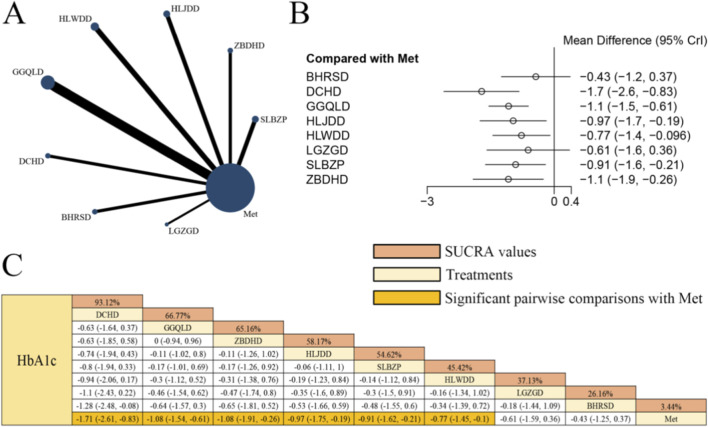
**(A)** Network evidence diagram of HbA1c **(B)** pairwise meta-analyses: Chinese herbal compound + Met vs. Met **(C)** results of the NMA and SUCRA probability ranking. *Notes:* A negative MD indicates a more significant decrease in HbA1c levels, representing a clinical advantage of the combined botanical-Met therapy over Met alone.

Figure 3A displays the network evidence graph for HbA1c from a NMA, incorporating 33 randomized controlled trials involving 2,484 participants and evaluating eight integrated botanical and biomedical treatment strategies [SLBZP + Met vs. Met (n = 4), ZBDHD + Met vs. Met (n = 3), HLJDD + Met vs. Met (n = 4), HLWDD + Met vs. Met (n = 5), GGQLD + Met vs. Met (n = 9), DCHD + Met vs. Met (n = 3), BHRSD + Met vs. Met (n = 3), LGZGD + Met vs. Met (n = 2)]; the Met monotherapy group constituted the largest sample size, and comparisons between GGQLD + Met and Met were the most frequently studied; combined herbal compound formula and Met therapy demonstrated significantly superior efficacy in improving HbA1c compared to Met alone, specifically DCHD + Met [MD = −1.71, 95% CrIs=(−2.61, −0.83)], GGQLD + Met [MD = −1.08, 95% CrIs=(−1.54, −0.61)], ZBDHD + Met [MD = −1.08, 95% CrIs=(−1.91, −0.26)], HLJDD + Met [MD = −0.97, 95% CrIs=(−1.75, −0.19)], SLBZP + Met [MD = −0.91, 95% CrIs=(−1.62, −0.21)], and HLWDD + Met [MD = −0.77, 95% CrIs=(−1.45, −0.10)], suggesting that DCHD combined with Met yielded the most significant improvement in HbA1c levels among patients with T2DM. A sensitivity analysis for HbA1c excluding trials with a duration of <8 weeks was performed to ensure physiological relevance. The findings showed that HLJDD + Met and ZBDHD + Met surpassed DCHD + Met to become the top-ranked interventions (Supplementary Appendix S7). This shift indicates that the efficacy of HLJDD and ZBDHD may be more stable over the 8–12 week period required for HbA1c stabilization, whereas the initial high ranking of DCHD might have been influenced by short-term fluctuations in smaller trials.

### Effects of herbal formulas combined with Met on 2hPG in patients with T2DM

**FIGURE 4:**
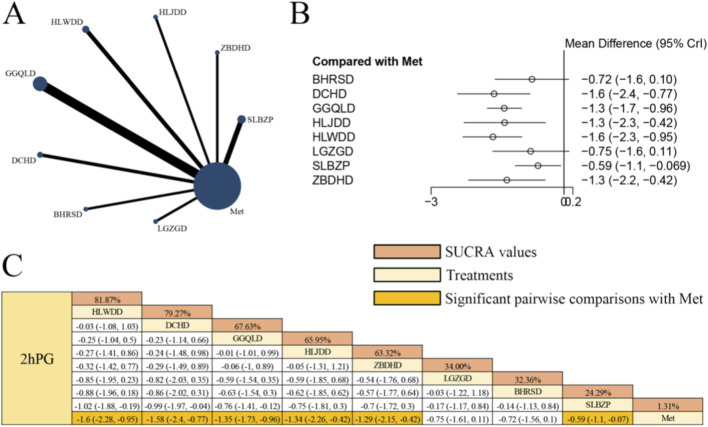
**(A)** Network evidence diagram of 2hPG **(B)** pairwise meta-analyses: Chinese herbal compound + Met vs. Met **(C)** results of the NMA and SUCRA probability ranking. N*otes:* A negative MD indicates a more significant decrease in 2hPG levels, representing a clinical advantage of the combined botanical-Met therapy over Met alone.

Figure 4A displays the network evidence graph for 2hPG from a NMA, incorporating 38 randomized controlled trials involving 2,918 participants and evaluating eight integrated botanical and biomedical treatment strategies [SLBZP + Met vs. Met (n = 6), ZBDHD + Met vs. Met (n = 3), HLJDD + Met vs. Met (n = 3), HLWDD + Met vs. Met (n = 5), GGQLD + Met vs. Met (n = 11), DCHD + Met vs. Met (n = 4), BHRSD + Met vs. Met (n = 3), LGZGD + Met vs. Met (n = 3)]; the Met monotherapy group constituted the largest sample size, and comparisons between GGQLD + Met and Met were the most frequently studied; combined herbal compound formula and Met therapy demonstrated significantly greater efficacy in improving 2hPG compared to Met alone, specifically HLWDD + Met [MD = −1.60, 95% CrIs=(−2.28, −0.95)], DCHD + Met [MD = −1.58, 95% CrIs=(−2.40, −0.77)], GGQLD + Met [MD = −1.35, 95% CrIs=(−1.73, −0.96)], HLJDD + Met [MD = −1.34, 95% CrIs=(−2.26, −0.42)], ZBDHD + Met [MD = −1.29, 95% CrIs=(−2.15, −0.42)], and SLBZP + Met [MD = −0.59, 95% CrIs=(−1.10, −0.07)], suggesting that HLWDD combined with Met yielded the most pronounced improvement in 2hPG levels among patients with T2DM.

### Effects of herbal formulas combined with Met on TC in patients with T2DM

**FIGURE 5:**
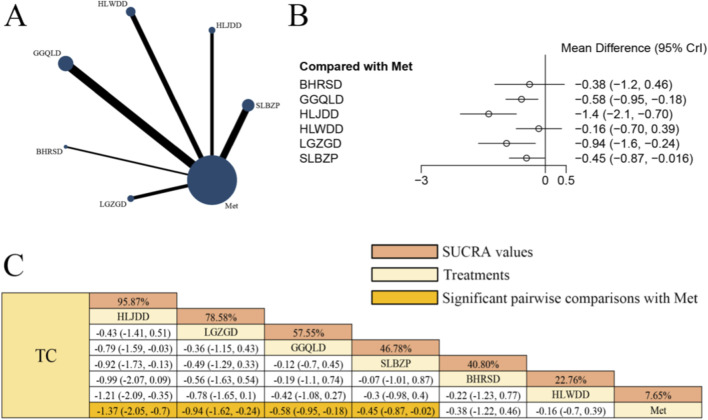
**(A)** Network evidence diagram of TC **(B)** pairwise meta-analyses: Chinese herbal compound + Met vs. Met **(C)** results of the NMA and SUCRA probability ranking. N*otes:* A negative MD indicates a more significant decrease in TC levels, representing a clinical advantage of the combined botanical-Met therapy over Met alone.

Figure 5A presents the network evidence graph for TC from a NMA, incorporating 17 randomized controlled trials involving 1,266 participants and evaluating six integrated botanical and biomedical treatment strategies [SLBZP + Met vs. Met (n = 4), HLJDD + Met vs. Met (n = 2), HLWDD + Met vs. Met (n = 3), GGQLD + Met vs. Met (n = 5), BHRSD + Met vs. Met (n = 1), LGZGD + Met vs. Met (n = 2)]; the Met monotherapy group constituted the largest sample size, and comparisons between GGQLD + Met and Met were the most frequently studied; combined herbal compound formula and Met therapy demonstrated significantly superior effects on improving TC compared to Met alone, specifically HLJDD + Met [MD = −1.37, 95% CrIs=(−2.05, −0.70)], LGZGD + Met [MD = −0.94, 95% CrIs=(−1.62, −0.24)], GGQLD + Met [MD = −0.58, 95% CrIs=(−0.95, −0.18)], and SLBZP + Met [MD = −0.45, 95% CrIs=(−0.87, −0.02)], suggesting that HLJDD combined with Met showed a potential trend towards improvement in TC levels among patients with T2DM. However, these rankings are exploratory and should be interpreted with caution due to the significant heterogeneity observed.

### Effects of herbal formulas combined with Met on TG in patients with T2DM

**FIGURE 6:**
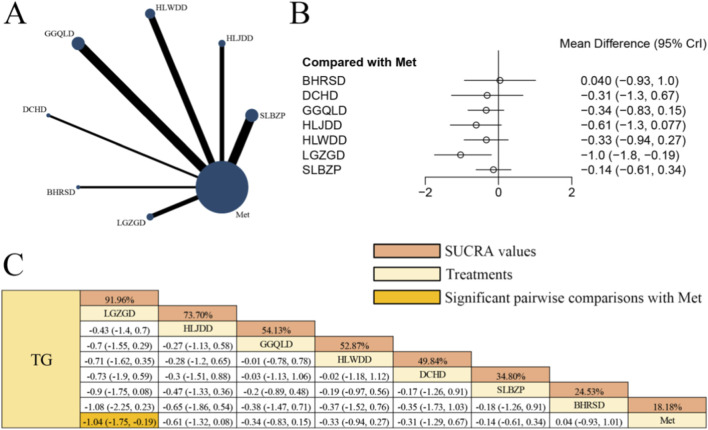
**(A)** Network evidence diagram of TG **(B)** pairwise meta-analyses: Chinese herbal compound + Met vs. Met **(C)** results of the NMA and SUCRA probability ranking. No*tes:* A negative MD indicates a more significant decrease in TG levels, representing a clinical advantage of the combined botanical-Met therapy over Met alone.

Figure 6A presents the network evidence graph for TG from a NMA, incorporating 17 randomized controlled trials involving 1,210 participants and evaluating seven integrated botanical and biomedical treatment strategies [SLBZP + Met vs. Met (n = 4), HLJDD + Met vs. Met (n = 2), HLWDD + Met vs. Met (n = 3), GGQLD + Met vs. Met (n = 4), DCHD + Met vs. Met (n = 1), BHRSD + Met vs. Met (n = 1), LGZGD + Met vs. Met (n = 2)]; the Met monotherapy group constituted the largest sample size, with comparisons between SLBZP + Met and Met as well as GGQLD + Met and Met representing the most frequently studied interventions; notably, LGZGD combined with Met demonstrated a potential benefit in improving TG levels compared to Met monotherapy [MD = −1.04, 95% CrIs=(−1.75, −0.19)] among patients with T2DM. Nevertheless, the high variability in results between primary trials for LGZGD limits the certainty of this finding.

### Effects of herbal formulas combined with Met on LDL-c in patients with T2DM

**FIGURE 7:**
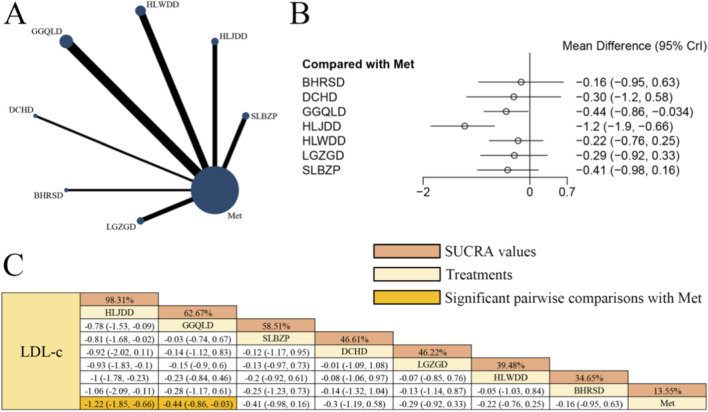
**(A)** Network evidence diagram of LDL-c **(B)** pairwise meta-analyses: Chinese herbal compound + Met vs. Met **(C)** results of the NMA and SUCRA probability ranking. *Notes:* A negative MD indicates a more significant decrease in LDL-c levels, representing a clinical advantage of the combined botanical-Met therapy over Met alone.

Figure 7A displays the network evidence graph for LDL-c from a NMA, incorporating 15 randomized controlled trials involving 1,131 participants and evaluating seven integrated botanical and biomedical treatment strategies [SLBZP + Met vs. Met (n = 2), HLJDD + Met vs. Met (n = 2), HLWDD + Met vs. Met (n = 3), GGQLD + Met vs. Met (n = 4), DCHD + Met vs. Met (n = 1), BHRSD + Met vs. Met (n = 1), LGZGD + Met vs. Met (n = 2)]; the Met monotherapy group constituted the largest sample size, and comparisons between GGQLD + Met and Met were the most frequently studied; combined herbal compound formula and Met therapy demonstrated significantly superior efficacy in improving LDL-c compared to Met alone, specifically HLJDD + Met [MD = −1.22, 95% CrIs=(−1.85, −0.66)] and GGQLD + Met [MD = −0.44, 95% CrIs=(−0.86, −0.03)], suggesting that HLJDD combined with Met yielded the potential benefits in LDL-c levels among patients with T2DM.

### Effects of herbal formulas combined with Met on HDL-c in patients with T2DM

**FIGURE 8:**
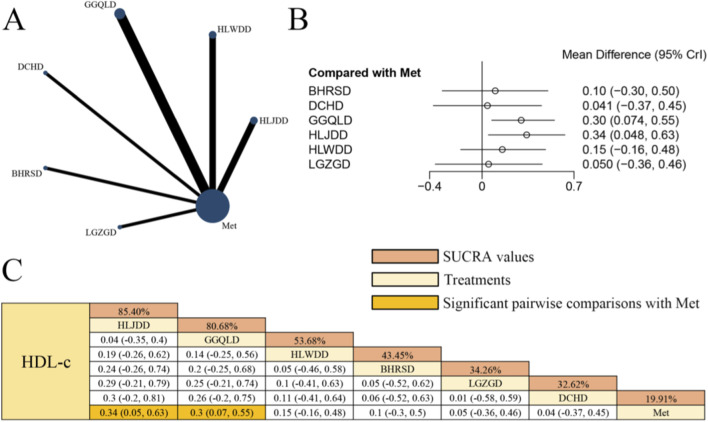
**(A)** Network evidence diagram of HDL-c **(B)** pairwise meta-analyses: Chinese herbal compound + Met vs. Met **(C)** results of the NMA and SUCRA probability ranking. N*otes:* For this specific outcome, a positive MD (increase) indicates a clinical benefit, as higher levels of HDL-c are associated with improved metabolic health.

Figure 8A presents the network evidence graph for HDL-c from a NMA, incorporating 10 randomized controlled trials involving 735 participants and evaluating six integrated botanical and biomedical treatment strategies [HLJDD + Met vs. Met (n = 2), HLWDD + Met vs. Met (n = 2), GGQLD + Met vs. Met (n = 3), DCHD + Met vs. Met (n = 1), BHRSD + Met vs. Met (n = 1), LGZGD + Met vs. Met (n = 1)]; the Met monotherapy group constituted the largest sample size, and comparisons between GGQLD + Met and Met were the most frequently studied; combined herbal compound formula and Met therapy demonstrated a significantly more favorable effect on improving HDL-c levels compared to Met alone, specifically HLJDD + Met [MD = 0.34, 95% CrIs=(0.05, 0.63)] and GGQLD + Met [MD = 0.30, 95% CrIs=(0.07, 0.55)], indicating that HLJDD combined with Met yielded the potential benefits in HDL-c levels among patients with T2DM.

### Effects of herbal formulas combined with Met on FINS in patients with T2DM

**FIGURE 9:**
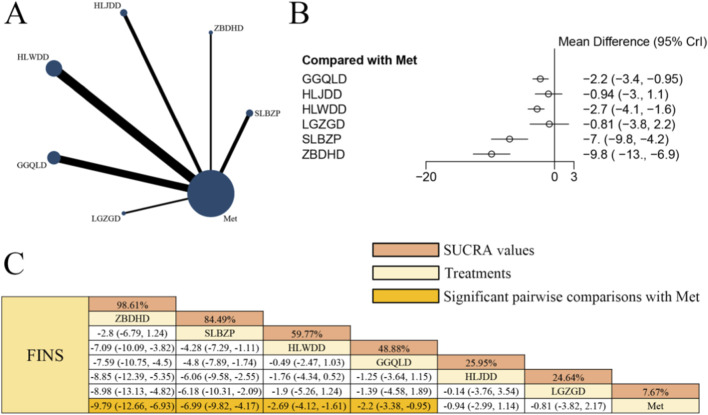
**(A)** Network evidence diagram of FINS **(B)** pairwise meta-analyses: Chinese herbal compound + Met vs. Met **(C)** results of the NMA and SUCRA probability ranking. *Notes:* A negative MD indicates a more significant decrease in FINS levels, representing a clinical advantage of the combined botanical-Met therapy over Met alone.

Figure 9A presents the network evidence graph for FINS from a NMA, incorporating 15 randomized controlled trials involving 1,094 participants and evaluating six integrated botanical and biomedical treatment strategies [SLBZP + Met vs. Met (n = 2), ZBDHD + Met vs. Met (n = 1), HLJDD + Met vs. Met (n = 2), HLWDD + Met vs. Met (n = 5), GGQLD + Met vs. Met (n = 4), LGZGD + Met vs. Met (n = 1)]; the Met monotherapy group constituted the largest sample size, and comparisons between HLWDD + Met and Met were the most frequently studied; combined herbal compound formula and Met therapy demonstrated significantly superior efficacy in improving FINS levels compared to Met alone, specifically ZBDHD + Met [MD = −9.79, 95% CrIs=(−12.66, −6.93)], SLBZP + Met [MD = −6.99, 95% CrIs=(−9.82, −4.17)], HLWDD + Met [MD = −2.69, 95% CrIs=(−4.12, −1.61)], and GGQLD + Met [MD = −2.20, 95% CrIs=(−3.38, −0.95)], suggesting that ZBDHD combined with Met yielded the potential benefits in FINS levels among patients with T2DM.

### Effects of herbal formulas combined with Met on HOMA-IR in patients with T2DM

**FIGURE 10:**
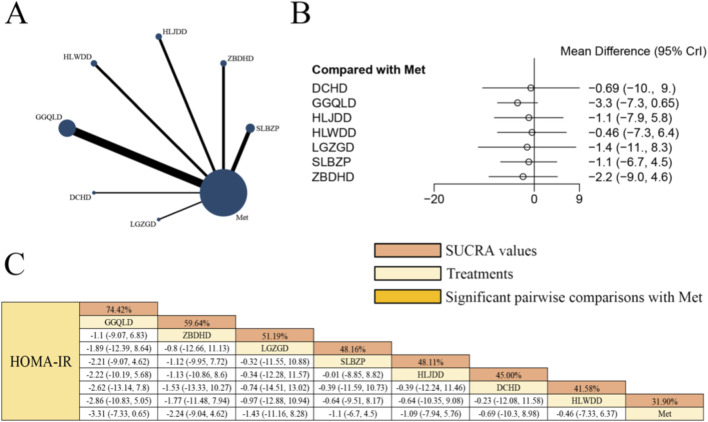
**(A)** Network evidence diagram of HOMA-IR **(B)** pairwise meta-analyses: Chinese herbal compound + Met vs. Met **(C)** results of the NMA and SUCRA probability ranking. *Notes:* A negative MD indicates a more significant decrease in HOMA-IR levels, representing a clinical advantage of the combined botanical-Met therapy over Met alone.

Figure 10A presents the network evidence graph for HOMA-IR from a NMA, incorporating 17 randomized controlled trials involving 1,260 participants and evaluating seven integrated botanical and biomedical treatment strategies [SLBZP + Met vs. Met (n = 3), ZBDHD + Met vs. Met (n = 2), HLJDD + Met vs. Met (n = 2), HLWDD + Met vs. Met (n = 2), GGQLD + Met vs. Met (n = 6), DCHD + Met vs. Met (n = 1), LGZGD + Met vs. Met (n = 1)]; the Met monotherapy group constituted the largest sample size, and comparisons between GGQLD + Met and Met were the most frequently studied; integrated therapy showed no statistically significant differences in improving HOMA-IR compared to Met alone (all mean differences for herbal combinations failed to reach statistical significance with 95% confidence intervals crossing zero), therefore the current evidence does not support superior efficacy of combination regimens over Met monotherapy for improving HOMA-IR in patients with T2DM.

### Publication bias

Supplementary Appendix S8 displays the results of the comparison-adjusted funnel plots for each outcome, where different inter ventions are identified by distinct colored points; the results reveal that most points are evenly distributed on both sides of the midline with substantial left-right symmetry, indicating low publication bias among the included literature; however, a few points lie beyond the two dotted lines, suggesting the presence of heterogeneity between studies and implying the existence of certain publication bias, the causes of which may be related to small sample sizes, incomplete clinical data, or a lack of blinding methods in data collection and processing.

### Inconsistency assessment

The assumption of consistency was evaluated using both local and global approaches. First, local inconsistency was assessed using the node-splitting method. The results showed no significant differences between direct and indirect evidence for any comparison in the glycemic outcomes (all P > 0.05). Furthermore, the global inconsistency of the network was evaluated using the design-by-treatment interaction model. The results indicated that the consistency assumption was upheld for most outcomes, including HbA1c (X^2^ = 1.78, *P* = 0.1823), FPG (X^2^ = 2.78, *P* = 0.0957), 2hPG (X^2^ = 3.18, *P* = 0.0746), LDL-c (X^2^ = 1.25, *P* = 0.2634), HDL-c (X^2^ = 0.15, *P* = 0.7011), HOMA-IR (X^2^ = 0.12, *P* = 0.7277) and FINS (X^2^ = 0.40, *P* = 0.5249). Significant global inconsistency was detected for TC (X^2^ = 9.38, *P* = 0.0022) and TG (X^2^ = 9.46, *P* = 0.0021). To investigate this, we performed a node-splitting analysis (Supplementary Appendix S9). To assess the influence of direct and indirect evidence on the network estimates, a network contribution matrix was generated for each outcome (Supplementary Appendix S10). The matrix illustrates that, due to the star-shaped network geometry, the comparisons between different herbal formulas are entirely derived from indirect evidence *via* the common comparator (Met). While the star-shaped network architecture limits the assessment of loop inconsistency, the analysis identified substantial within-node heterogeneity as the primary source of conflict. For instance, in the TC network, studies evaluating SLBZP showed highly divergent effect sizes (MD ranging from −0.07 to −0.87). Similarly, for TG, the LGZGD + Met comparison exhibited contradictory results across trials (MD ranging from −1.75 to 0.11). These discrepancies are likely attributable to variations in baseline lipid severity and patient baseline characteristics across the primary studies. Consequently, the hierarchy of efficacy for lipid outcomes should be regarded as preliminary and interpreted with significant caution.

### Meta-regression analysis

To investigate the potential sources of heterogeneity, multivariate meta-regression analyses were performed across all primary and secondary outcomes. The covariates included risk of bias, intervention duration, sample size, and prescription modification.

The analysis revealed that intervention duration was a robust and consistent predictor of efficacy for glycemic and insulin parameters. Specifically, longer treatment duration was significantly associated with greater reductions in HbA1c (*P* = 0.028), FPG (*P* = 0.022), and FINS (*P* = 0.013), with a similar borderline trend observed for 2hPG (*P* = 0.053). This suggests that the clinical benefits of the combined therapy are time-dependent, favoring longer treatment courses.

Regarding insulin resistance, sample size was identified as a significant source of heterogeneity for HOMA-IR (P < 0.001), indicating the presence of small-study effects where smaller trials tended to report larger effect sizes. In contrast, no significant covariates were identified for lipid profiles (TC, LDL-c, HDL-c; all *P* > 0.05), although TG showed a borderline association with intervention duration (*P* = 0.060). Other covariates, including risk of bias and herbal modification, did not significantly contribute to the heterogeneity in any outcome. Detailed results are presented in Supplementary Appendix S11.

### Certainty of evidence

The certainty of evidence for each outcome was assessed using the GRADE framework and is summarized in Supplementary Appendix S12. While HLJDD and DCHD ranked highest in SUCRA scores for FPG and HbA1c respectively, the certainty of evidence for these comparisons was rated as Very Low. This downgrading was primarily due to risk of bias in primary studies and imprecision of the estimates. Therefore, the superior ranking of these formulas should be interpreted as indicating potential efficacy rather than definitive clinical superiority.

## Discussion

Currently, the management of type 2 diabetes mellitus (T2DM) relies on pharmacotherapy and physical exercise, as there is no known cure for the condition (Knowler et al., 2025). Metformin (Met) has emerged as the most widely prescribed oral antidiabetic agent in the clinical treatment of T2DM due to its high cost-effectiveness, despite adverse effects such as diarrhea, nausea, abdominal discomfort, bloating, headache, and fatigue (Yao et al., 2025). With advancements in botanical drug research, the clinical application of these therapies in managing glucose and lipid metabolism disorders has gained increasing recognition (Zhang Y. et al., 2024). Chinese herbal medicine and other natural products possess potent pharmacological activities, demonstrating diverse effects including anti-inflammatory, antioxidant, and anti-diabetic actions (Li S. et al., 2025; Liu J. et al., 2025; Zhang J. et al., 2025). Beyond exhibiting therapeutic efficacy in ameliorating the disease, botanical interventions, particularly when combined with conventional pharmacotherapy, yield superior outcomes compared to pharmacotherapy alone (Chen et al., 2025; Li J. et al., 2025). This synergistic effect facilitates the dosage reduction of conventional antidiabetic agents and mitigates their potential toxicities and side effects. Furthermore, the distinct benefit of botanical formulations lies in their compositional flexibility, rendering them particularly suitable for T2DM patients requiring long-term medication for disease control.

This study represents the first network meta-analysis (NMA) to evaluate the efficacy of combining classical traditional Chinese medicine formulas with Met for treating T2DM in China. Although evidence suggests that Chinese medicine formulas, either alone or in combination with Met, can effectively alleviate T2DM symptoms, it is important to note that combining multiple medications with relatively limited efficacy may impact patient outcomes and lead to inefficient allocation of healthcare resources. Therefore, we aimed to identify the optimal combination of botanical agents and biomedicine for T2DM management. The initial screening encompassed a broader range of candidate herbal formulations; however, some lacked standardized compositions and had not undergone rigorous scientific investigation, as they primarily relied on the empirical experience of traditional practitioners. Consequently, following a comprehensive literature review, this study ultimately included classical botanical formulas with a well-documented history of clinical application.

The composition of all included classical prescriptions was defined based on the Pharmacopoeia of the People’s Republic of China (2020 Edition). While the specific botanical origin and medicinal parts (e.g., rhizome for Coptis chinensis) are standardized in the pharmacopeia, it must be noted that detailed phytochemical profiling (e.g., HPLC fingerprints) was not consistently reported in the original RCTs reviewed. Most included studies utilized standard decoctions or NMPA-approved granules prepared according to pharmacopeial monographs, but lacked independent chemical verification. This is a recognized limitation in the reporting quality of primary botanical drug clinical trials.

*Shenling Baizhu* powder (SLBZP), originating from the *Taiping Huimin Hejiju Fang* compiled during the Song Dynasty, is traditionally indicated for tonifying Qi and fortifying the spleen (Gao et al., 2024). Modern pharmacological studies have confirmed that this formula possesses the effect of regulating glucose and lipid metabolism. A randomized, double-blind, placebo-controlled trial demonstrated that SLBZP-based intervention improves blood glucose and lipid levels as well as β-cell function in patients with T2DM and obesity (Huang et al., 2019; Huang et al., 2022). Additionally, SLBZP improves gastrointestinal function and is commonly used to treat chronic gastritis and diarrhea (Jin et al., 2022); when co-administered with Met, it can ameliorate the gastrointestinal adverse effects associated with Met. *Zhibai Dihuang* decoction (ZBDHD), developed from the foundational *Liuwei Dihuang* decoction, has been shown in clinical studies to exert hypoglycemic effects while also enhancing immune function and demonstrating antioxidant activity (Wu et al., 2020). *Huanglian Jiedu* decoction (HLJDD), a renowned classical Chinese medicine formula with millennia of clinical application, exhibits beneficial effects in treating diabetes mellitus and its complications (Zhuang et al., 2025); additionally, it possesses actions for improving lipid profiles and conferring anti-obesity effects (Zheng et al., 2024). *Huanglian Wendan* decoction (HLWDD), originating from the Qing Dynasty text *Liu Yin Tiao Bian*, is clinically employed for treating T2DM and demonstrates effects in ameliorating dyslipidemia and mitigating insulin resistance (IR) (Shi et al., 2022; Liu Z. C. et al., 2025). Meta-analysis results indicate that HLWDD can improve blood glucose and lipid levels in T2DM patients, suggesting its potential as a therapeutic agent for T2DM (Tian et al., 2023). *Gegen Qinlian* decoction (GGQLD), originating from the Eastern Han Dynasty, has long been utilized in the treatment of common metabolic disorders such as T2DM (Xu et al., 2020; Lu et al., 2021). Meta-analysis results indicate that GGQLD exhibits certain therapeutic efficacy and safety in improving glucose and lipid metabolism and alleviating IR, suggesting its potential as a complementary therapy for T2DM (Tan et al., 2023). *Dachaihu* decoction (DCHD), originating from the Eastern Han Dynasty text *Shang Han Lun* authored by Zhongjing Zhang, has been shown by meta-analysis to effectively modulate glucose and lipid metabolism, reduce IR, and improve pancreatic islet function (Zhang et al., 2022; Mou et al., 2024). *Baihu Renshen* decoction (BHRSD), recorded in *Shang Han Lun* for its therapeutic effects on “*Xiao Ke*”, is established as a classical formula of historical significance (Tian et al., 2020). Yao et al., based on animal experiments, revealed that BHRSD ameliorates T2DM by improving hepatic and renal function, alleviating hyperglycemia, hyperlipidemia, pathological changes, oxidative stress, and inflammatory responses. Additionally, the mechanism of BHRSD against T2DM is potentially mediated through the repair of the intestinal barrier, inhibition of TLR4/NF-κB-mediated inflammatory signaling, and amelioration of gut microbiota dysbiosis (Yao et al., 2022). *Linggui Zhugan* decoction (LGZGD), derived from the classical text *Jin Gui Yao Lue*, is demonstrated in contemporary clinical botanical medicine practice to effectively combat obesity and alleviate IR, effects that may be closely correlated with its influence on gut microbiota (Wu et al., 2019).

We assessed the quality of 40 studies investigating the effects of classical Chinese medicine formulas combined with Met in treating T2DM, among which allocation concealment posed the highest risk of introducing selection bias.

This study enrolled a total of 3,088 patients. Levels of fasting plasma glucose (FPG), 2-h postprandial glucose (2hPG), glycated hemoglobin (HbA1c), total cholesterol (TC), triglycerides (TG), low-density lipoprotein cholesterol (LDL-c), high-density lipoprotein cholesterol (HDL-c), fasting insulin (FINS), and homeostasis model assessment of insulin resistance (HOMA-IR) were assessed for eight Chinese medicine formula plus metformin combination therapies: SLBZP + Met, ZBDHD + Met, HLJDD + Met, HLWDD + Met, GGQLD + Met, DCHD + Met, BHRSD + Met, and LGZGD + Met. According to the results of this NMA, the most frequently employed combination intervention among the included studies was *Gegen Qinlian* decoction plus Met. Compared to Met monotherapy, the *Huanglian Wendan* decoction plus Met combination demonstrated superior efficacy in reducing patient FPG and improving lipid profiles, specifically TC, LDL-c, and HDL-c levels. The *Huanglian Wendan* decoction plus Met combination showed significantly greater therapeutic effects than Met alone in lowering patient 2hPG. The *Dachaihu* decoction plus Met combination was more effective than Met monotherapy in reducing patient HbA1c. Interestingly, after excluding studies with a duration of <8 weeks in the sensitivity analysis, the ranking of HLJDD + Met and ZBDHD + Met surpassed that of DCHD + Met. This shift is physiologically plausible, as HbA1c reflects the average blood glucose level over the preceding 2–3 months. The high SUCRA ranking of DCHD in the primary analysis might have been influenced by short-term fluctuations in smaller trials, whereas the sustained efficacy of HLJDD and ZBDHD over longer periods (≥8 weeks) provides more robust evidence for chronic glycemic management. The *Linggui Zhugan* decoction plus Met combination exhibited greater efficacy than Met alone in lowering patient TG levels. Furthermore, the *Zhibai Dihuang* decoction plus Met combination demonstrated significantly greater reductions in patient FINS compared to Met monotherapy. Comprehensive analysis identified the *Huanglian Jiedu* decoction plus Met combination as showing promising efficacy for glycemic control in T2DM. While it also appeared favorable for multiple aspects of lipid metabolism, the substantial inconsistency in the lipid evidence base necessitates a more conservative interpretation of its overall metabolic benefits. It is noteworthy that while lipid profiles and FPG improved, the pooled effect on HOMA-IR did not reach statistical significance. Several factors may contribute to this negative finding. First, the treatment duration in most included RCTs was relatively short (8–12 weeks), which may be insufficient to reverse chronic IR. Although some included RCTs had relatively short treatment durations (2–4 weeks), which may be insufficient to fully reflect changes in HbA1c, these trials were initially included to maintain network connectivity and maximize the use of available clinical data. However, the sensitivity analysis restricted to trials ≥8 weeks ensures that our conclusions are physiologically sound. Second, HOMA-IR is a surrogate marker calculated from FPG and FINS; variability in insulin assays across different hospitals can introduce substantial heterogeneity, masking potential benefits. Third, statistical power was limited as fewer studies reported HOMA-IR compared to FPG.

According to the summary report, observational findings indicate that *Glycyrrhiza uralensis* Fisch. ex DC. (Gan Cao; 62.5%), *Wolfiporia cocos* (Schwein.) Ryvarden & Gilb. (Fu Ling; 52.5%), and *Coptis chinensis* Franch. (Huang Lian; 52.5%) are the most frequently utilized medicinal herbs across various traditional Chinese medicine formulas. Recent research demonstrates that glycyrrhizic acid, the primary bioactive constituent of licorice, possesses immunomodulatory, anti-inflammatory, antioxidant, and anti-diabetic properties, particularly against T2DM. These effects include reducing blood glucose levels, lowering serum insulin levels, enhancing insulin sensitivity, improving glucose tolerance and homeostasis, and modulating lipid metabolism. Furthermore, glycyrrhizic acid exhibits beneficial effects against diabetic complications (Tan et al., 2022). Isoliquiritigenin, a chalcone compound isolated from licorice, significantly reduced blood glucose levels and attenuated IR in diabetic mice without apparent side effects, revealing the substantial potential of licorice as a novel candidate therapeutic agent for the prevention and treatment of T2DM (Yang et al., 2022). Among the 30 anti-diabetic formulas approved by the China National Medical Products Administration, Poria cocos is one of the most commonly used herbs. A meta-analysis revealed that herbal formulas containing Poria cocos, when combined with hypoglycemic agents, further reduced glycemic indices—including FPG, 2hPG, and HbA1c—as well as lipid metabolism parameters in overweight T2DM patients (Di et al., 2022). Polysaccharides derived from Poria cocos significantly decreased fasting blood glucose and markedly reduced HbA1c levels in T2DM animal models; glucose tolerance and IR were also improved, alongside amelioration of lipid metabolism in the experimental animals (Sun et al., 2019). Coptis polysaccharides exert anti-diabetic activity in T2DM rats *via* their antioxidant actions (Jiang et al., 2015). In a recent animal experiment, oral administration of Coptis polysaccharides ameliorated hyperglycemia, IR, dyslipidemia, and β-cell function in T2DM mice. Additionally, a meta-analysis demonstrated that herbal formulas containing Coptis exhibit favorable therapeutic effects for T2DM (Pan et al., 2022).

We analyzed eight RCTs reporting adverse events and categorized the adverse reactions observed in patients receiving either Chinese medicine monotherapy or combination therapy into three types: mild gastrointestinal discomfort, diarrhea, and nausea/vomiting. For these adverse reactions, adjusting the dosing regimen to postprandial administration alleviated the symptoms. Dosing time is a critical consideration in Chinese medicine therapy. While most decoctions should be administered preprandially, postprandial dosing is recommended when the formula contains components known to irritate the gastrointestinal tract or when the patient exhibits compromised gastrointestinal function. Literature synthesis revealed that Chinese medicine formulas causing gastrointestinal adverse reactions consistently contained cold-natured herbs such as Scutellaria baicalensis Georgi (*Huang Qin*) and Coptis chinensis Franch. (*Huang Lian*). This aligns with the classical botanical theory that “bitter-cold herbs damage gastric function” (*Ku Han Bai Wei)*, where prolonged or excessive intake of these agents may compromise gastrointestinal homeostasis, leading to impaired digestive motility and absorption.

While our meta-analysis focused on efficacy, the safety profile of combining botanical formulations with Met requires careful consideration. Firstly, regarding adverse events: As reported in the included trials, the most frequent adverse events were gastrointestinal symptoms (e.g., diarrhea, nausea, abdominal distension). Met itself is well-known to cause gastrointestinal intolerance in up to 30% of patients. The addition of “bitter-cold” (Ku-Han) herbal formulas (e.g., HLJDD containing Coptis chinensis) may theoretically exacerbate these symptoms due to their stimulating effect on the gastrointestinal tract. However, our analysis suggests that these symptoms were generally mild and transient. We identified significant data gaps in safety reporting; only 8 RCTs (20.0%), involving 745 out of 3,088 participants, recorded adverse events. No data regarding treatment discontinuations due to adverse effects were systematically reported in the remaining 32 trials. While no severe adverse events (SAEs), such as lactic acidosis or severe hypoglycemia leading to coma, were documented in this reporting subset, the true incidence of SAEs may be obscured by the coarse reporting and small sample sizes. Therefore, there is limited and uncertain evidence to establish a definitive safety profile for these combination therapies.

The findings of this NMA should be interpreted within the context of the included study populations and healthcare settings. Since all 40 included RCTs originated from China, the evidence is primarily applicable to East Asian populations with T2DM under the Chinese healthcare system, where integrated botanical-biomedical management is routine. Given known ethnic differences in T2DM pathophysiology (e.g., differences in beta-cell function and BMI thresholds between Asian and Caucasian populations) and potential genetic variations in drug Met (e.g., polymorphisms affecting Met transporters), caution is warranted when extrapolating these results to non-Asian populations. Future multi-center international trials are necessary to validate the efficacy and safety of these botanical formulas in diverse ethnic groups and Western clinical settings.

In summary, our study indicates that specific botanical formulas when combined with Met, are likely associated with improved glycemic and lipid control compared to Met monotherapy. However, it is crucial to interpret these rankings with caution. The network structure is predominantly star-shaped, meaning that the relative effects between herbal formulas rely heavily on indirect evidence and transitivity assumptions rather than head-to-head clinical trials. Consequently, the rankings are exploratory and may be subject to instability, particularly for comparisons supported by sparse evidence or low certainty. A high SUCRA score determines the probability of a treatment being best but does not quantify the magnitude of difference or the certainty of evidence. As shown in our GRADE assessment, the certainty of evidence for many head-to-head comparisons was graded as very low, mainly limiting the robustness of these rankings. Furthermore, to account for the risk of false-positive findings associated with multiple comparisons, we interpreted our secondary outcomes (e.g., lipid profiles and insulin parameters) as exploratory. These findings should be viewed as preliminary evidence that requires validation in pre-specified, large-scale clinical trials. Thus, these findings should be viewed as suggestive of a hierarchy of efficacy, identifying promising candidates for future high-quality trials, rather than confirmatory evidence.

### Advantages

Firstly, this systematic review strictly adhered to the guidelines outlined in the PRISMA-NMA. Furthermore, to ensure comprehensive retrieval, we searched eight electronic databases, encompassing both Chinese and English literature. To comply with the gold standard for clinical trials, namely RCTs, we excluded any RCTs failing to meet essential eligibility criteria or non-randomized controlled trials that could potentially bias our findings. A Bayesian multi-treatment NMA was employed, yielding more precise estimates compared to frequentist approaches. Finally, the network estimates were robust, as all P values from the node-splitting analysis for inconsistency exceeded 0.05, and all curves from the convergence diagnostics approached 1, indicating good model fit.

### Limitations

Several limitations warrant consideration. First, at the study level, most included RCTs were limited by small sample sizes, short durations, and methodological weaknesses—particularly regarding allocation concealment and blinding. While biochemical outcomes are less prone to placebo effects, the lack of blinding introduces performance bias. We addressed this by strictly applying the GRADE framework to downgrade the certainty of evidence to “Very Low” for most comparisons, rather than excluding studies, which would have led to network disconnection and loss of statistical power.

Second, regarding the scope of evidence, the exclusive focus on Chinese studies limits the geographical generalizability of our findings. Additionally, while the inclusion of master’s theses mitigated publication bias, their lack of traditional peer review further contributed to lower evidence certainty. The safety profile also remains preliminary, as 80% of trials lacked standardized reporting on AEs/SAEs, potentially underestimating potential risks.

Third, the network’s predominantly star-shaped structure—lacking head-to-head trials between herbal formulas—represents a significant systematic limitation. This reliance on indirect evidence through a common control (Met) means that the comparative effectiveness and the resulting SUCRA rankings are exploratory rather than confirmatory, and may be subject to instability. Consequently, these findings should be interpreted with caution, and future large-scale, double-blind, head-to-head RCTs are required to provide more robust evidence for clinical decision-making.

## Data Availability

The original contributions presented in the study are included in the article/Supplementary Material, further inquiries can be directed to the corresponding author.
