# Supplementary material for: Efficacy and safety of traditional Chinese classic prescriptions combined with metformin in the treatment of type 2 diabetes mellitus: a Bayesian network meta-analysis
Source: Front Pharmacol. 2026 Feb 11;17:1693378. doi: 10.3389/fphar.2026.1693378 (PMC12932438; doi:10.3389/fphar.2026.1693378)
Supplement: Supplementary file 1 [file DataSheet7.pdf]

Full League Table (HbA1c, ≥ 8 Weeks)

	**HLJDD**	**ZBDHD**	**DCHD**	**GGQLD**	**HLWDD**	**SLBZD**	**LGZGD**	**BHRSD**	**Met**
**HLJDD**	**HLJDD**	-0.00 (-0.45, 0.44)	-0.06 (-0.67, 0.54)	-0.27 (-0.65, 0.11)	-0.28 (-0.74, 0.19)	-0.29 (-0.68, 0.11)	-0.43 (-0.84, -0.03)	-0.70 (-1.08, -0.32)	-1.02 (-1.39, -0.66)
**ZBDHD**	0.00 (-0.44, 0.45)	**ZBDHD**	-0.06 (-0.61, 0.49)	-0.26 (-0.55, 0.02)	-0.27 (-0.66, 0.12)	-0.28 (-0.59, 0.03)	-0.43 (-0.75, -0.11)	-0.70 (-0.99, -0.41)	-1.02 (-1.28, -0.76)
**DCHD**	0.06 (-0.54, 0.67)	0.06 (-0.49, 0.61)	**DCHD**	-0.20 (-0.70, 0.30)	-0.21 (-0.78, 0.36)	-0.22 (-0.73, 0.29)	-0.37 (-0.89, 0.15)	-0.64 (-1.14, -0.13)	-0.96 (-1.45, -0.47)
**GGQLD**	0.27 (-0.11, 0.65)	0.26 (-0.02, 0.55)	0.20 (-0.30, 0.70)	**GGQLD**	-0.01 (-0.32, 0.30)	-0.02 (-0.22, 0.18)	-0.16 (-0.38, 0.05)	-0.44 (-0.61, -0.26)	-0.76 (-0.87, -0.64)
**HLWDD**	0.28 (-0.19, 0.74)	0.27 (-0.12, 0.66)	0.21 (-0.36, 0.78)	0.01 (-0.30, 0.32)	**HLWDD**	-0.01 (-0.34, 0.32)	-0.15 (-0.50, 0.19)	-0.43 (-0.74, -0.11)	-0.75 (-1.04, -0.46)
**SLBZD**	0.29 (-0.11, 0.68)	0.28 (-0.03, 0.59)	0.22 (-0.29, 0.73)	0.02 (-0.18, 0.22)	0.01 (-0.32, 0.34)	**SLBZD**	-0.15 (-0.39, 0.10)	-0.42 (-0.63, -0.21)	-0.74 (-0.90, -0.57)
**LGZGD**	0.43 (0.03, 0.84)	0.43 (0.11, 0.75)	0.37 (-0.15, 0.89)	0.16 (-0.05, 0.38)	0.15 (-0.19, 0.50)	0.15 (-0.10, 0.39)	**LGZGD**	-0.27 (-0.50, -0.05)	-0.59 (-0.78, -0.41)
**BHRSD**	0.70 (0.32, 1.08)	0.70 (0.41, 0.99)	0.64 (0.13, 1.14)	0.44 (0.26, 0.61)	0.43 (0.11, 0.74)	0.42 (0.21, 0.63)	0.27 (0.05, 0.50)	**BHRSD**	-0.32 (-0.45, -0.19)
**Met**	1.02 (0.66, 1.39)	1.02 (0.76, 1.28)	0.96 (0.47, 1.45)	0.76 (0.64, 0.87)	0.75 (0.46, 1.04)	0.74 (0.57, 0.90)	0.59 (0.41, 0.78)	0.32 (0.19, 0.45)	**Met**

Sensitivity Analysis Ranking Table (HbA1c, ≥ 8 Weeks)

Rank	Intervention	Mean Difference (vs. Met)	95% Credible Intervals (CrIs)
1	HLJDD + Met	-1.02	(-1.39, -0.66)
2	ZBDHD + Met	-1.02	(-1.28, -0.76)
3	DCHD + Met	-0.96	(-1.45, -0.47)
4	GGQLD + Met	-0.76	(-0.87, -0.64)
5	HLWDD + Met	-0.75	(-1.04, -0.46)
6	SLBZD + Met	-0.74	(-0.90, -0.57)
7	LGZGD + Met	-0.59	(-0.78, -0.41)
8	BHRSD + Met	-0.32	(-0.45, -0.19)
9	Metformin (Ref)	0	(Reference)

Intervention	Primary Rank (All)	Sensitivity Rank (≥ 8w)	Change
HLJDD + Met	2	1	↑
ZBDHD + Met	3	2	↑
DCHD + Met	1	3	↓
