# Supplementary material for: Efficacy and safety of traditional Chinese classic prescriptions combined with metformin in the treatment of type 2 diabetes mellitus: a Bayesian network meta-analysis
Source: Front Pharmacol. 2026 Feb 11;17:1693378. doi: 10.3389/fphar.2026.1693378 (PMC12932438; doi:10.3389/fphar.2026.1693378)
Supplement: Supplementary file 2 [file DataSheet13.pdf]

study	treatment	mean	std.dev	sampleSize	risk of bias	Intervention time	sample size	Improved herbal
Shaowu Zeng	SLBZP	-2.52	1.116646766	40	0	1	1	1
Shaowu Zeng	Met	-2	1.202538981	40	0	1	1	1
Yuanchun Chen	ZBDHD	-2.82	1.155032467	50	0	1	1	1
Yuanchun Chen	Met	-2.26	1.231584345	50	0	1	1	1
Sisi Chen	HLJDD	-2.85	2.120542383	50	0	1	1	0
Sisi Chen	Met	-1.37	2.577343594	49	0	1	1	0
Xia Chen	HLWDD	-2.67	1.134768699	30	0	1	1	0
Xia Chen	Met	-1.64	1.07196082	30	0	1	1	0
Ye Chen	HLWDD	-4.3	1.378296049	43	0	0	1	1
Ye Chen	Met	-2.67	1.508011936	43	0	0	1	1
Mengjie Cheng	GGQLD	-1.966	0.956035564	30	0	1	1	0
Mengjie Cheng	Met	-1.18	0.813459895	30	0	1	1	0
Hongyan Cui	DCHD	-3.21	1.801360597	60	0	1	0	0
Hongyan Cui	Met	-2.85	1.708186173	60	0	1	0	0
Guoling Dai	GGQLD	-3.35	1.123031611	39	0	1	1	1
Guoling Dai	Met	-0.77	1.109639581	39	0	1	1	1
Jiajun Feng	HLJDD	-3.29	3.137307763	45	0	0	1	1
Jiajun Feng	Met	-1.72	3.319457787	45	0	0	1	1
Zhanrong Feng	BHRSD	-1.77	1.080601684	30	0	0	1	1
Zhanrong Feng	Met	-0.85	1.175925168	30	0	0	1	1
Zhaolan Feng	SLBZP	-2.5	1.135781669	32	0	1	1	0
Zhaolan Feng	Met	-2.1	1.389244399	32	0	1	1	0
Guizhen Fu	GGQLD	-2.77	0.926660671	30	0	1	1	1
Guizhen Fu	Met	-1.76	0.827103379	30	0	1	1	1
Ying Gong	SLBZP	-2.3	1.081665383	31	0	1	1	0
Ying Gong	Met	-1.9	1.3	34	0	1	1	0
Shangshang Pan	HLWDD	-6.34	3.00291525	41	0	0	1	0
Shangshang Pan	Met	-3.45	2.859527933	39	0	0	1	0
Guanghui Ji	HLWDD	-2.06	1.52561463	30	0	1	1	0
Guanghui Ji	Met	-1.07	1.860322553	30	0	1	1	0
Juliang Ji	DCHD	-3.77	3.260184044	20	0	1	1	0
Juliang Ji	Met	-2.77	4.053307292	20	0	1	1	0
Hua Li	GGQLD	-2.8	0.781024968	48	0	1	1	1
Hua Li	Met	-1.4	0.754983444	48	0	1	1	1
Jinhua Li	LGZGD	-1.03	0.761774245	30	0	1	1	0
Jinhua Li	Met	-0.25	0.749866655	30	0	1	1	0
Juanjuan Li	ZBDHD	-3.83	1.489865766	31	0	0	1	1
Juanjuan Li	Met	-2.97	1.36429469	31	0	0	1	1
Houce Liang	LGZGD	-1.55	0.673572565	38	0	1	1	0

Houce Liang	Met	-0.91	0.612209115	38	0	1	1	0
Qin Li	LGZGD	-1.48	1.03058236	40	0	0	1	1
Qin Li	Met	-1.13	1.145818485	40	0	0	1	1
Xuelin Luo	ZBDHD	-4	3.685254401	52	0	1	0	1
Xuelin Luo	Met	-3.78	3.428162773	50	0	1	0	1
Linna Ma	GGQLD	-2.37	1.498299036	55	0	1	0	1
Linna Ma	Met	-1.91	1.493686714	55	0	1	0	1
Shaolin Peng	BHRSD	-2.59	0.48754487	30	0	0	1	1
Shaolin Peng	Met	-1.79	0.424381903	30	0	0	1	1
Yanhong Rong	BHRSD	-2.2	1.126942767	40	1	1	1	0
Yanhong Rong	Met	-1.6	1.307669683	40	1	1	1	0
Qin Tan	SLBZP	-1.21	0.626019169	30	1	1	1	1
Qin Tan	Met	-0.68	0.867121675	30	1	1	1	1
Qin Tan	SLBZP	-0.66	1.455060136	100	1	1	0	1
Qin Tan	Met	-0.64	1.697527614	100	1	1	0	1
Dongmei Wang	DCHD	-4.28	1.951384124	40	0	1	1	0
Dongmei Wang	Met	-1.74	2.014770458	40	0	1	1	0
Lei Wang	GGQLD	-2.81	0.588132638	50	0	0	1	0
Lei Wang	Met	-2.13	0.54249424	50	0	0	1	0
Mingkun Wang	HLWDD	-2.73	2.387278786	30	0	1	1	1
Mingkun Wang	Met	-1.35	2.373099239	30	0	1	1	1
Yan Wang	HLWDD	-3.9	2.154646143	25	0	0	1	1
Yan Wang	Met	-3.15	2.086240638	25	0	0	1	1
Li Wu	GGQLD	-1.95	0.729588925	27	0	1	1	1
Li Wu	Met	-1.25	0.529150262	29	0	1	1	1
Fangyi Xie	GGQLD	-5.34	0.618304132	20	0	1	1	1
Fangyi Xie	Met	-2.89	0.76072334	20	0	1	1	1
Wenjun Yang	HLJDD	-8.2	1.884223978	33	0	0	1	1
Wenjun Yang	Met	-6.96	1.555602777	33	0	0	1	1
Xueqin Yang	GGQLD	-2.19	0.896046874	33	0	1	1	0
Xueqin Yang	Met	-0.76	0.461194102	33	0	1	1	0
Huiling Yu	GGQLD	-5.44	0.591523457	45	0	1	1	0
Huiling Yu	Met	-2.66	0.815046011	45	0	1	1	0
Hongguo Yuan	SLBZP	-2.09	0.692026011	43	0	0	1	1
Hongguo Yuan	Met	-1.49	0.707672241	43	0	0	1	1
Lili Zhang	HLJDD	-2.1	1.81934054	37	0	1	1	0
Lili Zhang	Met	-0.5	2.1	37	0	1	1	0
Siying Zhang	GGQLD	-1.54	0.675795827	36	0	0	1	1
Siying Zhang	Met	-0.89	0.225388553	36	0	0	1	1
Yuting Zhou	DCHD	-2.8	1.558845727	30	0	1	1	0

Yuting Zhou	Met	-1.2	1.705872211	30	0	1	1	0

study	treatment	mean	std.dev	sampleSize	risk of bias	Intervention time	sample size	Improved herbal
Shaowu Zeng	SLBZD	-4.96	1.415238496	40	0	1	1	1
Shaowu Zeng	Met	-3.67	1.28852629	40	0	1	1	1
Yuanchun Chen	ZBDHD	-4.54	2.140630748	50	0	1	1	1
Yuanchun Chen	Met	-3.88	2.298956285	50	0	1	1	1
Sisi Chen	HLJDD	-3.14	2.608505319	50	0	1	1	0
Sisi Chen	Met	-1.29	3.307007711	49	0	1	1	0
Xia Chen	HLWDD	-2.68	1.217661694	30	0	1	1	0
Xia Chen	Met	-1.74	1.188233984	30	0	1	1	0
Ye Chen	HLWDD	-5.98	1.478276023	43	0	0	1	1
Ye Chen	Met	-4.45	1.617807158	43	0	0	1	1
Mengjie Cheng	GGQLD	-4.827	1.575615435	30	0	1	1	0
Mengjie Cheng	Met	-3.767	1.576679105	30	0	1	1	0
Hongyan Cui	DCHD	-2.81	1.646481096	60	0	1	0	0
Hongyan Cui	Met	-2.61	1.929222641	60	0	1	0	0
Guoling Dai	GGQLD	-3.81	1.257417989	39	0	1	1	1
Guoling Dai	Met	-3.2	1.456159332	39	0	1	1	1
Zhanrong Feng	BHRSD	-1.97	1.893884896	30	0	0	1	1
Zhanrong Feng	Met	-1.66	2.165155884	30	0	0	1	1
Zhaolan Feng	SLBZD	-3.8	1.311487705	32	0	1	1	0
Zhaolan Feng	Met	-3.5	1.1	32	0	1	1	0
Guizhen Fu	GGQLD	-3.07	1.035905401	30	0	1	1	1
Guizhen Fu	Met	-1.75	1.069439105	30	0	1	1	1
Ying Gong	SLBZD	-3.6	1.3	31	0	1	1	0
Ying Gong	Met	-3.5	1.113552873	34	0	1	1	0
Guanghui Ji	HLWDD	-4.16	2.553194078	30	0	1	1	0
Guanghui Ji	Met	-2.57	2.194265253	30	0	1	1	0
Juliang Ji	DCHD	-3.9	4.268383769	20	0	1	1	0
Juliang Ji	Met	-3.21	4.914905899	20	0	1	1	0
Hua Li	GGQLD	-4.1	1.276714533	48	0	1	1	1
Hua Li	Met	-1.7	1	48	0	1	1	1
Jinhua Li	LGZGD	-0.81	2.060946385	30	0	1	1	0
Jinhua Li	Met	-0.3	2.325747192	30	0	1	1	0
Juanjuan Li	ZBDHD	-4.44	1.502697574	31	0	0	1	1
Juanjuan Li	Met	-2.8	1.521282354	31	0	0	1	1
Houce Liang	LGZGD	-3.1	2.42693222	38	0	1	1	0
Houce Liang	Met	-2.4	2.343074903	38	0	1	1	0
Qin Li	LGZGD	-2.36	1.724615899	40	0	0	1	1
Qin Li	Met	-1.4	1.612048386	40	0	0	1	1
Xuelin Luo	ZBDHD	-7.91	3.458959381	52	0	1	0	1

Xuelin Luo	Met	-6.23	3.83722556	50	0	1	0	1
Linna Ma	GGQLD	-3.8	1.919400948	55	0	1	0	1
Linna Ma	Met	-2.67	1.96	55	0	1	0	1
Shaolin Peng	BHRSD	-4.1	0.509215082	30	0	0	1	1
Shaolin Peng	Met	-3.43	0.485077313	30	0	0	1	1
Yanhong Rong	BHRSD	-4.4	3.290896534	40	1	1	1	0
Yanhong Rong	Met	-2.9	3.538361203	40	1	1	1	0
Qin Tan	SLBZD	-0.75	1.159353268	30	1	1	1	1
Qin Tan	Met	-0.54	0.751065909	30	1	1	1	1
Qin Tan	SLBZD	-1.05	1.758379936	100	1	1	0	1
Qin Tan	Met	-0.73	1.93450252	100	1	1	0	1
Dongmei Wang	DCHD	-5.06	1.888597363	40	0	1	1	0
Dongmei Wang	Met	-1.79	1.928289397	40	0	1	1	0
Lei Wang	GGQLD	-4.55	1.102859919	50	0	0	1	0
Lei Wang	Met	-3.34	1.116109314	50	0	0	1	0
Mingkun Wang	HLWDD	-5.06	2.191597591	30	0	1	1	1
Mingkun Wang	Met	-2.75	2.575829963	30	0	1	1	1
Yan Wang	HLWDD	-5.52	2.407882888	25	0	0	1	1
Yan Wang	Met	-3.27	2.33826859	25	0	0	1	1
Li Wu	GGQLD	-3.63	2.062328781	27	0	1	1	1
Li Wu	Met	-2.26	1.874326546	29	0	1	1	1
Fangyi Xie	GGQLD	-2.31	0.34	20	0	1	1	1
Fangyi Xie	Met	-0.79	1.006429332	20	0	1	1	1
Wenjun Yang	HLJDD	-11.4	2.706233545	33	0	0	1	1
Wenjun Yang	Met	-10.87	2.710940796	33	0	0	1	1
Xueqin Yang	GGQLD	-2.7	0.578186821	33	0	1	1	0
Xueqin Yang	Met	-1.25	0.606547607	33	0	1	1	0
Huiling Yu	GGQLD	-3.31	1.057875229	45	0	1	1	0
Huiling Yu	Met	-1.54	0.822860863	45	0	1	1	0
Hongguo Yuan	SLBZD	-3.25	0.907964757	43	0	0	1	1
Hongguo Yuan	Met	-2.01	0.949368211	43	0	0	1	1
Lili Zhang	HLJDD	-3.5	2.1	37	0	1	1	0
Lili Zhang	Met	-2	1.852025918	37	0	1	1	0
Siying Zhang	GGQLD	-5.18	1.426429108	36	0	0	1	1
Siying Zhang	Met	-4.52	1.528037958	36	0	0	1	1
Yuting Zhou	DCHD	-3.7	2.570992026	30	0	1	1	0
Yuting Zhou	Met	-1.9	2.88444102	30	0	1	1	0

study	treatment	mean	std.dev	sampleSize	risk of bias	Intervention time	sample size	Improved herbal
Shaowu Zeng	SLBZD	-1.49	0.52	40	0	1	1	1
Shaowu Zeng	Met	-0.99	0.445308882	40	0	1	1	1
Yuanchun Chen	ZBDHD	-2.46	1.040240357	50	0	1	1	1
Yuanchun Chen	Met	-1.77	1.090825376	50	0	1	1	1
Sisi Chen	HLJDD	-1.13	1.506386405	50	0	1	1	0
Sisi Chen	Met	-0.3	1.686801707	49	0	1	1	0
Xia Chen	HLWDD	-2.11	0.970412284	30	0	1	1	0
Xia Chen	Met	-1.32	1.024548681	30	0	1	1	0
Ye Chen	HLWDD	-3.6	1.541654955	43	0	0	1	1
Ye Chen	Met	-2.13	1.63	43	0	0	1	1
Mengjie Cheng	GGQLD	-1.776	0.498481695	30	0	1	1	0
Mengjie Cheng	Met	-1.544	0.516523959	30	0	1	1	0
Hongyan Cui	DCHD	-2.37	1.38567673	60	0	1	0	0
Hongyan Cui	Met	-1.41	1.331014651	60	0	1	0	0
Jiajun Feng	HLJDD	-2.07	1.345808307	45	0	0	1	1
Jiajun Feng	Met	-0.73	1.410957122	45	0	0	1	1
Zhanrong Feng	BHRSD	-0.77	0.936162379	30	0	0	1	1
Zhanrong Feng	Met	-0.54	0.930161276	30	0	0	1	1
Zhaolan Feng	SLBZD	-2.06	1.077636302	32	0	1	1	0
Zhaolan Feng	Met	-1.07	1.148956048	32	0	1	1	0
Guizhen Fu	GGQLD	-2.87	1.101226589	30	0	1	1	1
Guizhen Fu	Met	-1.76	1.055319857	30	0	1	1	1
Ying Gong	SLBZD	-2.04	1.075127899	31	0	1	1	0
Ying Gong	Met	-1.05	1.147649772	34	0	1	1	0
Guanghui Ji	HLWDD	-0.62	1.20503112	30	0	1	1	0
Guanghui Ji	Met	-0.6	1.240120962	30	0	1	1	0
Juliang Ji	DCHD	-3.71	1.472854372	20	0	1	1	0
Juliang Ji	Met	-1	1.094897255	20	0	1	1	0
Hua Li	GGQLD	-4.3	0.4	48	0	1	1	1
Hua Li	Met	-1.1	0.608276253	48	0	1	1	1
Juanjuan Li	ZBDHD	-3.29	1.196160524	31	0	1	1	0
Juanjuan Li	Met	-1.85	1.275656694	31	0	1	1	0
Houce Liang	LGZGD	-1	0.7	38	0	1	1	0
Houce Liang	Met	-0.3	0.6244998	38	0	1	1	0
Qin Li	LGZGD	-1.42	0.49689033	40	0	0	1	1
Qin Li	Met	-0.89	0.564269439	40	0	0	1	1
Xuelin Luo	ZBDHD	-5.11	1.03058236	52	0	1	0	1
Xuelin Luo	Met	-3.96	1.021958903	50	0	1	0	1
Linna Ma	GGQLD	-1.81	1.505822035	55	0	1	0	1
Linna Ma	Met	-1.22	1.46447943	55	0	1	0	1
Shaolin Peng	BHRSD	-1.69	0.278388218	30	0	0	1	1
Shaolin Peng	Met	-1.4	0.270554985	30	0	0	1	1
Yanhong Rong	BHRSD	-2.2	1.058300524	40	1	1	1	0

Yanhong Rong	Met	-1.4	1.1	40	1	1	1	0
Dongmei Wang	DCHD	-5.2	2.163954713	40	0	1	1	0
Dongmei Wang	Met	-3.57	2.085113906	40	0	1	1	0
Lei Wang	GGQLD	-1.97	0.613921819	50	0	0	1	0
Lei Wang	Met	-1.17	0.59405387	50	0	0	1	0
Mingkun Wang	HLWDD	-1.75	1.529836593	30	0	1	1	1
Mingkun Wang	Met	-0.66	1.71467198	30	0	1	1	1
Yan Wang	HLWDD	-1.78	1.427900557	25	0	0	1	1
Yan Wang	Met	-1.25	1.371969387	25	0	0	1	1
Li Wu	GGQLD	-1.55	0.409511905	27	0	1	1	1
Li Wu	Met	-1.06	0.504777179	29	0	1	1	1
Fangyi Xie	GGQLD	-4.59	1.013360745	20	0	1	1	1
Fangyi Xie	Met	-3.44	0.777881739	20	0	1	1	1
Wenjun Yang	HLJDD	-4.2	1.931320792	33	0	0	1	1
Wenjun Yang	Met	-2.9	2.23383079	33	0	0	1	1
Huiling Yu	GGQLD	-4.84	0.580258563	45	0	1	1	0
Huiling Yu	Met	-3.28	0.976165969	45	0	1	1	0
Hongguo Yuan	SLBZD	-3.47	0.83108363	43	0	0	1	1
Hongguo Yuan	Met	-2.27	0.855628424	43	0	0	1	1
Lili Zhang	HLJDD	-2.51	2.052632456	37	0	1	1	0
Lili Zhang	Met	-2.18	2.161110825	37	0	1	1	0
Siying Zhang	GGQLD	-1.23	0.52848841	36	0	0	1	1
Siying Zhang	Met	-0.74	0.445757782	36	0	0	1	1

study	treatment	mean	std.dev	sampleSize	risk of bias	Intervention time	sample size	Improved herbal
Shaowu Zeng	SLBZD	-2.9	0.705478561	40	0	1	1	1
Shaowu Zeng	Met	-2.03	0.631743619	40	0	1	1	1
Sisi Chen	HLJDD	-2.78	1.146429239	50	0	1	1	0
Sisi Chen	Met	-1.59	1.26676754	49	0	1	1	0
Xia Chen	HLWDD	-2.25	0.895041898	30	0	1	1	0
Xia Chen	Met	-1.93	0.935788438	30	0	1	1	0
Mengjie Cheng	GGQLD	-0.001	1.215782875	30	0	1	1	0
Mengjie Cheng	Met	-0.068	1.265929303	30	0	1	1	0
Jiajun Feng	HLJDD	-2.4	1.489865766	45	0	0	1	1
Jiajun Feng	Met	-0.81	1.47929037	45	0	0	1	1
Zhanrong Feng	BHRSD	-0.44	0.520480547	30	0	0	1	1
Zhanrong Feng	Met	-0.06	0.54064776	30	0	0	1	1
Zhaolan Feng	SLBZD	-0.89	0.692026011	32	0	1	1	0
Zhaolan Feng	Met	-0.82	0.74101282	32	0	1	1	0
Guizhen Fu	GGQLD	-1.75	0.888988189	30	0	1	1	1
Guizhen Fu	Met	-0.98	0.903714557	30	0	1	1	1
Ying Gong	SLBZD	-0.88	0.687895341	31	0	1	1	0
Ying Gong	Met	-0.8	0.73430239	34	0	1	1	0
Guanghui Ji	HLWDD	-0.54	0.638357267	30	0	1	1	0
Guanghui Ji	Met	-0.44	0.633798075	30	0	1	1	0
Jinhua Li	LGZGD	-0.63	1.235758876	30	0	1	1	0
Jinhua Li	Met	-0.35	1.217497433	30	0	1	1	0
Qin Li	LGZGD	-1.963	1.165032188	40	0	0	1	1
Qin Li	Met	-0.45	1.270472353	40	0	0	1	1
Linna Ma	GGQLD	-1.15	0.525737577	55	0	1	0	1
Linna Ma	Met	-0.5	0.516430053	55	0	1	0	1
Lei Wang	GGQLD	-1.65	0.386910842	50	0	0	1	0
Lei Wang	Met	-1.18	0.422255847	50	0	0	1	0
Mingkun Wang	HLWDD	-0.35	1.579018683	30	0	1	1	1
Mingkun Wang	Met	-0.35	1.711256848	30	0	1	1	1
Xueqin Yang	GGQLD	-2.19	0.660908466	33	0	1	1	0
Xueqin Yang	Met	-1.34	0.56559703	33	0	1	1	0
Hongguo Yuan	SLBZD	-1.27	0.48815981	43	0	0	1	1
Hongguo Yuan	Met	-0.56	0.506853036	43	0	0	1	1

study	treatment	mean	std.dev	sampleSize	risk of bias	Intervention time	sample size	Improved herbal
Shaowu Zeng	SLBZD	-0.54	0.32969683	40	0	1	1	1
Shaowu Zeng	Met	-0.34	0.320468407	40	0	1	1	1
Sisi Chen	HLJDD	-2.37	1.081295519	50	0	1	1	0
Sisi Chen	Met	-1.57	1.129026129	49	0	1	1	0
Xia Chen	HLWDD	-1.34	0.520480547	30	0	1	1	0
Xia Chen	Met	-1.26	0.883572295	30	0	1	1	0
Mengjie Cheng	GGQLD	-0.048	0.29496271	30	0	1	1	0
Mengjie Cheng	Met	-0.022	0.529436257	30	0	1	1	0
Jiajun Feng	HLJDD	-0.62	0.319530906	45	0	0	1	1
Jiajun Feng	Met	-0.15	0.326036808	45	0	0	1	1
Zhanrong Feng	BHRSD	-0.9	0.452106182	30	0	0	1	1
Zhanrong Feng	Met	-0.94	0.570876519	30	0	0	1	1
Zhaolan Feng	SLBZD	-1.27	0.418688428	32	0	1	1	0
Zhaolan Feng	Met	-1.39	0.399499687	32	0	1	1	0
Guizhen Fu	GGQLD	-1.16	0.838629835	30	0	1	1	1
Guizhen Fu	Met	-0.69	0.886397202	30	0	1	1	1
Ying Gong	SLBZD	-1.25	0.413884042	31	0	1	1	0
Ying Gong	Met	-1.37	0.392300905	34	0	1	1	0
Guanghui Ji	HLWDD	-1.02	1.212476804	30	0	1	1	0
Guanghui Ji	Met	-0.43	0.826135582	30	0	1	1	0
Jinhua Li	LGZGD	-0.62	1.430524379	30	0	1	1	0
Jinhua Li	Met	-0.73	1.570095538	30	0	1	1	0
Qin Li	LGZGD	-2.34	0.835404094	40	0	0	1	1
Qin Li	Met	-0.59	0.87846457	40	0	0	1	1
Lei Wang	GGQLD	-0.65	0.174355958	50	0	0	1	0
Lei Wang	Met	-0.33	0.173493516	50	0	0	1	0
Mingkun Wang	HLWDD	-0.87	1.092336944	30	0	1	1	1
Mingkun Wang	Met	-0.49	0.874128137	30	0	1	1	1
Xueqin Yang	GGQLD	-1.24	0.58966092	33	0	1	1	0
Xueqin Yang	Met	-0.65	0.863770803	33	0	1	1	0
Hongguo Yuan	SLBZD	-1.11	0.244335834	43	0	0	1	1
Hongguo Yuan	Met	-0.55	0.248797106	43	0	0	1	1
Yuting Zhou	DCHD	-0.91	0.502692749	30	0	1	1	0
Yuting Zhou	Met	-0.6	0.54064776	30	0	1	1	0

study	treatment	mean	std.dev	sampleSize	risk of bias	Intervention time	sample size	Improved herbal
Shaowu Zeng	SLBZD	-2.43	0.827103379	40	0	1	1	1
Shaowu Zeng	Met	-2.05	0.711055553	40	0	1	1	1
Sisi Chen	HLJDD	-1.41	0.398371686	50	0	1	1	0
Sisi Chen	Met	-0.49	0.428835633	49	0	1	1	0
Xia Chen	HLWDD	-1.78	0.679485099	30	0	1	1	0
Xia Chen	Met	-1.22	0.674462749	30	0	1	1	0
Jiajun Feng	HLJDD	-2.31	1.11216006	45	0	0	1	1
Jiajun Feng	Met	-0.61	1.185284776	45	0	0	1	1
Zhanrong Feng	BHRSD	-0.48	0.226495033	30	0	0	1	1
Zhanrong Feng	Met	-0.32	0.374032084	30	0	0	1	1
Guizhen Fu	GGQLD	-1.11	0.736681749	30	0	1	1	1
Guizhen Fu	Met	-0.76	0.995791143	30	0	1	1	1
Guanghui Ji	HLWDD	-0.46	0.399624824	30	0	1	1	0
Guanghui Ji	Met	-0.73	0.396862697	30	0	1	1	0
Jinhua Li	LGZGD	-0.53	1.206109448	30	0	1	1	0
Jinhua Li	Met	-0.15	1.10761004	30	0	1	1	0
Qin Li	LGZGD	-0.61	0.635924524	40	0	0	1	1
Qin Li	Met	-0.38	0.747462374	40	0	0	1	1
Linna Ma	GGQLD	-0.71	0.461194102	55	0	1	0	1
Linna Ma	Met	-0.35	0.457383865	55	0	1	0	1
Lei Wang	GGQLD	-1.44	0.289309523	50	0	1	1	0
Lei Wang	Met	-1.02	0.29866369	50	0	0	1	0
Mingkun Wang	HLWDD	-1.02	1.463181465	30	0	1	1	1
Mingkun Wang	Met	-0.34	1.294102005	30	0	1	1	1
Xueqin Yang	GGQLD	-1.01	0.790506167	33	0	1	1	0
Xueqin Yang	Met	-0.33	0.860290649	33	0	1	1	0
Hongguo Yuan	SLBZD	-1.27	0.326955654	43	0	0	1	1
Hongguo Yuan	Met	-0.83	0.327414111	43	0	0	1	1
Yuting Zhou	DCHD	-0.98	0.825287829	30	0	1	1	0
Yuting Zhou	Met	-0.68	0.918041393	30	0	1	1	0

study	treatment	mean	std.dev	sampleSize	risk of bias	Intervention time	sample size	Improved herbal
Sisi Chen	HLJDD	0.65	0.365102725	50	0	1	1	0
Sisi Chen	Met	0.31	0.21	49	0	1	1	0
Jiajun Feng	HLJDD	0.66	0.326955654	45	0	0	1	1
Jiajun Feng	Met	0.32	0.278747197	45	0	0	1	1
Zhanrong Feng	BHRSD	0.24	0.132287566	30	0	0	1	1
Zhanrong Feng	Met	0.14	0.14	30	0	0	1	1
Guizhen Fu	GGQLD	1.08	0.37	30	0	1	1	1
Guizhen Fu	Met	0.62	0.204205779	30	0	1	1	1
Guanghui Ji	HLWDD	0.27	0.324191302	30	0	1	1	0
Guanghui Ji	Met	0.14	0.17	30	0	1	1	0
Qin Li	LGZGD	0.25	0.240208243	40	0	0	1	1
Qin Li	Met	0.2	0.223383079	40	0	0	1	1
Lei Wang	GGQLD	0.35	0.127671453	50	0	1	1	0
Lei Wang	Met	0.21	0.111355287	50	0	0	1	0
Mingkun Wang	HLWDD	0.03	0.707601583	30	0	1	1	1
Mingkun Wang	Met	-0.18	0.715052446	30	0	1	1	1
Xueqin Yang	GGQLD	0.4	0.389743505	33	0	1	1	0
Xueqin Yang	Met	0.04	0.326955654	33	0	1	1	0
Yuting Zhou	DCHD	0.14	0.245153013	30	0	1	1	0
Yuting Zhou	Met	0.1	0.22	30	0	1	1	0

study	treatment	mean	std.dev	sampleSize	risk of bias	Intervention time	sample size	Improved herbal
Sisi Chen	HLJDD	-2.79	4.191288585	50	0	1	1	0
Sisi Chen	Met	-1.05	5.377071694	49	0	1	1	0
Xia Chen	HLWDD	-5.71	2.453915239	30	0	1	1	0
Xia Chen	Met	-2.76	4.265008792	30	0	1	1	0
Ye Chen	HLWDD	-3.92	1.262972684	43	0	0	1	1
Ye Chen	Met	-2	1.33502809	43	0	0	1	1
Jiajun Feng	HLJDD	-2.3	4.941730466	45	0	0	1	1
Jiajun Feng	Met	-2.23	4.912311065	45	0	0	1	1
Zhaolan Feng	SLBZP	-11.1	6.98641539	32	0	1	1	0
Zhaolan Feng	Met	-4.1	6.802205525	32	0	1	1	0
Guizhen Fu	GGQLD	-5.4	0.854224795	30	0	1	1	1
Guizhen Fu	Met	-2.35	0.835942582	30	0	1	1	1
Ying Gong	SLBZP	-10.9	6.921704992	31	0	1	1	0
Ying Gong	Met	-3.9	6.702238432	34	0	1	1	0
Shangshang Pan	HLWDD	-6.03	2.950101693	41	0	0	1	0
Shangshang Pan	Met	-3.48	2.880989413	39	0	0	1	0
Guanghui Ji	HLWDD	-4.62	4.861491541	30	0	1	1	0
Guanghui Ji	Met	-2.14	2.600922913	30	0	1	1	0
Juanjuan Li	ZBDHD	-15.92	3.72810944	31	0	1	1	0
Juanjuan Li	Met	-6.13	3.892081705	31	0	1	1	0
Qin Li	LGZGD	-6.39	4.552109401	40	0	0	1	1
Qin Li	Met	-5.57	4.97075447	40	0	0	1	1
Lei Wang	GGQLD	-6.97	1.385243661	50	0	0	1	0
Lei Wang	Met	-4.86	1.285029183	50	0	0	1	0
Mingkun Wang	HLWDD	-7.96	6.433917935	30	0	1	1	1
Mingkun Wang	Met	-1.15	6.735272823	30	0	1	1	1
Li Wu	GGQLD	-3.93	4.416525784	27	0	1	1	1
Li Wu	Met	-1.6	4.23982311	29	0	1	1	1
Siying Zhang	GGQLD	-6.02	1.890317434	36	0	0	1	1
Siying Zhang	Met	-4.87	1.697409791	36	0	0	1	1

study	treatment	mean	std.dev	sampleSize	risk of bias	Intervention time	sample size	Improved herbal
Yuanchun Chen	ZBDHD	-1.19	1.681457701	50	0	1	1	1
Yuanchun Chen	Met	-0.78	2.019628679	50	0	1	1	1
Sisi Chen	HLJDD	-2.41	1.907170679	50	0	1	1	0
Sisi Chen	Met	-1.01	2.719724251	49	0	1	1	0
Zhaolan Feng	SLBZD	-6.8	1.479864859	32	0	1	1	0
Zhaolan Feng	Met	-5.7	2.25166605	32	0	1	1	0
Guizhen Fu	GGQLD	-3.83	0.788098979	30	0	1	1	1
Guizhen Fu	Met	-2.25	0.781024968	30	0	1	1	1
Ying Gong	SLBZD	-6.6	1.385640646	31	0	1	1	0
Ying Gong	Met	-5.5	2.165640783	34	0	1	1	0
Shangshang Pan	HLWDD	-1.04	0.897719333	41	0	0	1	0
Shangshang Pan	Met	-0.47	1.9832549	39	0	0	1	0
Guanghui Ji	HLWDD	-1.64	1.683062685	30	0	1	1	0
Guanghui Ji	Met	-1.32	2.469028149	30	0	1	1	0
Juanjuan Li	ZBDHD	-9.96	1.868769649	31	0	0	1	1
Juanjuan Li	Met	-5.93	1.846591455	31	0	0	1	1
Qin Li	LGZGD	-3.61	2.79649781	40	0	0	1	1
Qin Li	Met	-2.19	3.017101258	40	0	0	1	1
Linna Ma	GGQLD	-30.47	4.021504694	55	0	1	0	1
Linna Ma	Met	-15.44	4.132541591	55	0	1	0	1
Lei Wang	GGQLD	-3.12	0.476969601	50	0	0	1	0
Lei Wang	Met	-2.21	0.461302504	50	0	0	1	0
Li Wu	GGQLD	-2.39	1.433561997	27	0	1	1	1
Li Wu	Met	-1.39	1.676275634	29	0	1	1	1
Fangyi Xie	GGQLD	-1.29	0.594306318	20	0	1	1	1
Fangyi Xie	Met	-0.69	0.668356192	20	0	1	1	1
Wenjun Yang	HLJDD	-0.94	0.61245408	33	0	0	1	1
Wenjun Yang	Met	-0.16	0.57	33	0	0	1	1
Hongguo Yuan	SLBZD	-3.11	0.637887137	43	0	0	1	1
Hongguo Yuan	Met	-2.02	0.668430999	43	0	0	1	1
Siying Zhang	GGQLD	-8.87	3.730509349	36	0	0	1	1
Siying Zhang	Met	-7.78	3.845789906	36	0	0	1	1
Yuting Zhou	DCHD	-1.1	0.7	30	0	1	1	0
Yuting Zhou	Met	-0.4	1.058300524	30	0	1	1	0

```
#做贝叶斯NMA我们需要用到2个R包，一个是gemtc包（做NMA的本体），另一个是rjags包（一种Gibbs采样器）
#其中rjags包需要先安装rjags软件，然后下载rjags包才能使用
install.packages("gemtc")#安装gemtc包，做贝叶斯NMA的本体
install.packages("rjags")#安装rjags包，一个Gibbs采样器，需要同时安装rjags软件
library("gemtc")#加载gemtc包
library("rjags")#加载rjags包
library("dmetar")#最好是有这个包，下载方式见前面的两两比较meta分析
library("showtext")
showtext_auto()
#设置工作路径，设置工作路径有2种办法，一种是直接把代码文件和数据放在同一个文件夹，另一种就是用下面的setwd()命令
setwd("C:\\Users\\cheems\\Desktop\\贝叶斯NMA\\连续性变量")
getwd()
#读取数据，<-在R中是赋值（定义）的意思，快捷键是ALT+-。所以下面这行代码的意思是“读取数据contion.csv，并将这份数据赋值给一个叫nmadata 的对象”
nmadata <- read.csv("weight.csv",header = T,sep = ",")
#R语言无法识别中文和一些特殊符号，所以建议数据中干预方式均使用数字代替，然后使用下面的命令设置干预方式的名称
treatments <- read.table(textConnection(
  'id description
  1 "Gr"
  2 "Tr"
  3 "St"
  4 "Ce"
   '), header=T)
#将数据打包成gemtc可以识别分析的格式，下面这段代码的意思是“将nmadata和treatments打包进network”
network <- mtc.network(nmadata, #前面说过了，我们将数据文件读取后赋值给了nmadata
                       treatments = treatments)#前面说了，我们将干预方式命名然后赋值给了treatments
summary(network)#查看数据概要
#绘制网络证据图，这个图建议用stata绘制更好看，后面我会教怎么画
plot(network,#network是前面我们打包后的数据
     use.description = T,#表示是否使用我们定义在treatments里的标签
     vertex.label.cex=2,#表示干预方式文字大小的倍数
     vertex.size=nmadata$sampleSize, #表示将节点的大小设置为该干预方式对应的样本量，R中我们可以使用“数据集$列名“提取特定某列数据
     vertex.shape="circle", #节点的形状设置为圆形
     vertex.label.color="#226E9C", #标签的颜色，想换颜色的话改成颜色的十进制代码就行，去配色网站自己查
     vertex.label.dist=4, #设置顶点标签的距离
     vertex.label.degree=-pi/2, #设置顶点标签的角度
     vertex.color="green", #设置节点的颜色
     dynamic.edge.width=T,#将连线用直接比较的数量加权
     edge.color="gray", #将连线的颜色设置为灰色
     vertex.label.font=1)#设置顶点标签的字体类型
#建立贝叶斯模型，下面这段代码我强烈建议使用“?mtc.model”看看说明书，非常重要
#mtc.model函数是我们设置贝叶斯NMA的第一步，课件里我们说过贝叶斯需要设置先验信息，但是先验信息很多时候是没有的，设置起来难度也很大
#所以我们干脆直接使用mtc.model的默认设置“无信息先验”，让程序从我们的数据中自动总结信息
#mtc.model函数设置先验信息的命令是om.scale、hy.prior、re.prior.sd，感兴趣的话自己看看说明书研究下
```

```
model.ran <- mtc.model(network,#network是前面我们打包后的数据
                       n.chain=4,#n.chain用于设置马尔可夫链的数量，设置一般认为3-4就可以
                       likelihood="normal", #MD/SMD就设置normal，OR就设置binom,RR设置binom，HR设置poisson，HR（每组随访时间相等）设置binom
                       link="identity",#MD/SMD就设置identity，OR就设置logit,RR设置clog，HR设置log，HR（每组随访时间相等）设置cloglog
                       type="consistency", #type表示我们这里使用的是一致性模型（consistency），如果是不一致模型就设置（ume、use），回归模型就设置为（regression）
                       linearModel='random',#linearModel用于设置分析模型，fixed固定效应，random随机效应
                       dic=TRUE)#DIC表示是否输出DIC值
#对贝叶斯模型设置马尔科夫链蒙特卡洛抽样，记住下面的代码，我们现在把NMA模型赋值给了result.ran这个对象
result.ran <- mtc.run(model.ran,#model.ran是上一步我们设置的贝叶斯模型
                      sampler = "JAGS",#JAGS是一种Gibbs采样器，需要先安装rjags软件
                      n.adapt = 20000, #n.adapt设置退火次数，我个人习惯是设置20000，然后根据轨迹图密度图等改进
                      n.iter = 50000, #n.iter设置迭代次数，我个人习惯是设置50000，然后根据轨迹图密度图等改进
                      thin = 1)
summary(result.ran)#查看运行结果
#评估模型收敛情况——潜在尺度收缩因子（PSRF）
gelman.diag(result.ran)
#评估模型收敛情况——收敛诊断图（Brooks-Gelman-Rubin）
gelman.plot(result.ran)
pdf("收敛诊断图.PDF")
gelman.plot(result.ran)
dev.off()
#评估模型收敛情况——轨迹图和密度图
plot(result.ran)
pdf("轨迹图与密度图.PDF")
plot(result.ran)
dev.off()
#还有一种评估模型拟合的方法，原理是后验残差生成残差图（杠杆图）
plot(mtc.deviance(result.ran))#绘制残差图
pdf("杠杆图.PDF")
plot(mtc.deviance(result.ran))
dev.off()
#两两比较结果
#联赛表
relative.effect.table(result.ran)#查看联赛表
ls <- round(relative.effect.table(result.ran),digits  = 2)#联赛表小数位数太多，我们使用round函数设置只保留2位小数（digits = 2）
print(ls)
write.csv(ls, "联赛表.csv")#将联赛表导出为csv
#森林图,我们可以通过写for循环，实现一次性输出所有森林图
pdf("森林图.pdf",6.5,3)
for (i in network$treatments$id) {
  forest(relative.effect(result.ran,i),
         use.description = T)#设置是否在图中显示研究标签
}
```

```
dev.off()
#还有一种blobbogram的分组森林图，可以显示不同组之间的比较效果
blobdata <- read.csv("blob-weight.csv",header = T,sep = ",")#读取我们做分组森林图的数据， 并赋值给blobdata
blobbogram(blobdata, #上一行代码赋值的对象
           group.labels = c("Compare with Gr","Compare with Tr","Compare with St","Compare with Ce"),#这里要和数据中group的数字对应
           grouped = T,#是否按照数据中设定的group分组
           draw.no.effect =T,#是否显示无效线
           id.label = "Comparsion",#设置ID栏的标题
           ci.label="MD (95% CI)",#设置效应量栏的标题
           log.scale = F)#如果效应量为OR值等，这里设置为TRUE
dev.off()
#排序结果
ranks <- rank.probability(result.ran, preferredDirection=-1)#preferredDirection取1或-1，表示越大越好或越小越好
print(ranks)#概率排序值
sucra(ranks)#最重要的SUCRA排序值
plot(ranks,xlab='干预方式',ylab='堆积排序图')#堆积排序图
plot(ranks, ylim=c(0, 1), beside=TRUE,xlab='干预方式',ylab='堆积排序图')#分组堆积排序图
plot(sucra(ranks))#绘制SUCRA排序图
pdf("SUCRA排序图.pdf")
plot(sucra(ranks))
dev.off()
#节点分裂法判断不一致性,这里的代码应当与前面的一致性模型保持一致
result.ns <- mtc.nodesplit(network,#前面打包的gemtc格式数据
                          linearModel='random', #随机效应模型
                          n.adapt=20000, #20000次退火
                          n.iter=50000,#50000次迭代
                          thin=1,
                          n.chain=4,#4条马尔科夫链
                          likelihood="normal",#连续性数据
                          link="identity")
summary(result.ns)#查看不一致检验结果
plot(summary(result.ns))#查看不一致森林图
pdf("节点分割法.pdf")
plot(summary(result.ns))
dev.off()
#异质性检验，这里的参数设置应当和前面
resultanohe <- mtc.anohe(network,#前面打包的gemtc格式数据
                        n.adapt=20000,#20000次退火
                        n.iter=50000,#50000次迭代
                        thin=1,
                        n.chain=4,#4条马尔科夫链
                        likelihood="normal",#连续性数据
                        link="identity",
```

```
                         linearModel="random")#随机效应模型
summary(resultanohe)#查看异质性检验结果
plot(summary(resultanohe))#查看异质性检验森林图
dev.off()
pdf("异质性检验.pdf")
plot(summary(resultanohe))
dev.off()



#进行网状meta回归
nmadata.mr <- read.csv("weightmr.csv",header = T,sep = ",")#读取数据
network.mr <- mtc.network(data = nmadata,#mean、sd等数据依然是前面用的nmadata
                          studies = nmadata.mr,#nmadata.mr是我们刚刚赋值的数据
                          treatments = treatments)#表示使用前面赋值的干预方式标签
#设置回归参数
regressor <- list(coefficient='unrelated', #设置回归系数的类型,可以设置为unrelated、exchangeable、shared
                  variable='rob', #设置协变量
                  control='4')#设置对照组
#拟合回归模型
model.mr <- mtc.model(network.mr,
                       n.chain=4,
                       likelihood="normal",
                       link="identity",
                       type="regression",
                       linearModel='random',
                       dic=TRUE,
                      regressor=regressor)
#运行贝叶斯分析
results.mr<- mtc.run(model.mr,
                     sampler = "JAGS",
                     n.adapt = 20000,
                     n.iter = 50000,
                     thin = 1)
summary(results.mr)#查看回归模型结果
#回归分析的图形结果
par(mfrow = c(2, 2))#由于生成的图太多，所以我选择使用这行命令将图片2*2排列
plotCovariateEffect(results.mr,t1='4', t2=c('1', '2', '3'))#t1表示以谁为对照，t2表示谁与对照相比较\
dev.off()
#网状meta回归的结果
#两两比较结果——联赛表
round(relative.effect.table(results.mr),digits = 2)#控制协变量之后的综合结果
round(relative.effect.table(results.mr, covariate = 1),digits = 2)#仅查看协变量赋值为1时的联赛表，covariate用于设置分组依据，这个结果其实是亚组分析的结果
```

```
#两两比较结果——联森林图
forest(relative.effect(results.mr,  t1 = "4"),use.description  = TRUE)#控制协变量之后的综合森林图
title("控制协变量：rob")
dev.off()
forest(relative.effect(results.mr,  t1 = "4",  covariate  = 1),use.description  = TRUE)#仅查看协变量赋值为1时的森林图，covariate用于设置分组依据，这个结果其实是亚组分析的结果
title("控制协变量：rob=1")
dev.off()
#排序结果——SUCRA
rank.probability(results.mr,preferredDirection= -1)#控制协变量后的综合概率排序
sucra(rank.probability(results.mr,preferredDirection= -1))#控制协变量后的综合SUCRA
rank.probability(results.mr,preferredDirection= -1,covariate =1)#仅查看协变量赋值为1时的概率排序
sucra(rank.probability(results.mr,preferredDirection= -1,covariate =1))#仅查看协变量赋值为1时的SUCRA
```
