# Supplementary material for: Efficacy and safety of traditional Chinese classic prescriptions combined with metformin in the treatment of type 2 diabetes mellitus: a Bayesian network meta-analysis
Source: Front Pharmacol. 2026 Feb 11;17:1693378. doi: 10.3389/fphar.2026.1693378 (PMC12932438; doi:10.3389/fphar.2026.1693378)
Supplement: Supplementary file 3 [file DataSheet2.pdf]

Chinese herbal compound	Composition
Shenling Baizhu powder	Panax ginseng C. A. Mey., Atractylodes macrocephala Koidz., Poria cocos（Schw.）Wolf, Glycyrrhiza uralensis Fisch., Dioscorea opposita Thunb., Dolichos lablab L., Nelumbo nucifera Gaertn., Coix lacryma-jobi L.var.mayuen (Roman.) Stapf, Amomum villosum Lour., Platycodon grandiflorum（Jacq.）A.DC., Paeonia lactiflora Pall. (Gong et al., 2012;Feng, 2017), Alisma orientale（Sam.）Juzep. (Gong et al., 2012;Feng, 2017), Achyranthes bidentata Bl. (Gong et al., 2012;Feng, 2017), Salvia miltiorrhiza Bge. (Gong et al., 2012;Feng, 2017)
Zhibai Dihuang decoction	Rehmannia glutinosa Libosch., Cornus officinalis Sieb. et Zucc., Dioscorea opposita Thunb., Alisma orientale（Sam.）Juzep., Paeonia suffruticosa Andr., Poria cocos（Schw.）Wolf, Anemarrhena asphodeloides Bge., Phellodendron chinense Schneid., Coptis chinensis Franch. (Chen, 2015;Li and Feng, 2023)
Huanglian Jiedu decoction	Coptis chinensis Franch., Phellodendron chinense Schneid., Scutellaria baicalensis Georgi, Gardenia jasminoides Ellis, Ophiopogon japonicus（L.f）Ker-Gawl. (Chen and Wang, 2021), Scrophularia ningpoensis Hemsl. (Chen and Wang, 2021), Rehmannia glutinosa Libosch. (Chen and Wang, 2021), Forsythia suspensa （Thunb.） Vahl (Chen and Wang, 2021), Taraxacum mongolicum Hand. -Mazz. (Chen and Wang, 2021), Lonicera japonica Thunb. (Chen and Wang, 2021), Zingiber officinale Rosc. (Zhang, 2017)
Huanglian Wendan decoction	Coptis chinensis Franch., Pinellia ternata（Thunb.）Breit., Citrus reticulata Blanco, Poria cocos（Schw.）Wolf, Bambusa tuldoides Munro, Citrus aurantium L., Glycyrrhiza uralensis Fisch., Zingiber officinale Rosc., Ziziphus jujuba Mill., Atractylodes macrocephala Koidz., Scutellaria baicalensis Georgi (Ji, 2017;Chen, 2018), Trichosanthes kirilowii Maxim. (Ji, 2017;Chen, 2018), Curcuma phaeocaulis Val. (Chen, 2018), Fritillaria cirrhosa D.Don (Chen, 2018)), Pueraria lobata（Willd.）Ohwi (Chen, 2018), Salvia miltiorrhiza Bge. (Chen, 2018), Bupleurum chinense DC. (Ji, 2017;Chen, 2018), Rheum palmatum L. (Ji, 2017), Scutellaria baicalensis Georgi (Ji, 2017)
Gegen Qinlian decoction	Pueraria lobata（Willd.）Ohwi, Scutellaria baicalensis Georgi, Coptis chinensis Franch., Glycyrrhiza uralensis Fisch., Astragalus membranaceus（Fisch.）Bge. (Cheng, 2018;Wu, 2021), Dioscorea opposita Thunb. (Cheng, 2018), Atractylodes lancea（Thunb.）DC. (Cheng, 2018), Scrophularia ningpoensis Hemsl. (Cheng, 2018), Rehmannia glutinosa Libosch. (Cheng,

	2018), Trichosanthes kirilowii Maxim. (Cheng, 2018), Ophiopogon japonicus （L.f）Ker-Gawl. (Cheng, 2018), Poria cocos（Schw.）Wolf (Cheng, 2018;Yang, 2021), Trichosanthes kirilowii Maxim. (Wang, 2021), Salvia miltiorrhiza Bge. (Wang, 2021), Eupatorium fortunei Turcz. (Wang, 2021), Citrus aurantium L. (Wang, 2021), Pinellia ternata（Thunb.） Breit. (Wang, 2021), Zingiber officinale Rosc. (Wu, 2021;Xie, 2023), Citrus reticulata Blanco (Wu, 2021;Yu, 2023;Zhang, 2025), Codonopsis pilosula (Franch.)Nannf. (Yang, 2021), Atractylodes macrocephala Koidz. (Yang, 2021), Alisma orientale（Sam.） Juzep. (Yang, 2021), Ligustrum lucidum Ait. (Yu, 2023), Paeonia lactiflora Pall. (Yu, 2023), Amomum kravanh Pierre ex Gagnep. (Yu, 2023), Anemarrhena asphodeloides Bge. (Yu, 2023), Rheum palmatum L. (Yu, 2023), Coix lacryma-jobi L.var.mayuen (Roman.) Stapf (Yu, 2023)
Dachaihu decoction	Bupleurum chinense DC., Scutellaria baicalensis Georgi, Rheum palmatum L., Citrus aurantium L., Pinellia ternata（Thunb.） Breit., Paeonia lactiflora Pall., Ziziphus jujuba Mill., Zingiber officinale Rosc., Coptis chinensis Franch. (Cui and Chen, 2015), Pueraria lobata（Willd.）Ohwi (Cui and Chen, 2015;Wang, 2022), Gypsum (Cui and Chen, 2015), Pheretima aspergillum (E.Perrier) (Cui and Chen, 2015;Wang, 2022), Prunus persica（L.）Batsch (Cui and Chen, 2015;Wang, 2022), Anemarrhena asphodeloides Bge. (Cui and Chen, 2015), Salvia miltiorrhiza Bge. (Cui and Chen, 2015;Wang, 2022;Zhou, 2023), Cassia obtusifolia L. (Ji and Che, 2020), Atractylodes lancea（Thunb.） DC. (Ji and Che, 2020), Scrophularia ningpoensis Hemsl. (Ji and Che, 2020), Paeonia suffruticosa Andr. (Zhou, 2023), Crataegus pinnatifida Bge. (Zhou, 2023), Poria cocos（Schw.）Wolf (Zhou, 2023)
Baihu Renshen decoction	Anemarrhena asphodeloides Bge., Gypsum, Glycyrrhiza uralensis Fisch., Japonica rice, Panax ginseng C. A. Mey., Polygonatum odoratum（Mill.）Druce (Rong, 2019), Ophiopogon japonicus （L.f）Ker-Gawl. (Rong, 2019), Rehmannia glutinosa Libosch. (Rong, 2019), Trichosanthes kirilowii Maxim. (Rong, 2019)
Linggui Zhugan decoction	Poria cocos（Schw.）Wolf, Cinnamomum cassia Presl, Atractylodes macrocephala Koidz., Glycyrrhiza uralensis Fisch., Citrus reticulata Blanco (Liang and Wang, 2016;Li et al., 2023;Li et al., 2024), Pinellia ternata（Thunb.） Breit. (Liang and Wang, 2016;Li et al., 2023;Li et al., 2024), Coptis chinensis Franch. (Li et al., 2024), Acorus tatarinowii Schott (Li et al., 2024), Bupleurum chinense DC. (Li et al., 2024), Panax ginseng C. A. Mey. (Liang and Wang, 2016), Crataegus pinnatifida Bge. (Liang

and Wang, 2016), Alisma orientale（Sam.）Juzep. (Liang and Wang, 2016), Codonopsis pilosula (Franch.)Nannf. (Li et al., 2023)

Reference

Chen, C. (2015). *Observation on the Therapeutic Effect of Zhibai Dihuang Decoction on Type 2 Diabetes Mellitus and Its Impact on HOMA-IR.* Master.

Chen, S., and Wang, Y. (2021). Modified Huanglian Detoxification Decoction combined with metformin in the treatment of 50 cases of diabetes complicated with obesity. *Zhejiang Journal of Traditional Chinese Medicine* 56, 711-712.

Chen, X. (2018). *Clinical Observation on the Treatment of T2DM Combined with Non-alcoholic Fatty Liver Disease with Phlegm-Heat Interlocking Syndrome by Modified Huanglian Wenchan Decoction.* master.

Cheng, M. (2018). *Observation on the Therapeutic Effect of Modified Gegen Qinlian Decoction Combined with Metformin in the Treatment of Type 2 Diabetes with Internal Accumulation of Damp Heat.* master.

Cui, H., and Chen, Y. (2015). Clinical Observation on the Treatment of Type 2 Diabetes with Modified Da-Chai-Hu Decoction. *Hebei Journal of Traditional Chinese Medicine* 37, 1195-1197.

Feng, Z. (2017). Clinical Observation on the Treatment of Type 2 Diabetes Mellitus of the Spleen Deficiency and Phlegm-Stasis Type with Modified Shenling Baizhu Powder Combined with Metformin. *Asia-Pacific Journal of Traditional Medicine* 13, 160-161.

Gong, Y., Shen, Y., Li, K., and Ma, L. (2012). Clinical Observation on the Treatment of Type 2 Diabetes Mellitus of the Spleen Deficiency and Phlegm-Stasis Type with Modified Shenling Baizhu Powder Combined with Metformin. *Xinjiang Journal of Traditional Chinese Medicine* 30, 19-21.

Ji, G. (2017). *Clinical Observation on the Treatment of Abdominal Type Type 2 Diabetes Mellitus with Insulin Resistance by Huanglian Wenchan Decoction.* master.

Ji, J., and Che, Z. (2020). Modified Da Chenghu Decoction combined with metformin tablets for the treatment of 20 cases of type 2 diabetes. *Journal of Traditional Chinese Medicine Research* 33, 18-21.

Li, J., and Feng, S. (2023). The effect of Zhibai Dihuang Decoction combined with Western medicine on blood glucose levels and insulin resistance in patients with type 2 diabetes. *Chinese Contemporary Medical Journal* 30, 48-51.

Li, J., Xiao, Q., and Xiong, L. (2024). Clinical Observation on the Treatment of Prediabetes with Modified Linggui Zhugan Decoction. *Chinese Journal of Clinical Research in Traditional Chinese Medicine* 16, 81-83.

Li, Q., Wu, R., Guo, F., An, R., and Shi, B. (2023). Modified Linggui Zhuanshan Gancao Decoction for Treating Obesity-Type Type 2 Diabetes. *Jilin Journal of Traditional Chinese Medicine* 43, 784-788.

Liang, H., and Wang, S. (2016). Linggui Shugan Decoction + Intervention of Lifestyle Combined with Metformin for the Treatment of Diabetes and Obesity Caused by Excessive Phlegm: A Randomized Parallel Controlled Study. *Practical Journal of Traditional Chinese Internal Medicine* 30, 40-42.

Rong, Y. (2019). A Randomized Parallel Controlled Study on the Treatment of Diabetes (with

Excessive Heat in the Lung and Stomach) by Baihu Jia Ren Shen Decoction. *Practical Journal of Traditional Chinese Internal Medicine* 33, 32-34.

Wang, D. (2022). The clinical efficacy of modified Da-Chai-Hu Decoction combined with metformin tablets in the treatment of type 2 diabetes. *Heilongjiang Journal of Medical Sciences* 45, 26-28.

Wang, L. (2021). Clinical Study on the Treatment of Newly Diagnosed Type 2 Diabetes with Phlegm (Dampness) Heat Interlocking Syndrome by Modified Gegen Qinlian Decoction. *Journal of New Traditional Chinese Medicine* 53, 16-20.

Wu, L. (2021). *Clinical study on the treatment of type 2 diabetes with damp-heat obstruction pattern by modified Gegen Keminlian Decoction.* master.

Xie, F. (2023). Observation on the Effect of Modified Gegen Kinfenlian Decoction in Regulating Blood Glucose Levels in Patients with Type 2 Diabetes. *Journal of Marriage, Reproduction and Health* 29, 112-114.

Yang, X. (2021). *Clinical Research on Modified Ge Gen Qin Lian Decoction Combined with Metformin in the Treatment of Damp-Heat Obstructing the Spleen Syndrome of Type 2 Diabetes Mellitus.* Master.

Yu, H. (2023). A Study on the Clinical Effectiveness and Safety of Gegen Qinlian Decoction in Treating Diabetic Patients. *Smart Health Magazine* 9, 142-146.

Zhang, L. (2017). A Randomized Parallel Controlled Study on the Treatment of Type 2 Diabetes with Severe Heat-Induced Dehydration Using Huanglian Jiedu Decoction Combined with Western Medicine. *Practical Journal of Traditional Chinese Internal Medicine* 31, 34-37.

Zhang, S. (2025). Clinical Observation on the Treatment of Type 2 Diabetes with Modified Gegen Kinfenlian Decoction Combined with Metformin for the Syndrome of Dampness and Heat Accumulating in the Spleen. *Practical Chinese Medicine Journal* 41, 371-373.

Zhou, Y. (2023). *Observation on the Clinical Efficacy of Modified Da-Chai-Hu Decoction in Treating Type 2 Diabetes Mellitus with Metabolic Syndrome Caused by Liver-Gallbladder Stagnation and Blood Stasis.* master.
