# Supplementary material for: Efficacy and safety of traditional Chinese classic prescriptions combined with metformin in the treatment of type 2 diabetes mellitus: a Bayesian network meta-analysis
Source: Front Pharmacol. 2026 Feb 11;17:1693378. doi: 10.3389/fphar.2026.1693378 (PMC12932438; doi:10.3389/fphar.2026.1693378)
Supplement: Supplementary file 4 [file DataSheet4.pdf]

Exclusion studies and reasons (total records 155)	
**Exclusion studies**	**Reasons**
Xu HY, 2016	Non randomized controlled trial
Zhang Y, 2013	Non randomized controlled trial
Deng PP, 2021	Non randomized controlled trial
Liu Y, 2020	Non randomized controlled trial
Yang AH, 2015	Non randomized controlled trial
Zhai RQ, 2023	Outcome measures not applicable
Guan WZ, 2018	Outcome measures not applicable
Guan YL, 2016	Outcome measures not applicable
Guo Q, 2018	Outcome measures not applicable
Jiang F, 2021	Outcome measures not applicable
Kang XL, 2017	Outcome measures not applicable
Liu JD, 2020	Outcome measures not applicable
Liu Y, 2017	Outcome measures not applicable
Shi GF, 2023	Outcome measures not applicable
Tan DN, 2024	Outcome measures not applicable
Wang C, 2021	Outcome measures not applicable
Xie GZ, 2017	Outcome measures not applicable
Zhang LY, 2010	Outcome measures not applicable
Zhao SC, 2023	Outcome measures not applicable
Zheng LX, 2008	Outcome measures not applicable
Guo SS, 2023	Repeat publication
Jiang F, 2021	Repeat publication
Peng SL, 2015	Repeat publication
Yang WJ, 2013	Repeat publication
Cai SN, 2019	Full-text is not available
Dai MJ, 2014	Full-text is not available
He Q, 2019	Full-text is not available
Li NY, 2018	Full-text is not available
Luo L, 2018	Full-text is not available
Wu XZ, 2020	Full-text is not available
Zhang GG, 2006	Full-text is not available
Zhao HB, 2021	Full-text is not available
Cai BS, 2020	No baseline assessment data
Deng M, 2015	No baseline assessment data
Gao HY, 2020	No baseline assessment data
Wang LQ, 2020	No baseline assessment data
Xiu H, 2020	No baseline assessment data
Zhang XT, 2014	No baseline assessment data
Deng B, 2016	No baseline assessment data
Bian ZZ, 2024	Non TCM combined with Met
Cao F, 2021	Non TCM combined with Met

Cao R, 2018	Non TCM combined with Met
Chang DY, 2020	Non TCM combined with Met
Shen KL, 2014	Non TCM combined with Met
Chen Q, 2020	Non TCM combined with Met
Chen XH, 2012	Non TCM combined with Met
Chen YF, 2024	Non TCM combined with Met
Chen ZN, 2010	Non TCM combined with Met
Cheng H, 2008	Non TCM combined with Met
Cui Y, 2019	Non TCM combined with Met
Du JH, 2022	Non TCM combined with Met
Du M, 2023	Non TCM combined with Met
Du TC, 2021	Non TCM combined with Met
Fan XF, 2017	Non TCM combined with Met
Feng L, 2017	Non TCM combined with Met
Feng W, 2019	Non TCM combined with Met
Feng YL, 2016	Non TCM combined with Met
Fu NY, 2012	Non TCM combined with Met
Fu YH, 2016	Non TCM combined with Met
Fu JB, 2012	Non TCM combined with Met
Geng LP, 2015	Non TCM combined with Met
Gong YL, 2018	Non TCM combined with Met
Guo J, 2019	Non TCM combined with Met
Guo TT, 2019	Non TCM combined with Met
Han JH, 2020	Non TCM combined with Met
Han WL, 2023	Non TCM combined with Met
He K, 2016	Non TCM combined with Met
Huang HL, 2018	Non TCM combined with Met
Huang WZ, 2022	Non TCM combined with Met
Huang ZY, 2010	Non TCM combined with Met
Ji WJ, 2013	Non TCM combined with Met
Jin LL, 2016	Non TCM combined with Met
Kang XD, 2019	Non TCM combined with Met
Li FF, 2019	Non TCM combined with Met
Li HH, 2008	Non TCM combined with Met
Li L, 2020	Non TCM combined with Met
Li LP, 2018	Non TCM combined with Met
Li SY, 2024	Non TCM combined with Met
Li Z, 2023	Non TCM combined with Met
Lian XM, 2017	Non TCM combined with Met
Lin GL, 2024	Non TCM combined with Met
Liu JH, 2024	Non TCM combined with Met
Liu LZ, 2008	Non TCM combined with Met
Liu XD, 2006	Non TCM combined with Met
Liu Y, 2019	Non TCM combined with Met

Liu YT, 2023	Non TCM combined with Met
Lu M, 2015	Non TCM combined with Met
Lu XF, 2025	Non TCM combined with Met
Meng JJ, 2022	Non TCM combined with Met
Mo HB, 2018	Non TCM combined with Met
Mu NR, 2021	Non TCM combined with Met
Nian JY, 2023	Non TCM combined with Met
Ou L, 2021	Non TCM combined with Met
Qi XL, 2012	Non TCM combined with Met
Qin CY, 2015	Non TCM combined with Met
Qu DL, 2016	Non TCM combined with Met
Liu LZ, 2008	Non TCM combined with Met
Rong WW, 2024	Non TCM combined with Met
Ruan HZ, 2022	Non TCM combined with Met
Shi AM, 2022	Non TCM combined with Met
Shi JB, 2017	Non TCM combined with Met
Shi CP, 2018	Non TCM combined with Met
Shi JY, 2019	Non TCM combined with Met
Song QJ, 2023	Non TCM combined with Met
Tan QL, 2019	Non TCM combined with Met
Tang Y, 2015	Non TCM combined with Met
Tang YQ, 2023	Non TCM combined with Met
Tu CL, 2015	Non TCM combined with Met
Wan Y, 2019	Non TCM combined with Met
Wang FJ, 2022	Non TCM combined with Met
Wang XQ, 2017	Non TCM combined with Met
Wang CF, 2018	Non TCM combined with Met
Wang GC, 2025	Non TCM combined with Met
Wang H, 2016	Non TCM combined with Met
Wang H, 2015	Non TCM combined with Met
Wang JJ, 2016	Non TCM combined with Met
Wang Q, 2022	Non TCM combined with Met
Wang XP, 2008	Non TCM combined with Met
Wang YH, 2014	Non TCM combined with Met
Wang YS, 2024	Non TCM combined with Met
Wang Y, 2024	Non TCM combined with Met
Wei XL, 2010	Non TCM combined with Met
Wu HZ, 2013	Non TCM combined with Met
Wu JL, 2005	Non TCM combined with Met
Wu JX, 2021	Non TCM combined with Met
Wu M, 2024	Non TCM combined with Met
Wu Y, 2011	Non TCM combined with Met
Xia ZL, 2011	Non TCM combined with Met
Xie J, 2014	Non TCM combined with Met

Xing Y, 2017	Non TCM combined with Met
Xiu Y, 2022	Non TCM combined with Met
Xu HJ, 2019	Non TCM combined with Met
Xu NJ, 2008	Non TCM combined with Met
Xu YW, 2015	Non TCM combined with Met
Yang JY, 2014	Non TCM combined with Met
Yang X, 2020	Non TCM combined with Met
Yin HS, 2018	Non TCM combined with Met
Ying YL, 2019	Non TCM combined with Met
Zhang GP, 2015	Non TCM combined with Met
Zhang HY, 2013	Non TCM combined with Met
Zhang LP, 2023	Non TCM combined with Met
Zhang LM, 2014	Non TCM combined with Met
Zhang W, 2017	Non TCM combined with Met
Zhang XY, 2005	Non TCM combined with Met
Zhang Z, 2023	Non TCM combined with Met
Zhang ZN, 2020	Non TCM combined with Met
Zhao HR, 2015	Non TCM combined with Met
Zhao J, 2024	Non TCM combined with Met
Zhao M, 2016	Non TCM combined with Met
Zhao YN, 2016	Non TCM combined with Met
Zhao ZY, 2006	Non TCM combined with Met
Zheng HX, 2014	Non TCM combined with Met
Zheng LX, 2017	Non TCM combined with Met
Zheng SM, 2018	Non TCM combined with Met
Zhu YG, 2018	Non TCM combined with Met
