# Supplementary material for: Efficacy and safety of traditional Chinese classic prescriptions combined with metformin in the treatment of type 2 diabetes mellitus: a Bayesian network meta-analysis
Source: Front Pharmacol. 2026 Feb 11;17:1693378. doi: 10.3389/fphar.2026.1693378 (PMC12932438; doi:10.3389/fphar.2026.1693378)
Supplement: Supplementary file 5 [file DataSheet6.pdf]

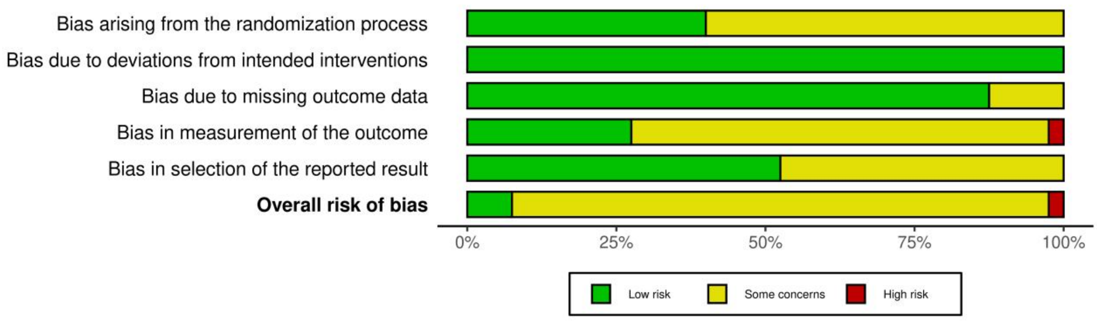

Bias arising from the randomization process
Bias due to deviations from intended interventions
Bias due to missing outcome data
Bias in measurement of the outcome
Bias in selection of the reported result
Overall risk of bias
0%
25%
50%
75%
100%
Low risk
Some concerns
High risk

Risk of bias domains

Study	D1	D2	D3	D4	D5	Overall
Shaowu Zeng 2022	-	+	+	-	+	-
Chunyuan Chen 2015	+	+	+	X	+	X
Sisi Chen 2021	-	+	+	-	-	-
Xia Chen 2018	-	+	+	-	-	-
Ye Chen 2022	-	+	+	-	-	-
Mengjie Cheng 2022	-	+	+	-	-	-
Hongyan Cui 2015	-	+	+	-	-	-
Guoling Dai 2022	-	+	+	-	-	-
Jiajun Feng 2019	+	+	+	+	-	-
Zhanrong Feng 2020	+	+	+	-	-	-
Zhaolan Feng 2017	-	+	+	-	-	-
Guizhen Fu 2017	+	+	+	-	-	-
Ying Gong 2012	-	+	+	-	-	-
Shangshang Pan 2021	-	+	+	-	-	-
Guanghui Ji 2017	-	+	+	-	+	-
Juliang Ji 2020	+	+	+	-	+	-
Hua Li 2018	+	+	-	-	+	-
Jinhua Li 2024	-	+	-	-	+	-
Juanjuan Li 2023	+	+	-	-	+	-
Houce Liang 2016	-	+	+	-	+	-
Qin Li 2023	+	+	-	-	+	-
Xuelin Luo 2005	-	+	+	-	+	-
Linna Ma 2022	-	+	+	-	+	-
Shaolin Peng 2015	-	+	+	-	+	-
Yanhong Rong 2019	+	+	+	+	+	+
Qin Tan 2017	+	+	+	+	+	+
Qin Tan 2021	+	+	+	+	+	+
Dongmei Wang 2022	-	+	+	+	-	-
Lei Wang 2021	+	+	+	+	-	-
Mingkun Wang 2021	+	+	+	+	-	-
Yan Wang 2022	-	+	+	-	-	-
Li Wu 2021	-	+	+	+	-	-
Fangyi Xie 2023	-	+	+	+	-	-
Wenjun Yang 2013	-	+	+	-	-	-
Xueqin Yang 2021	-	+	-	+	+	-
Huiling Yu 2023	-	+	+	+	+	-
Hongguo Yuan 2023	+	+	+	-	+	-
Lili Zhang 2017	+	+	+	-	+	-
Siying Zhang 2025	-	+	+	-	+	-
Yuting Zhou 2023	+	+	+	-	+	-

Domains:
D1: Bias arising from the randomization process.
D2: Bias due to deviations from intended intervention.
D3: Bias due to missing outcome data.
D4: Bias in measurement of the outcome.
D5: Bias in selection of the reported result.

Judgement
X High
- Some concerns
+ Low
