# Supplementary material for: Efficacy and safety of traditional Chinese classic prescriptions combined with metformin in the treatment of type 2 diabetes mellitus: a Bayesian network meta-analysis
Source: Front Pharmacol. 2026 Feb 11;17:1693378. doi: 10.3389/fphar.2026.1693378 (PMC12932438; doi:10.3389/fphar.2026.1693378)
Supplement: Supplementary file 6 [file DataSheet9.pdf]

Investigation into Network Inconsistency for Lipid Profiles (TC and TG)

1. Overview of the Inconsistency Investigation

As the evidence network for Total Cholesterol (TC) and Triglycerides (TG) follows a predominantly star-shaped architecture (where all botanical formulas are compared only to Metformin with no head-to-head trials), the global inconsistency detected by the model does not stem from "loop inconsistency" (as no closed loops exist). Instead, it primarily reflects significant within-node heterogeneity across the primary studies. To explore the sources of this inconsistency, study-level forest plots were generated to visualize the effect size distribution for each treatment node.

2. Analysis of Total Cholesterol (TC) Heterogeneity

Figure S1 illustrates the study-level Mean Differences (MD) for TC. The analysis identifies marked discrepancies within specific intervention nodes:

SLBZD + Met: There is a substantial divide in reported efficacy. Studies by Shaowu Zeng and Hongguo Yuan reported significant TC reduction (MD approximately -0.7 to -0.9), whereas studies by Ying Gong and Zhaolan Feng showed near-null effects (MD approx. -0.1).

LGZGD + Met: A significant gap exists between the results of Qin Li (MD = -1.51) and Jinhua Li (MD = -0.28). These clinical variations in reported outcomes contribute to the instability of the global inconsistency test for TC.

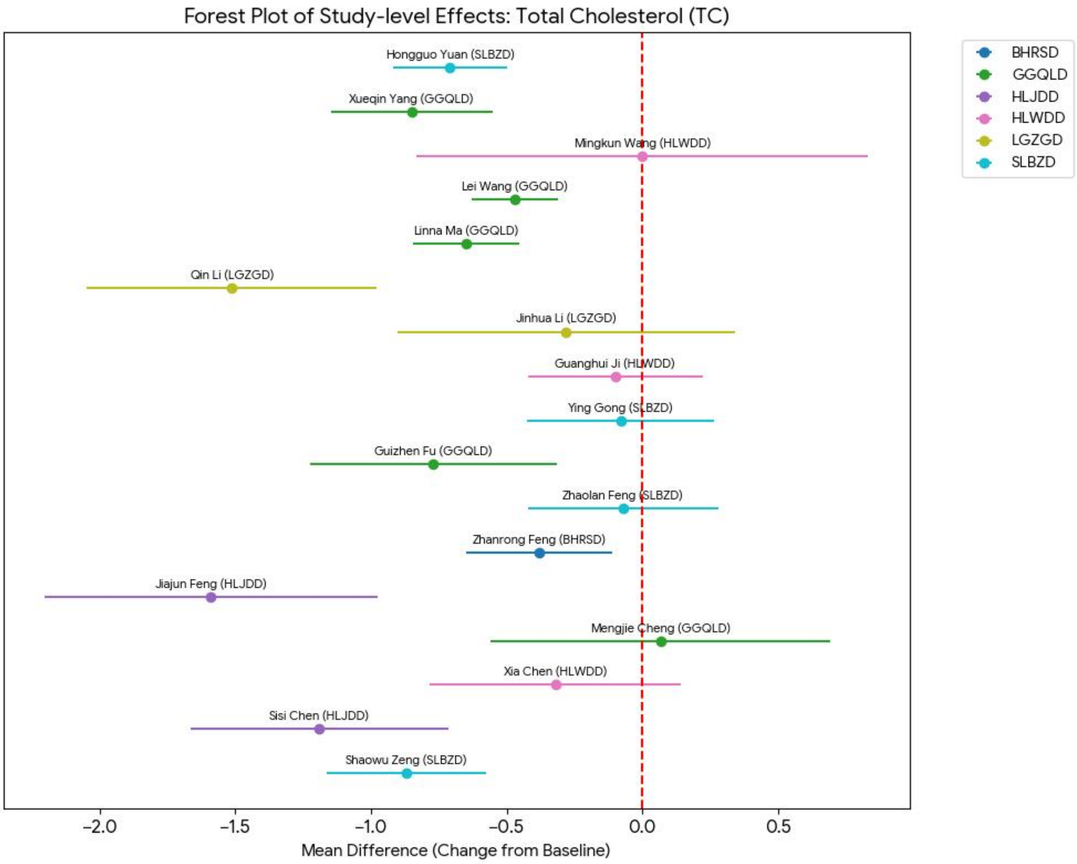

Figure S1Forest Plot of Study-level Effects: TC

3. Analysis of Triglycerides (TG) Heterogeneity

Figure S2 displays the study-level MD for TG. The inconsistency in this network is largely driven by contradictory findings within the LGZGD + Met comparison:

A major conflict is observed between the study by Qin Li (which showed a strong reduction in TG, MD = -1.75) and the study by Jinhua Li (which reported a slight increase in TG, MD = +0.11). Such extreme outliers in small-sample trials disrupt the transitivity across the lipid network, leading to the identified global inconsistency.

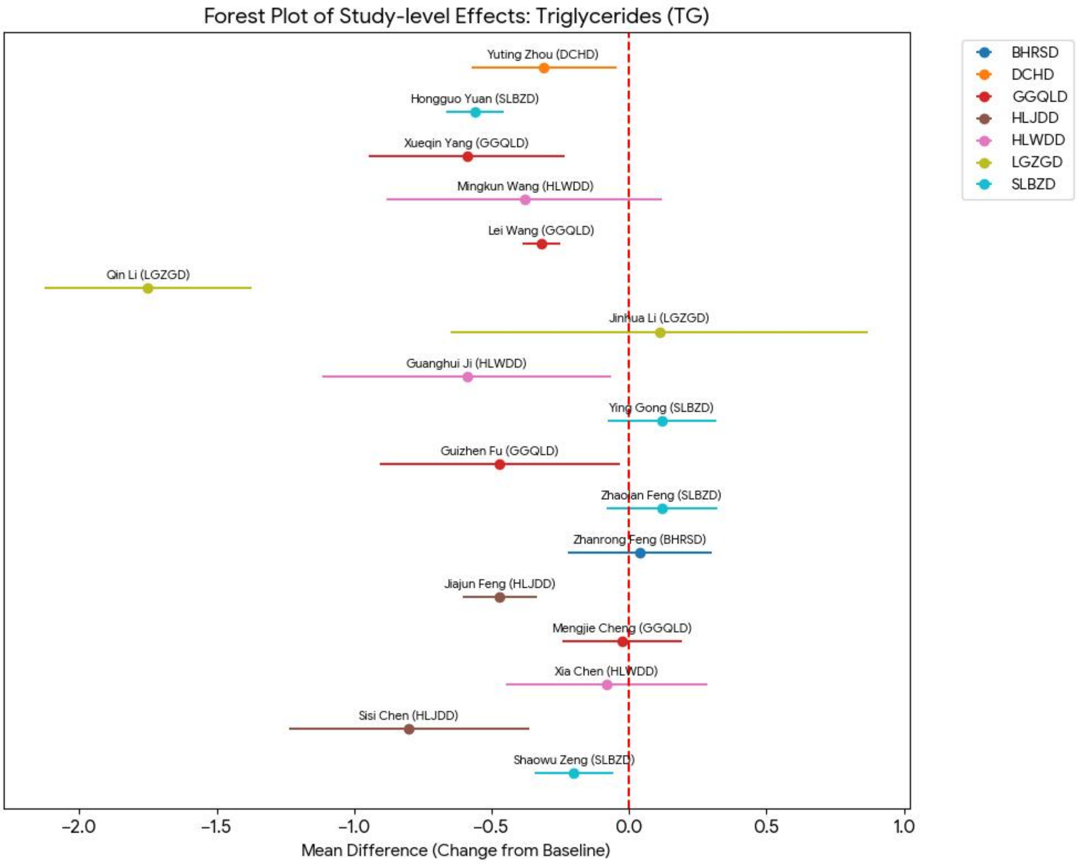

Figure S2 Forest Plot of Study-level Effects: TG

The visual evidence from the study-level forest plots confirms that the statistical inconsistency is a product of high clinical heterogeneity rather than structural model failure. Factors such as baseline lipid levels, patient diet, and trial duration likely contribute to these divergent results. Consequently, the relative rankings for TC and TG should be considered exploratory and interpreted with caution.
