# Supplementary material for: Efficacy and safety of traditional Chinese classic prescriptions combined with metformin in the treatment of type 2 diabetes mellitus: a Bayesian network meta-analysis
Source: Front Pharmacol. 2026 Feb 11;17:1693378. doi: 10.3389/fphar.2026.1693378 (PMC12932438; doi:10.3389/fphar.2026.1693378)
Supplement: Supplementary file 7 [file DataSheet11.pdf]

Summary of Meta-regression Analysis for All Outcomes.

Outcome	No. of Studies	Significant Covariate	Coefficient	P-value	Interpretation
HbA1c	33	Intervention Duration	-0.701 (-1.32 to -0.08)	0.028	Longer duration to Better efficacy
FPG	40	Intervention Duration	-0.549 (-1.01 to -0.09)	0.022	Longer duration to Better efficacy
2hPG	38	Intervention Duration	-0.474 (-0.96 to 0.01)	0.053	No significant predictor identified
FINS	15	Intervention Duration	-1.757 (-3.07 to -0.45)	0.013	Longer duration to Better efficacy
HOMA-IR	17	Sample Size	14.018 (10.30 to 17.74)	<0.001	Small-study effects present
TC	17	None	-	>0.05	No significant predictor identified
TG	17	Intervention Duration	0.252 (-0.01 to 0.52)	0.060	No significant predictor identified
LDL-c	15	None	-	>0.05	No significant predictor identified
HDL-c	10	None	-	>0.05	No significant predictor identified

Meta-regression Analysis for FPG

Covariate	Coeff	SE	P-value	95% CI	Whether significant
Intervention time	0.549	0.227	0.022	0.086 to 1.011	Yes
Risk of bias	0.703	0.800	0.386	-0.925 to 2.332	No
Sample size	-0.898	0.603	0.146	-2.124 to 0.329	No
Improved herbal	-0.010	0.225	0.965	-0.467 to 0.448	No

Meta-regression Analysis for 2hPG

Covariate	Coeff	SE	P-value	95% CI	Whether significant
Intervention time	-0.474	0.236	0.053	-0.956 to 0.007	No
Risk of bias	-0.178	1.497	0.906	-3.231 to 2.876	No
Sample size	-0.673	0.472	0.164	-1.636 to 0.290	No
Improved herbal	-0.140	0.228	0.543	-0.605 to 0.324	No

Meta-regression Analysis for HbA1c

Covariate	Coeff	SE	P-value	95% CI	Whether significant
Intervention time	-0.701	0.303	0.028	-1.322 to -0.080	Yes
Risk of bias	0.197	1.138	0.864	-2.133 to 2.527	No
Sample size	-0.231	0.661	0.729	-1.586 to 1.124	No
Improved herbal	-0.280	0.326	0.398	-0.948 to 0.388	No

Meta-regression Analysis for TC

Covariate	Coeff	SE	P-value	95% CI	Whether significant
Risk of bias	-	-	-	-	
Intervention time	0.046	0.183	0.805	-0.349 to 0.440	No
Sample size	-0.056	0.283	0.847	-0.668 to 0.556	No
Improved herbal	-0.312	0.186	0.117	-0.714 to 0.090	No

Meta-regression Analysis for TG

Covariate	Coeff	SE	P-value	95% CI	Whether significant
Risk of bias	-	-	-	-	
Sample size	-	-	-	-	
Intervention time	0.252	0.123	0.060	-0.012 to 0.515	No
Improved herbal	-0.190	0.115	0.122	-0.437 to 0.057	No

Meta-regression Analysis for LDL-c

Covariate	Coeff	SE	P-value	95% CI	Whether significant
Risk of bias	-	-	-	-	
Intervention time	-0.044	0.458	0.925	-1.052 to 0.964	No
Sample size	-0.049	0.518	0.926	-1.190 to 1.092	No
Improved herbal	0.035	0.448	0.939	-0.952 to 1.022	No

Meta-regression Analysis for HDL-c

Covariate	Coeff	SE	P-value	95% CI	Whether significant
Risk of bias	-	-	-	-	
Sample size	-	-	-	-	
Intervention time	0.295	0.169	0.124	-0.104 to 0.693	No
Improved herbal	0.262	0.164	0.153	-0.125 to 0.649	No

Meta-regression Analysis for HOMA-IR

Covariate	Coeff	SE	P-value	95% CI	Whether significant
Sample size	14.018	1.722	< 0.001	10.297 to 17.739	Yes
Intervention time	0.063	0.268	0.818	-0.516 to 0.642	No
Improved herbal	-0.181	0.246	0.475	-0.713 to 0.351	No
Risk of bias	-	-	-	-	

Meta-regression Analysis for FINS

Covariate	Coeff	SE	P-value	95% CI	Whether significant
Intervention time	-1.757	0.601	0.013	-3.067 to -0.448	Yes
Improved herbal	0.887	0.629	0.184	-0.484 to 2.258	No
Risk of bias	-	-	-	-	
Sample size	-	-	-	-	
