# Supplementary figures and images for: Efficacy and safety of traditional Chinese classic prescriptions combined with metformin in the treatment of type 2 diabetes mellitus: a Bayesian network meta-analysis
Source: Front Pharmacol. 2026 Feb 11;17:1693378. doi: 10.3389/fphar.2026.1693378 (PMC12932438; doi:10.3389/fphar.2026.1693378)

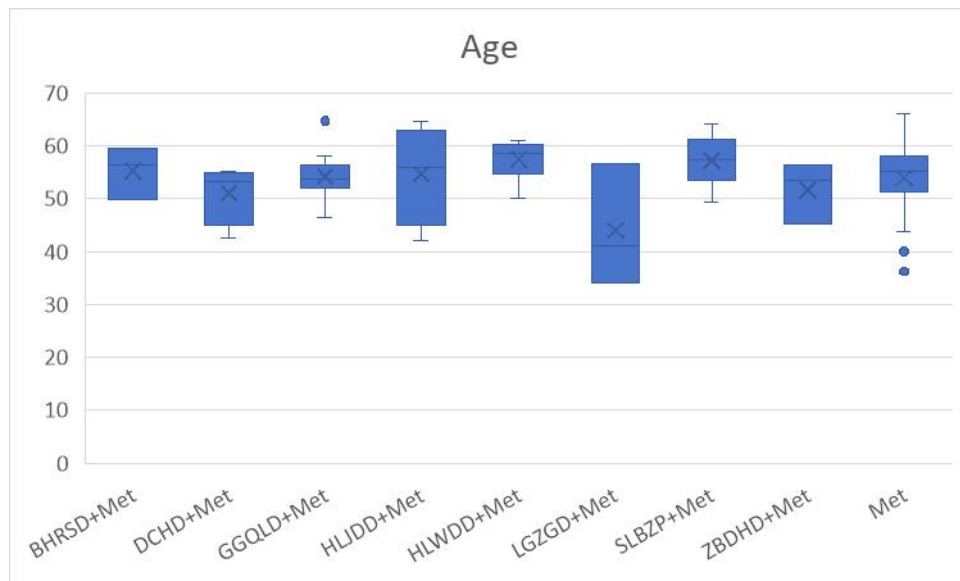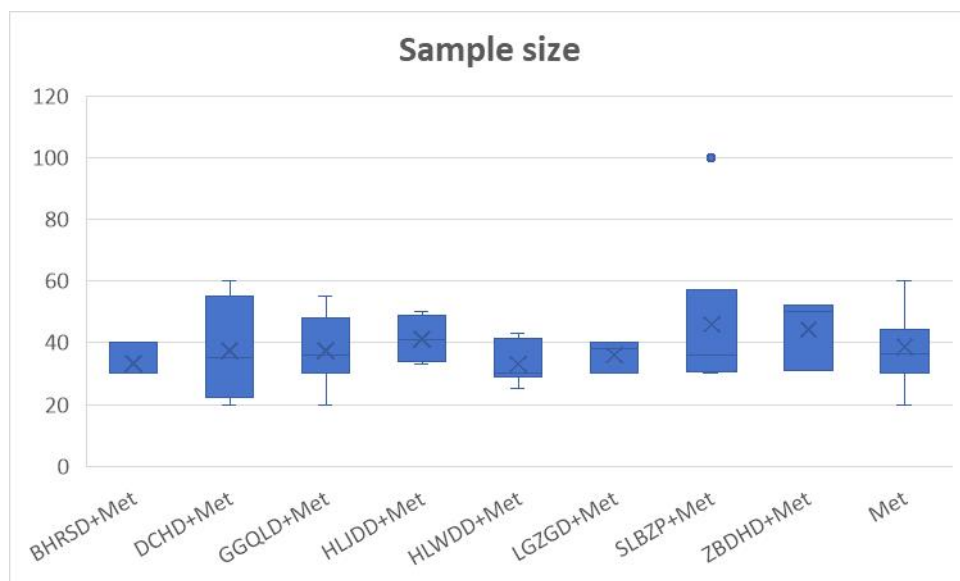

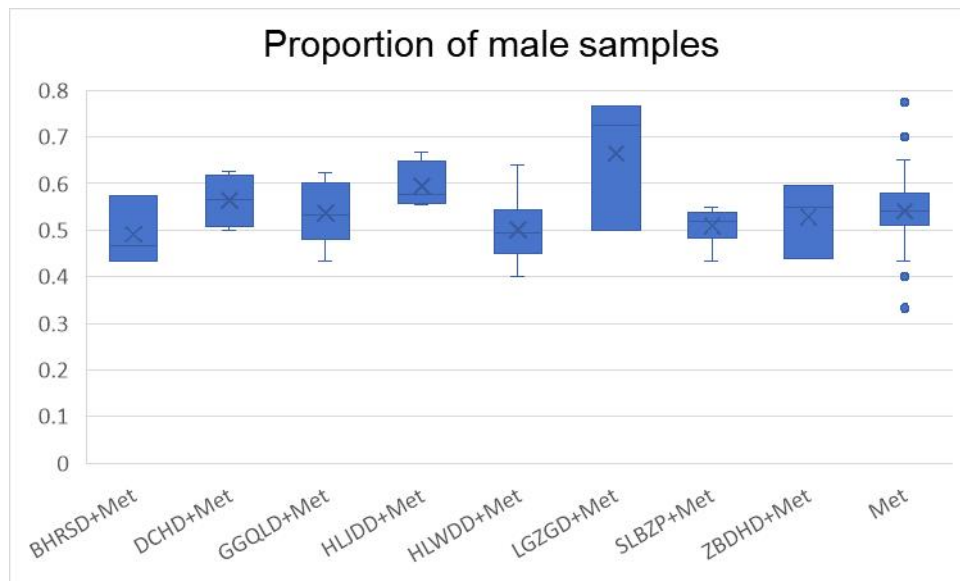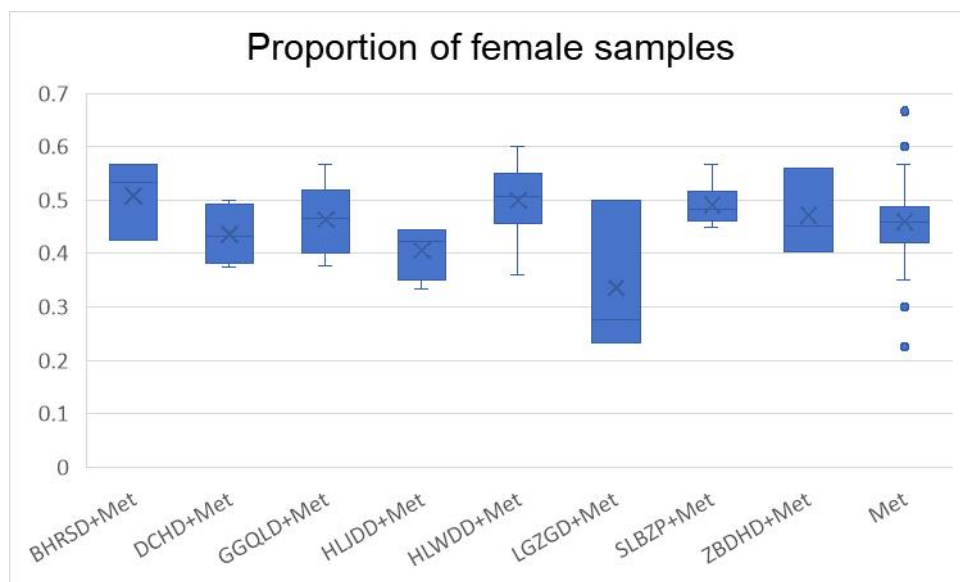

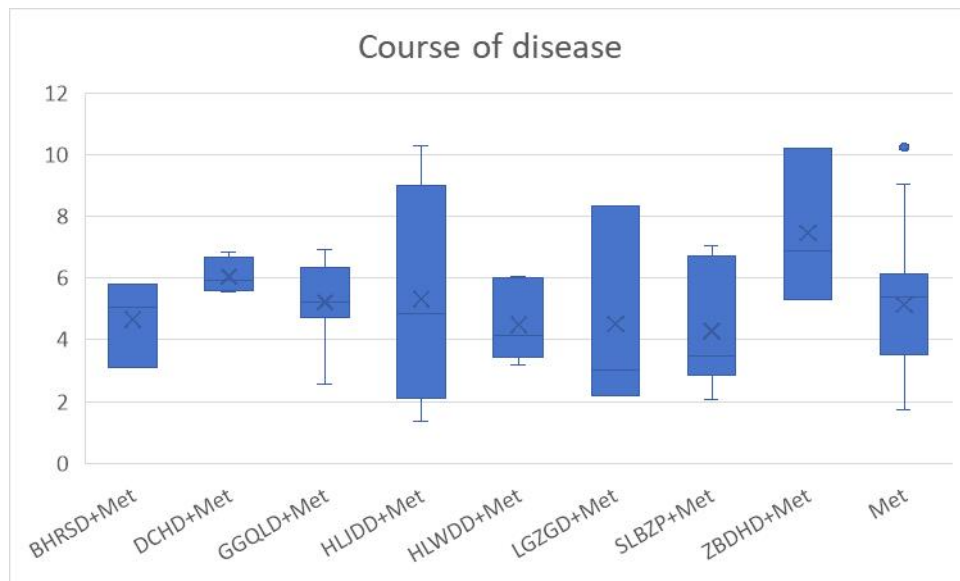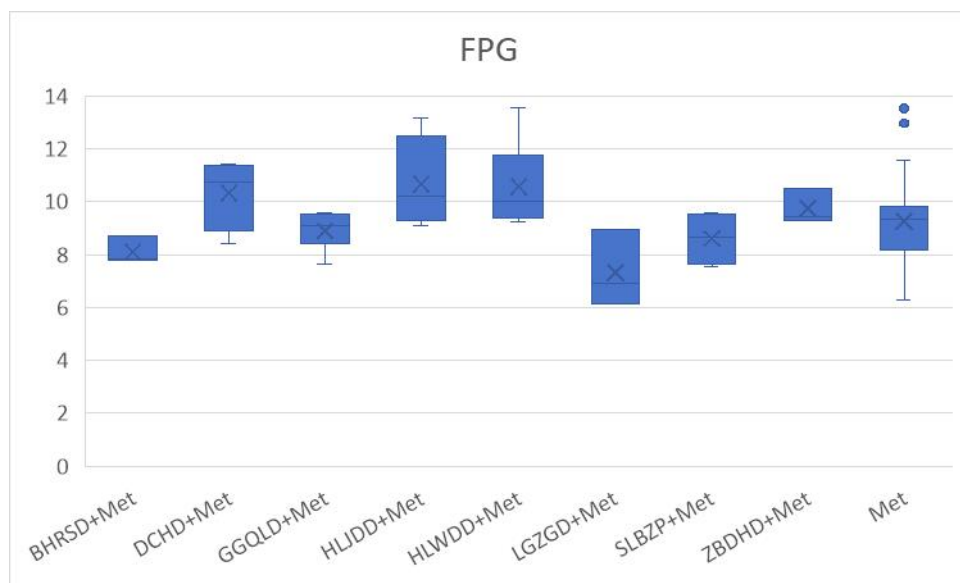

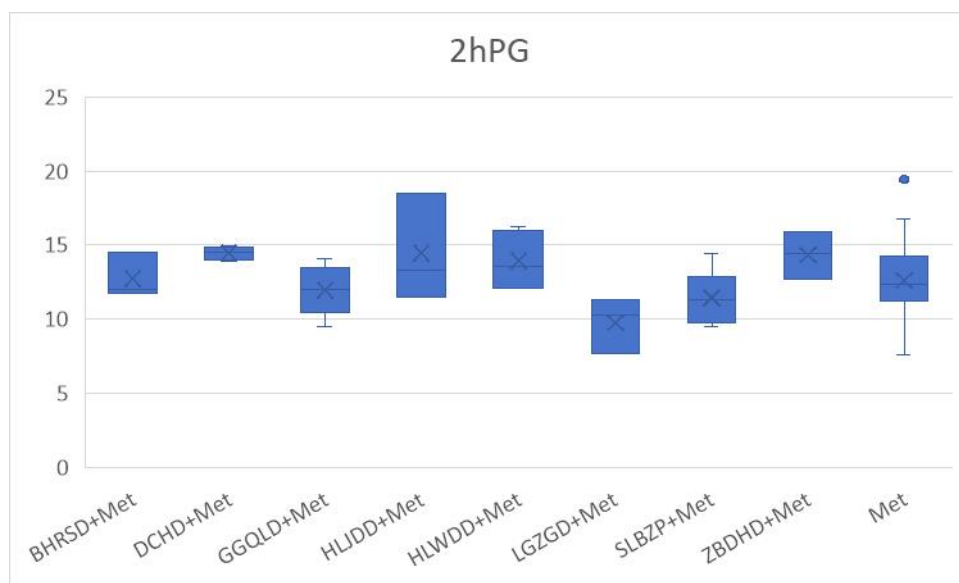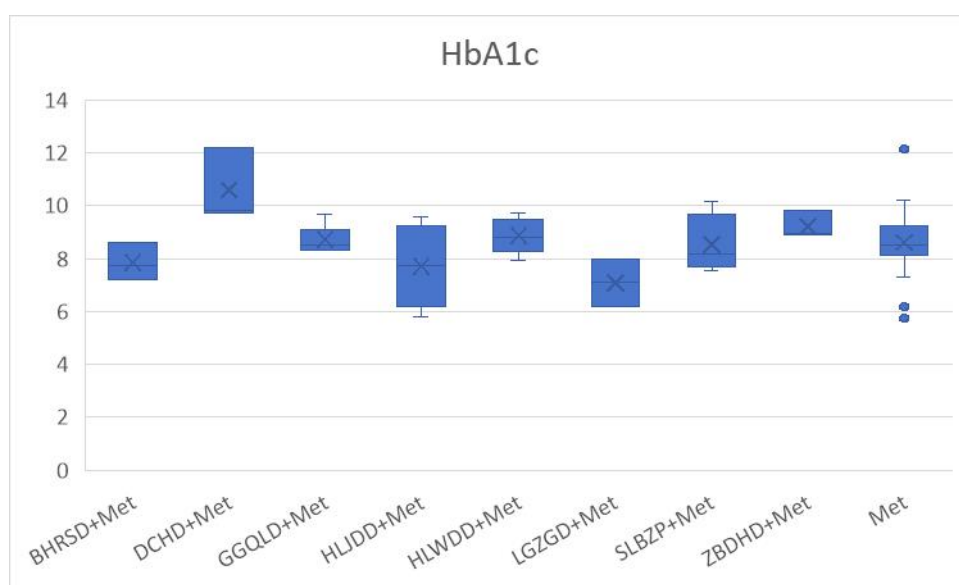

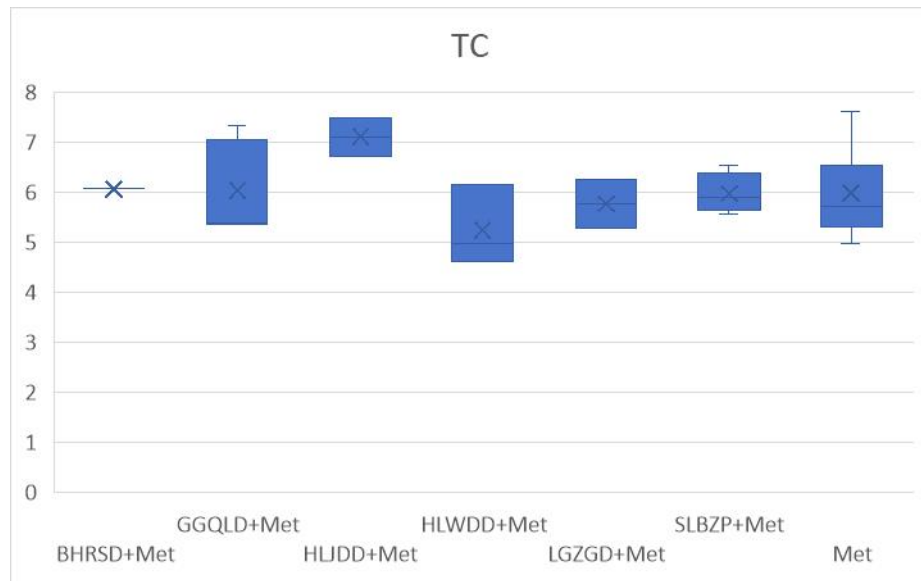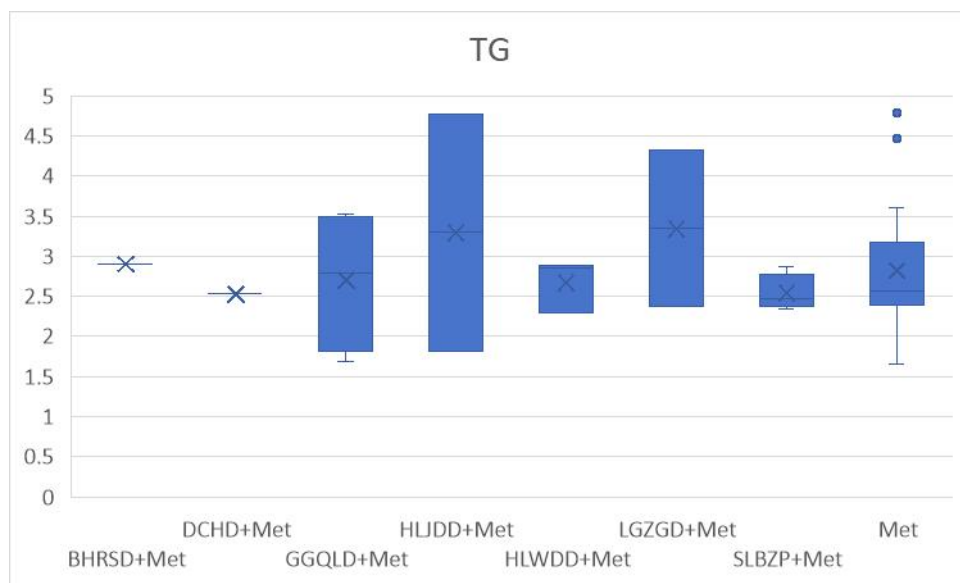

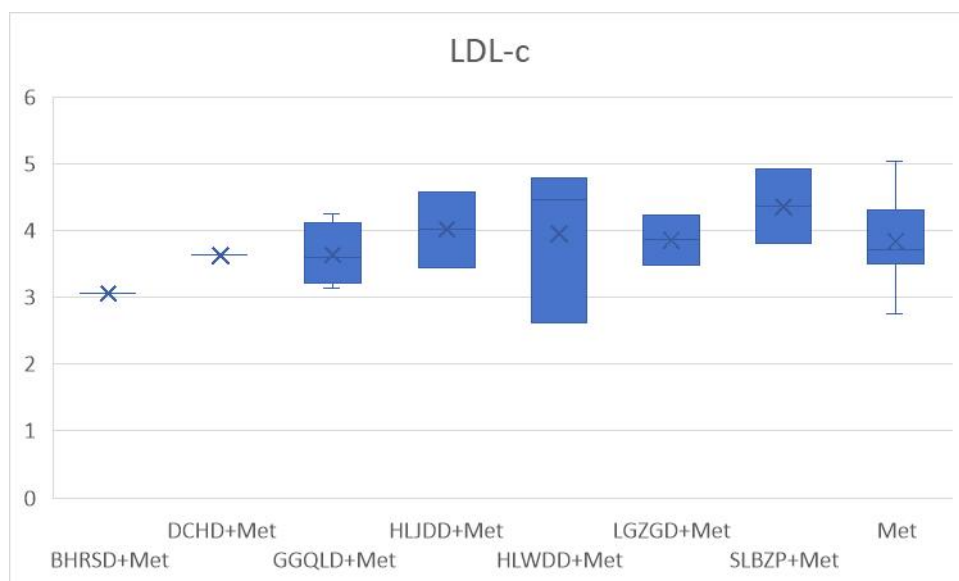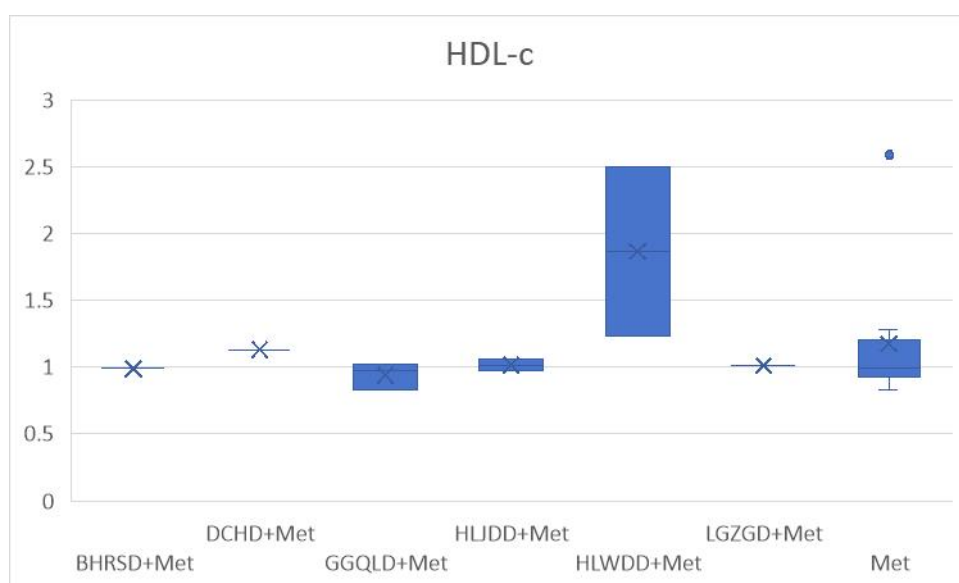

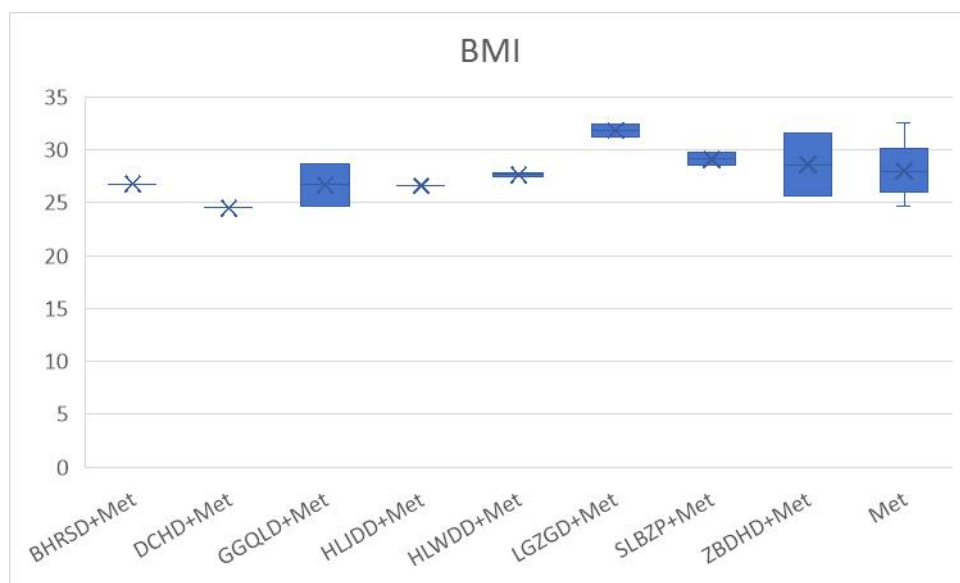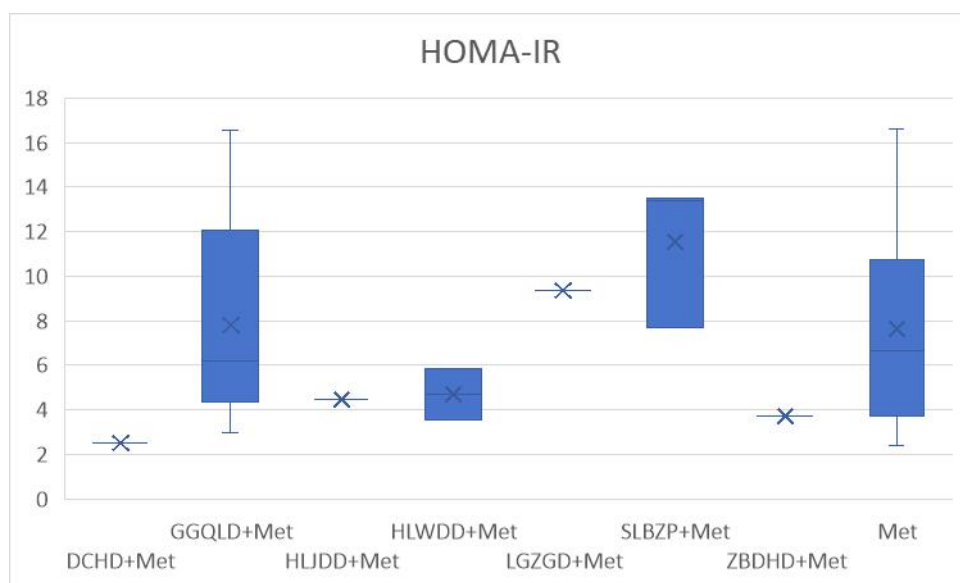

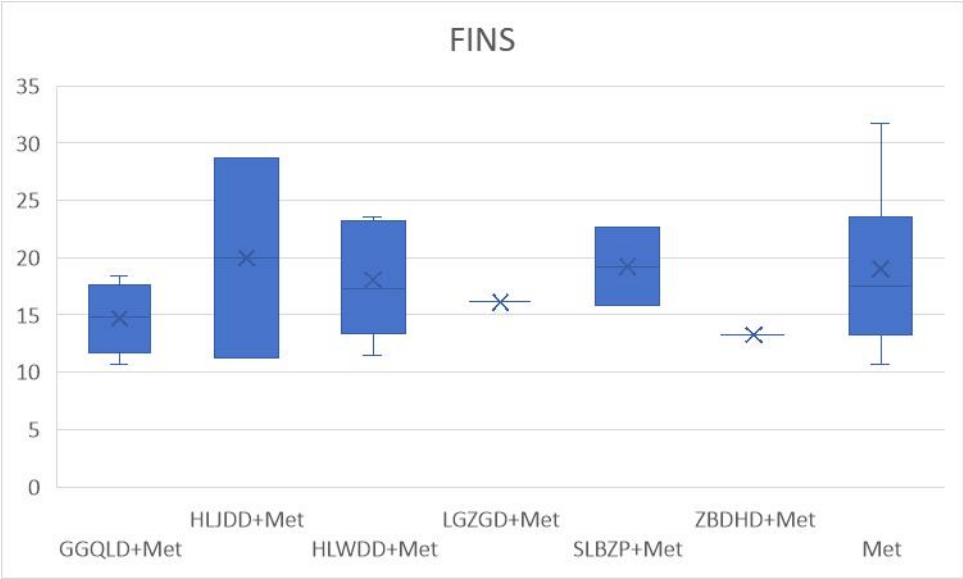

Supplement: Supplementary file 8 [file DataSheet3.pdf]
