# Supplementary material for: Efficacy and safety of traditional Chinese classic prescriptions combined with metformin in the treatment of type 2 diabetes mellitus: a Bayesian network meta-analysis
Source: Front Pharmacol. 2026 Feb 11;17:1693378. doi: 10.3389/fphar.2026.1693378 (PMC12932438; doi:10.3389/fphar.2026.1693378)
Supplement: Supplementary file 9 [file DataSheet1.pdf]

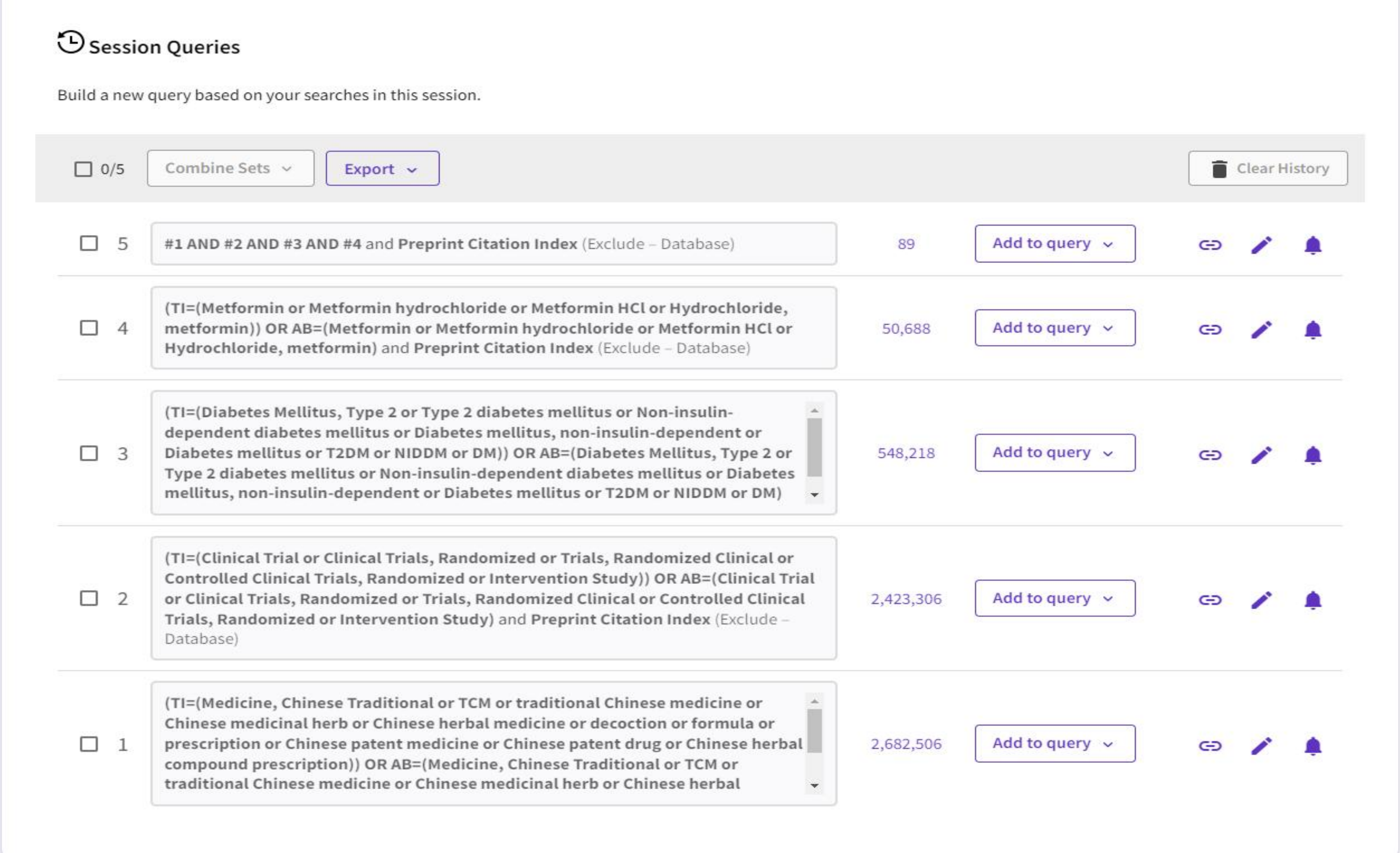

The search strategy of Web of science

The search strategy of Web of science

Entitlements	#		Database	Results
- WOS: 1985 to 2025<br>- CSCD: 1989 to 2025<br>- DIIDW: 1966 to 2025<br>- GRANTS: 1953 to 2025<br>- KJD: 1980 to 2025<br>- MEDLINE: 1950 to 2025<br>- PCI: 1950 to 2025<br>- PPRN: 1991 to 2025<br>- PQDT: 1637 to 2025<br>- SCIELO: 2002 to 2025	1	(TI=(Medicine, Chinese Traditional or TCM or traditional Chinese medicine or Chinese medicinal herb or Chinese herbal medicine or decoction or formula or prescription or Chinese patent medicine or Chinese patent drug or Chinese herbal compound prescription)) OR AB=(Medicine, Chinese Traditional or TCM or traditional Chinese medicine or Chinese medicinal herb or Chinese herbal medicine or decoction or formula or prescription or Chinese patent medicine or Chinese patent drug or Chinese herbal compound prescription) and Preprint Citation Index (Exclude – Database)	All Databases	2682506
- WOS: 1985 to 2025<br>- CSCD: 1989 to 2025<br>- DIIDW: 1966 to 2025<br>- GRANTS: 1953 to 2025<br>- KJD: 1980 to 2025<br>- MEDLINE: 1950 to 2025<br>- PCI: 1950 to 2025<br>- PPRN: 1991 to 2025<br>- PQDT: 1637 to 2025<br>- SCIELO: 2002 to 2025	2	(TI=(Clinical Trial or Clinical Trials, Randomized or Trials, Randomized Clinical or Controlled Clinical Trials, Randomized or Intervention Study)) OR AB=(Clinical Trial or Clinical Trials, Randomized or Trials, Randomized Clinical or Controlled Clinical Trials, Randomized or Intervention Study) and Preprint Citation Index (Exclude – Database)	All Databases	2423306
- WOS: 1985 to 2025<br>- CSCD: 1989 to 2025<br>- DIIDW: 1966 to 2025<br>- GRANTS: 1953 to 2025	3	(TI=(Diabetes Mellitus, Type 2 or Type 2 diabetes mellitus or Non-insulin-dependent diabetes mellitus or Diabetes mellitus, non-insulin-dependent or Diabetes mellitus or T2DM or NIDDM or DM)) OR AB=(Diabetes Mellitus, Type 2 or Type 2 diabetes mellitus or Non-insulin-dependent diabetes mellitus or Diabetes mellitus, non-insulin-dependent or Diabetes mellitus or T2DM or NIDDM or DM) and Preprint Citation Index (Exclude –	All Databases	548218

- KJD: 1980 to 2025<br>- MEDLINE: 1950 to 2025<br>- PCI: 1950 to 2025<br>- PPRN: 1991 to 2025<br>- PQDT: 1637 to 2025<br>- SCIELO: 2002 to 2025		Database)		
- WOS: 1985 to 2025<br>- CSCD: 1989 to 2025<br>- DIIDW: 1966 to 2025<br>- GRANTS: 1953 to 2025<br>- KJD: 1980 to 2025<br>- MEDLINE: 1950 to 2025<br>- PCI: 1950 to 2025<br>- PPRN: 1991 to 2025<br>- PQDT: 1637 to 2025<br>- SCIELO: 2002 to 2025	4	(TI=(Metformin or Metformin hydrochloride or Metformin HCl or Hydrochloride, metformin)) OR AB=(Metformin or Metformin hydrochloride or Metformin HCl or Hydrochloride, metformin) and Preprint Citation Index (Exclude – Database)	All Databases	50688
- WOS: 1985 to 2025<br>- CSCD: 1989 to 2025<br>- DIIDW: 1966 to 2025<br>- GRANTS: 1953 to 2025<br>- KJD: 1980 to 2025<br>- MEDLINE: 1950 to 2025<br>- PCI: 1950 to 2025<br>- PPRN: 1991 to 2025<br>- PQDT: 1637 to 2025<br>- SCIELO: 2002 to 2025	5	#1 AND #2 AND #3 AND #4 and Preprint Citation Index (Exclude – Database)	All Databases	89

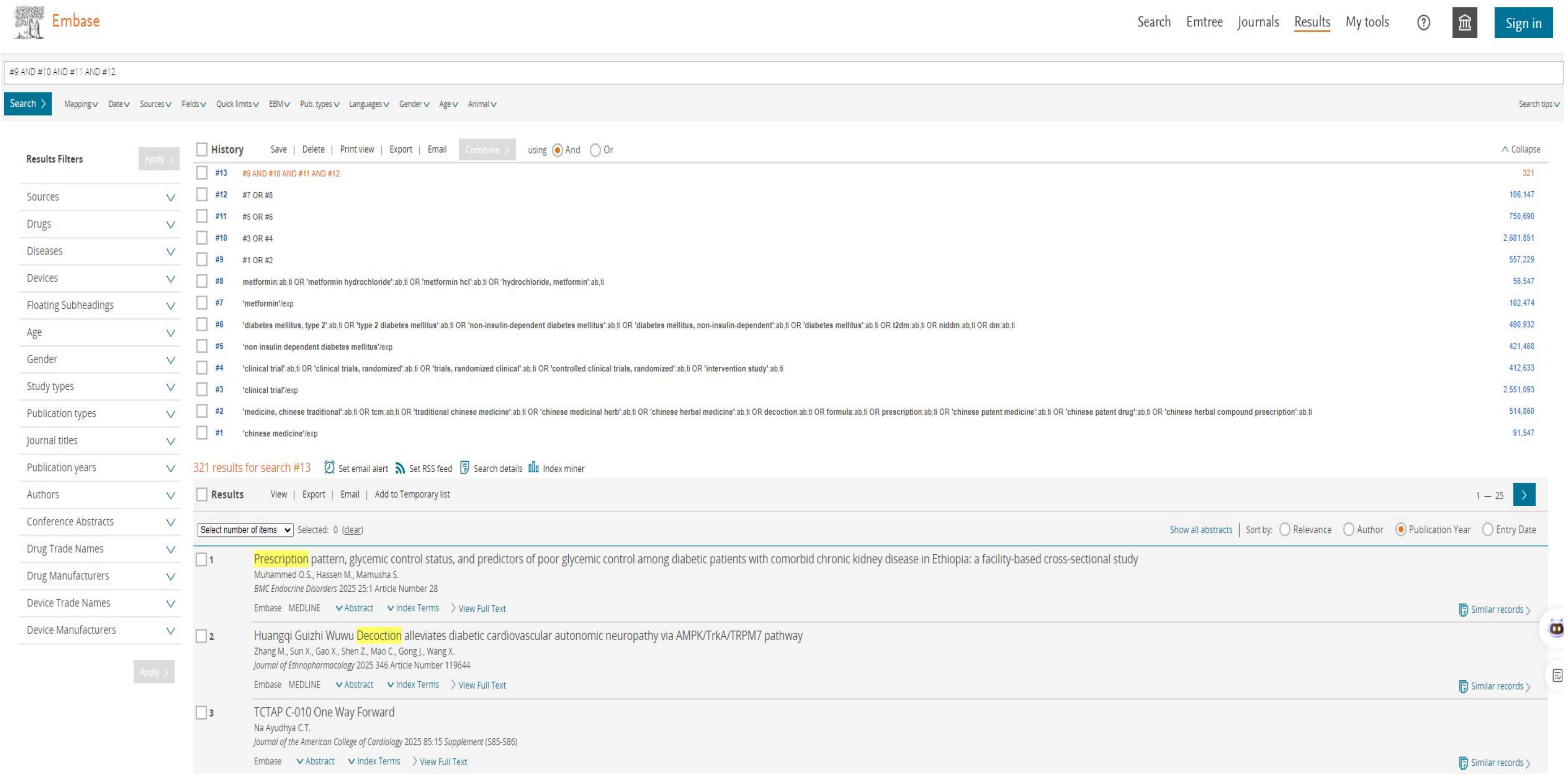

The search strategy of Embase

The search strategy of Embase

No.	Query	Results	Date
#13	#9 AND #10 AND #11 AND #12	321	7-Jun-25
#12	#7 OR #8	106147	7-Jun-25
#11	#5 OR #6	750690	7-Jun-25
#10	#3 OR #4	2681851	7-Jun-25
#9	#1 OR #2	557229	7-Jun-25
#8	metformin:ab,ti OR 'metformin hydrochloride':ab,ti OR 'metformin hcl':ab,ti OR 'hydrochloride, metformin':ab,ti	56547	7-Jun-25
#7	'metformin'/exp	102474	7-Jun-25
#6	'diabetes mellitus, type 2':ab,ti OR 'type 2 diabetes mellitus':ab,ti OR 'non-insulin-dependent diabetes mellitus':ab,ti OR 'diabetes mellitus, non-insulin-dependent':ab,ti OR 'diabetes mellitus':ab,ti OR t2dm:ab,ti OR niddm:ab,ti OR dm:ab,ti	490932	7-Jun-25
#5	'non insulin dependent diabetes mellitus'/exp	421468	7-Jun-25
#4	'clinical trial':ab,ti OR 'clinical trials, randomized':ab,ti OR 'trials, randomized clinical':ab,ti OR 'controlled clinical trials, randomized':ab,ti OR 'intervention study':ab,ti	412633	7-Jun-25
#3	'clinical trial'/exp	2551093	7-Jun-25
#2	'medicine, chinese traditional':ab,ti OR tcm:ab,ti OR 'traditional chinese medicine':ab,ti OR 'chinese medicinal herb':ab,ti OR 'chinese herbal medicine':ab,ti OR decoction:ab,ti OR formula:ab,ti OR prescription:ab,ti OR 'chinese patent medicine':ab,ti OR 'chinese patent drug':ab,ti OR 'chinese herbal compound prescription':ab,ti	514860	7-Jun-25
#1	'chinese medicine'/exp	91547	7-Jun-25

History and Search Details

Download Delete

Search	Actions	Details	Query	Results	Time
#13	···	>	Search: (((("Medicine, Chinese Traditional"[Mesh]) OR (Medicine, Chinese Traditional[Title/Abstract] OR TCM[Title/Abstract] OR traditional Chinese medicine[Title/Abstract] OR Chinese medicinal herb[Title/Abstract] OR Chinese herbal medicine[Title/Abstract] OR decoction[Title/Abstract] OR formula[Title/Abstract] OR prescription[Title/Abstract] OR Chinese patent medicine[Title/Abstract] OR Chinese patent drug[Title/Abstract] OR Chinese herbal compound prescription[Title/Abstract])) AND (("Clinical Trial" [Publication Type]) OR (Clinical Trial[Title/Abstract] OR Clinical Trials, Randomized[Title/Abstract] OR Trials, Randomized Clinical[Title/Abstract] OR Controlled Clinical Trials, Randomized[Title/Abstract] OR Intervention Study[Title/Abstract]))) AND (("Diabetes Mellitus, Type 2"[Mesh]) OR (Diabetes Mellitus, Type 2[Title/Abstract] OR Type 2 diabetes mellitus[Title/Abstract] OR Non-insulin-dependent diabetes mellitus[Title/Abstract] OR Diabetes mellitus, non-insulin-dependent[Title/Abstract] OR Diabetes mellitus[Title/Abstract] OR T2DM[Title/Abstract] OR NIDDM[Title/Abstract] OR DM[Title/Abstract]))) AND (("Metformin" [Mesh]) OR (Metformin[Title/Abstract] OR Metformin hydrochloride[Title/Abstract] OR Metformin HCl[Title/Abstract] OR Hydrochloride, metformin[Title/Abstract]))	39	12:30:02
#12	···	>	Search: ("Metformin"[Mesh]) OR (Metformin[Title/Abstract] OR Metformin hydrochloride[Title/Abstract] OR Metformin HCl[Title/Abstract] OR Hydrochloride, metformin[Title/Abstract])	34,300	12:28:49
#11	···	>	Search: ("Diabetes Mellitus, Type 2"[Mesh]) OR (Diabetes Mellitus, Type 2[Title/Abstract] OR Type 2 diabetes mellitus[Title/Abstract] OR Non-insulin-dependent diabetes mellitus[Title/Abstract] OR Diabetes mellitus, non-insulin-dependent[Title/Abstract] OR Diabetes mellitus[Title/Abstract] OR T2DM[Title/Abstract] OR NIDDM[Title/Abstract] OR DM[Title/Abstract])	446,691	12:27:42
#10	···	>	Search: ("Clinical Trial" [Publication Type]) OR (Clinical Trial[Title/Abstract] OR Clinical Trials, Randomized[Title/Abstract] OR Trials, Randomized Clinical[Title/Abstract] OR Controlled Clinical Trials, Randomized[Title/Abstract] OR Intervention Study[Title/Abstract])	1,166,756	12:27:18
#9	···	>	Search: ("Medicine, Chinese Traditional"[Mesh]) OR (Medicine, Chinese Traditional[Title/Abstract] OR TCM[Title/Abstract] OR traditional Chinese medicine[Title/Abstract] OR Chinese medicinal herb[Title/Abstract] OR Chinese herbal medicine[Title/Abstract] OR decoction[Title/Abstract] OR formula[Title/Abstract] OR prescription[Title/Abstract] OR Chinese patent medicine[Title/Abstract] OR Chinese patent drug[Title/Abstract] OR Chinese herbal compound prescription[Title/Abstract])	299,434	12:26:55
#8	···	>	Search: Metformin[Title/Abstract] OR Metformin hydrochloride[Title/Abstract] OR Metformin HCl[Title/Abstract] OR Hydrochloride, metformin[Title/Abstract]	32,349	11:59:12
#7	···	>	Search: "Metformin"[Mesh] Sort by: Most Recent	19,865	11:57:06
#6	···	>	Search: Diabetes Mellitus, Type 2[Title/Abstract] OR Type 2 diabetes mellitus[Title/Abstract] OR Non-insulin-dependent diabetes mellitus[Title/Abstract] OR Diabetes mellitus, non-insulin-dependent[Title/Abstract] OR Diabetes mellitus[Title/Abstract] OR T2DM[Title/Abstract] OR NIDDM[Title/Abstract] OR DM[Title/Abstract]	338,137	11:53:33
#5	···	>	Search: "Diabetes Mellitus, Type 2"[Mesh] Sort by: Most Recent	190,227	11:51:27
#4	···	>	Search: Clinical Trial[Title/Abstract] OR Clinical Trials, Randomized[Title/Abstract] OR Trials, Randomized Clinical[Title/Abstract] OR Controlled Clinical Trials, Randomized[Title/Abstract] OR Intervention Study[Title/Abstract]	252,162	11:30:14
#3	···	>	Search: "Clinical Trial" [Publication Type] Sort by: Most Recent	1,024,154	11:27:23
#2	···	>	Search: Medicine, Chinese Traditional[Title/Abstract] OR TCM[Title/Abstract] OR traditional Chinese medicine[Title/Abstract] OR Chinese medicinal herb[Title/Abstract] OR Chinese herbal medicine[Title/Abstract] OR decoction[Title/Abstract] OR formula[Title/Abstract] OR prescription[Title/Abstract] OR Chinese patent medicine[Title/Abstract] OR Chinese patent drug[Title/Abstract] OR Chinese herbal compound prescription[Title/Abstract]	287,550	11:17:37
#1	···	>	Search: "Medicine, Chinese Traditional"[Mesh] Sort by: Most Recent	26,677	11:08:45

Showing 1 to 13 of 13 entries

NCBI Literature Resources MeSH PMC Bookshelf Disclaimer

The search strategy of pubmed

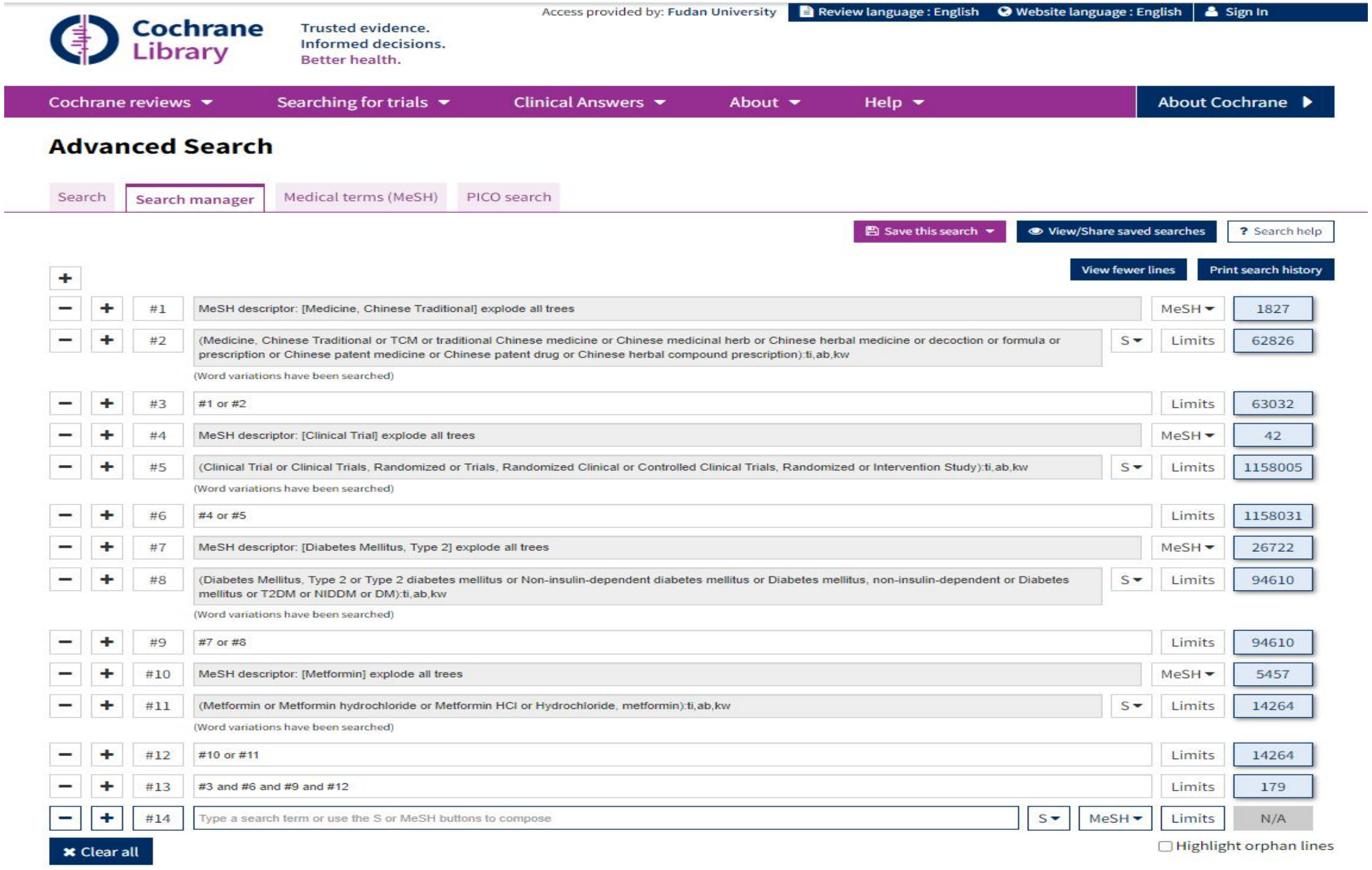

The search strategy of Cochrane Library

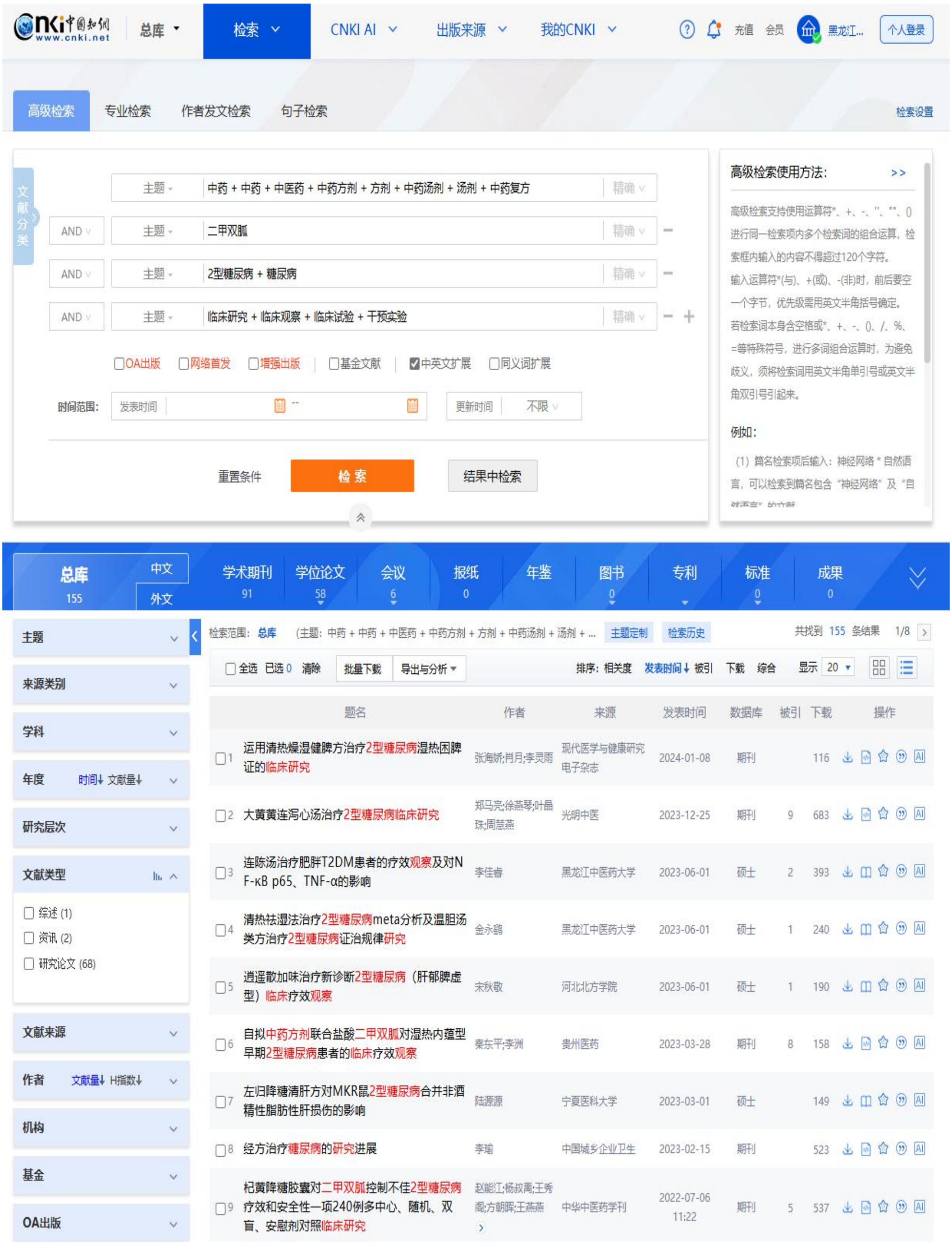

The search strategy of CNKI

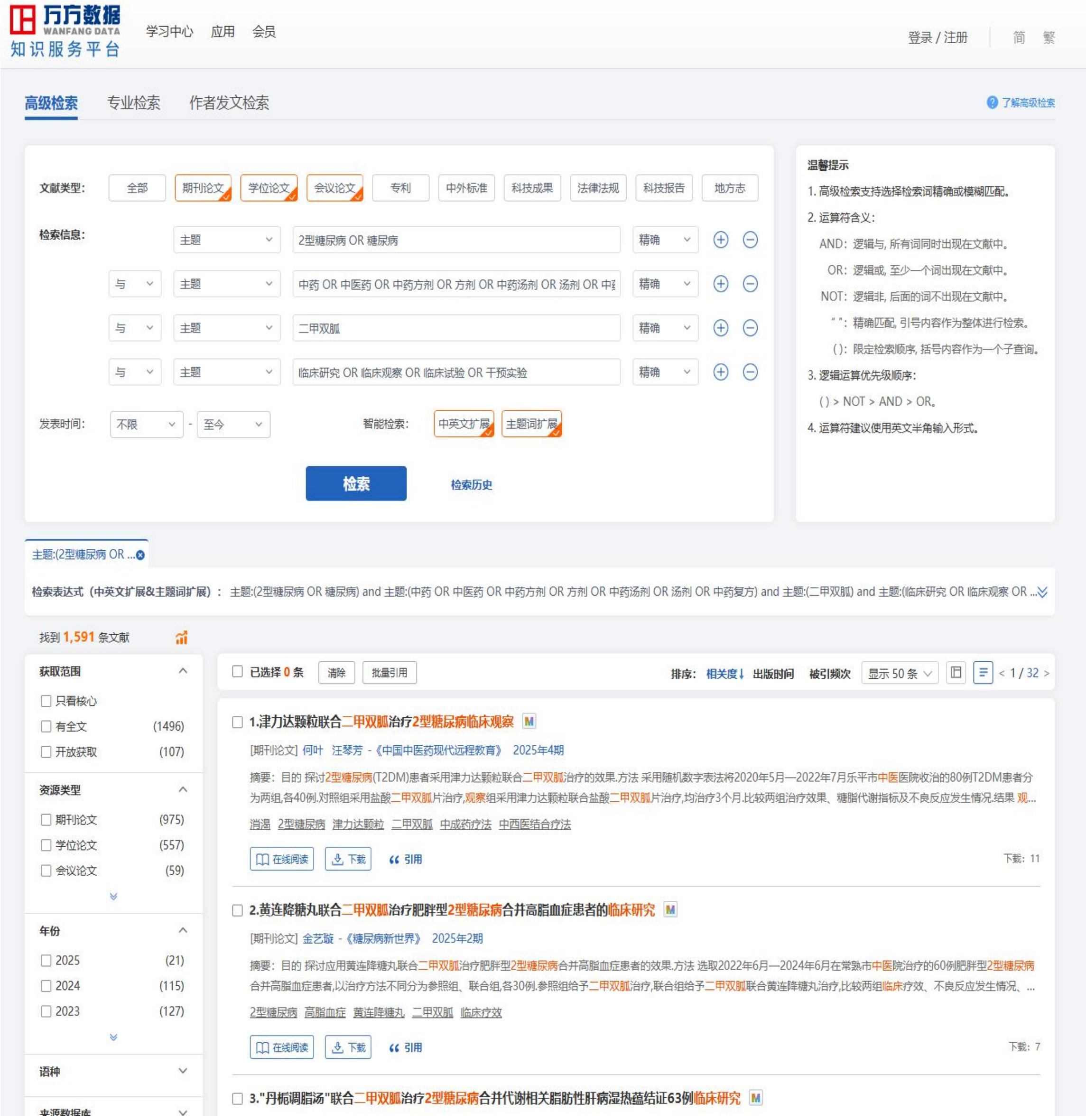

The search strategy of WANFANG

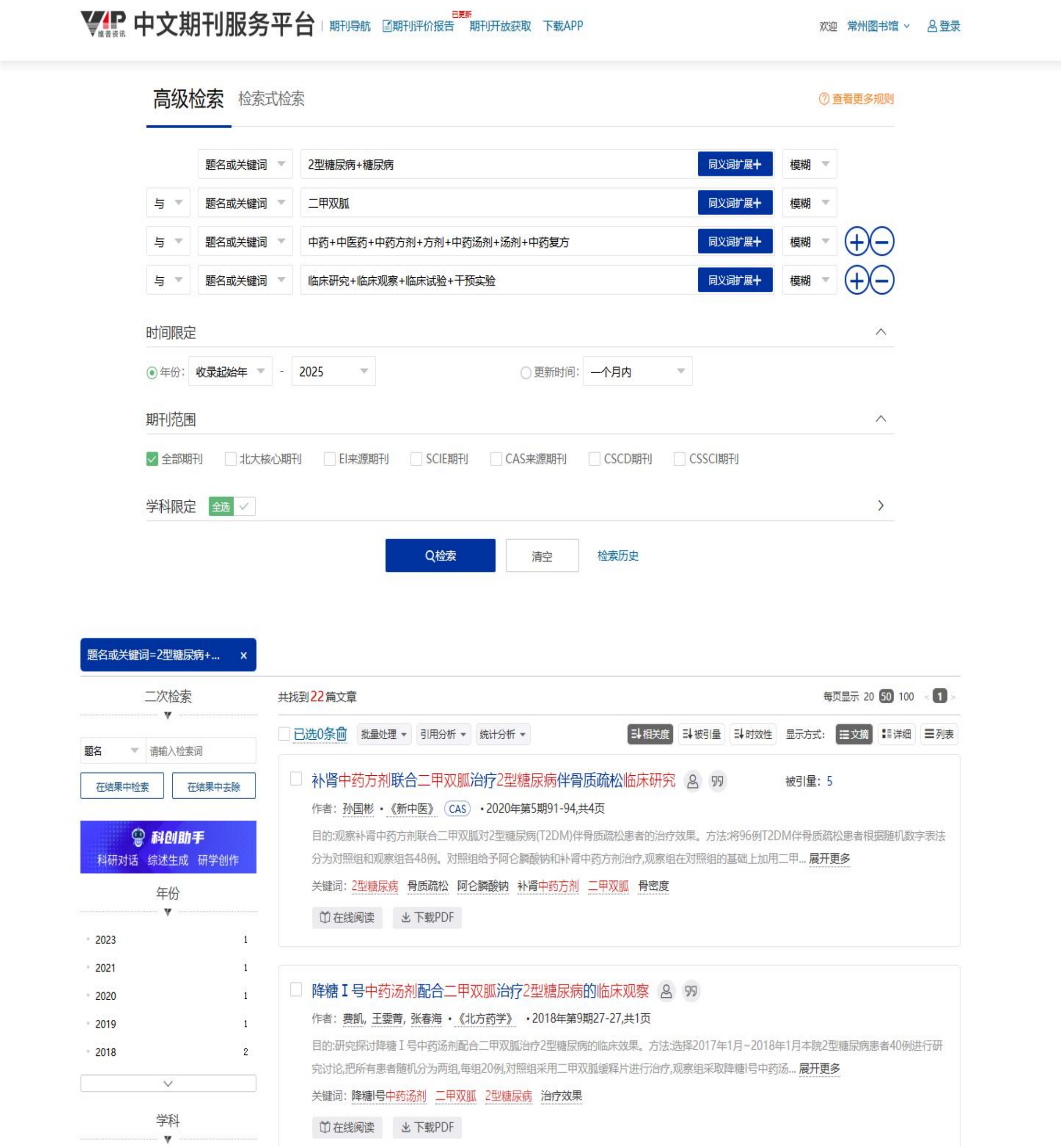

The search strategy of VIP

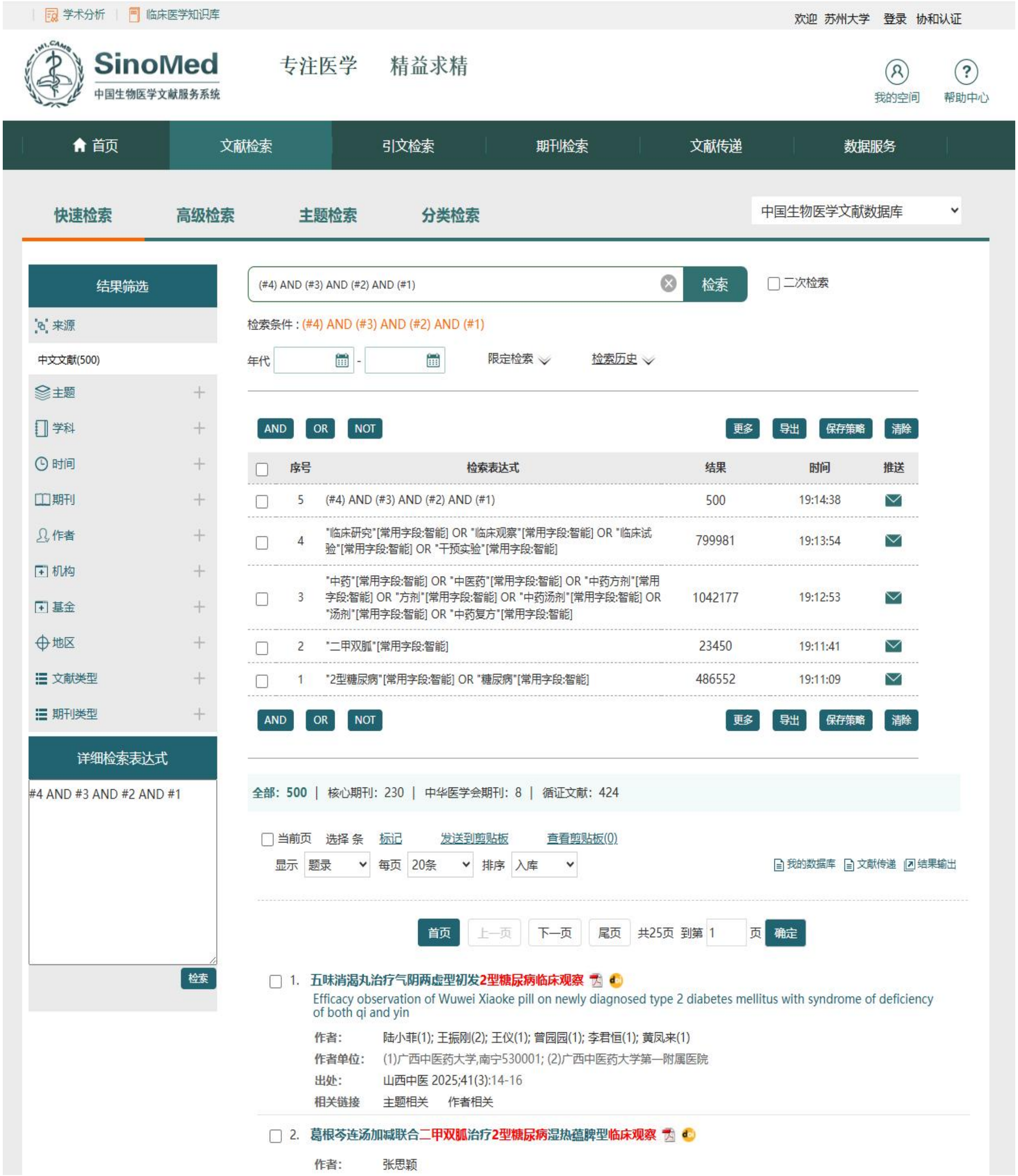

The search strategy of Chinese biomedical literature database
