# Supplementary material for: Efficacy and safety of traditional Chinese classic prescriptions combined with metformin in the treatment of type 2 diabetes mellitus: a Bayesian network meta-analysis
Source: Front Pharmacol. 2026 Feb 11;17:1693378. doi: 10.3389/fphar.2026.1693378 (PMC12932438; doi:10.3389/fphar.2026.1693378)
Supplement: Supplementary file 10 [file DataSheet5.pdf]

R1 曾少武.参苓白术散联合二甲双胍治疗超重/肥胖 2 型糖尿病脾虚湿困证患者的临床疗效观察[D]. 湖北中医药大学,2022. (硕士学位论文,湖北中医药大学).

Shaowu Zeng (2022). Clinical Efficacy Observation of *Shenling Baizhu* Powder Combined with Metformin in Overweight/Obese Type 2 Diabetes Patients with Spleen Deficiency-Dampness Retention Pattern. (Master Dissertation, Hubei University of Chinese Medicine).

DOI:10.27134/d.cnki.ghbzc.2022.000341.

R2 陈春远.知柏地黄汤治疗 2 型糖尿病疗效观察及对 HOMA-IR 的影响[D].广州中医药大学,2015. (硕士学位论文,广州中医药大学).

Chunyuan Chen (2015). Clinical Efficacy Observation of *Zhibai Dihuang* Decoction for Type 2 Diabetes Mellitus and Its Effect on HOMA-IR. (Master Dissertation, Guangzhou university of Chinese Medicine).

R3 陈思思,王雁秋.加味黄连解毒汤联合二甲双胍治疗糖尿病合并肥胖 50 例[J].浙江中医杂志,2021,56(10):711-712.

Sisi Chen (2021). *Huanglian Jiedu* Decoction Combined with Metformin for Diabetes-Obesity Comorbidity: A Clinical Study of 50 Cases. Zhejiang Journal of Traditional Chinese Medicine, 2021, 56(10): 711-712.

DOI:10.13633/j.cnki.zjtcm.2021.10.005..

R4 陈霞.黄连温胆汤加减治疗 T2DM 合并非酒精性脂肪肝痰热互结证的临床观察[D].山东中医药大学,2018. (硕士学位论文,山东中医药大学).

Xia Chen (2018). Clinical Observation on *Huanglian Wendan* Decoction for Type 2 Diabetes Mellitus Complicated by Non-alcoholic Fatty Liver Disease with Phlegm-Heat Intermingling Pattern. (Master Dissertation, Shandong University of Chinese Medicine).

R5 陈鍱.黄连温胆汤联合盐酸二甲双胍缓释片对湿热内蕴型早期 2 型糖尿病患者的临床应用[J].智慧健康,2022,8(13):136-138.

Die Chen (2022). Clinical Application of *Huanglian Wendan* Decoction Combined with Metformin Hydrochloride Sustained-Release Tablets in Early-Stage Type 2 Diabetes Patients with Dampness-Heat Internal Accumulation Pattern. Smart Healthcare, 2022, 8(13): 136-138

DOI:10.19335/j.cnki.2096-1219.2022.13.042.

R6 程梦婕.加减葛根芩连汤联合二甲双胍治疗 2 型糖尿病湿热内蕴型疗效观察[D].湖南中医药大学,2018. (硕士学位论文,湖南中医药大学).

Mengjie Cheng (2018). Therapeutic Efficacy Observation of Modified *Gegen Qinlian* Decoction Combined with Metformin for Type 2 Diabetes Mellitus with Dampness-Heat Internal Accumulation Pattern (Master Dissertation, Hunan University of Chinese Medicine).

R7 崔红艳,陈艳玲.大柴胡汤加味治疗 2 型糖尿病临床观察[J].河北中医,2015,37(08):1195-1197.

Hongyan Cui (2015). Clinical Observation on *Dachaihu* Decoction for Type 2 Diabetes Mellitus. Hebei Journal of Traditional Chinese Medicine, 2015, 37(08): 1195-1197

R8 戴国令.探究采用中药葛根芩连汤治疗糖尿病患者的临床效果[J].糖尿病新世界,2022,25(11):1-4.

Guoling Dai (2022). Clinical Efficacy of *Gegen Qinlian* Decoction in Diabetic Patients: A Therapeutic Investigation. Diabetes New World, 2022, 25(11): 1-4

DOI:10.16658/j.cnki.1672-4062.2022.11.001.

R9 冯佳俊.中药黄连解毒汤治疗 2 型糖尿病患者的临床效果[J].中国处方药,2019,17(04):94-95.

Jiajun Feng (2019). Clinical Efficacy of *Huanglian Jiedu* Decoction in Type 2 Diabetic Patients. Journal of China Prescription Drug, 2019, 17(04): 94-95

R10 冯占荣,徐铁岩,赵乾.白虎加人参汤治疗气阴两虚型 2 型糖尿病 30 例临床观察[J].湖南中医杂志,2020,36(08):51-53.

Zhanrong Feng (2020). Clinical Observation on *Baihu Renshen* Decoction for 30 Cases of Type 2 Diabetes with Qi-Yin Deficiency Pattern. Hunan Journal of Traditional Chinese Medicine, 2020, 36(08): 51-53

DOI:10.16808/j.cnki.issn1003-7705.2020.08.020.

R11 冯召岚.参苓白术散加减联合二甲双胍治疗脾虚痰瘀型 2 型糖尿病临床观察[J].亚太传统医药,2017,13(21):160-161.

Zhaolan Feng (2017). Clinical Observation on *Shenling Baizhu* Powder Combined with Metformin for Type 2 Diabetes with Spleen Deficiency and Phlegm-Stasis Pattern. Asia-Pacific Traditional Medicine, 2017, 13(21): 160-161

R12 付贵珍.葛根芩连汤治疗肥胖 2 型糖尿病（湿热内蕴型）的临床观察[D].河南中医药大学,2017. (硕士学位论文,河南中医药大学).

Guizhen Fu (2017). Clinical Observation on *Gegen Qinlian* Decoction for Obese Type 2 Diabetes with Dampness-Heat Internal Accumulation Pattern (Master Dissertation, Henan University of Chinese Medicine).

R13 龚英,沈玉国,李凯利,等.参苓白术散加减联合二甲双胍治疗脾虚痰瘀型 2 型糖尿病临床观察[J].新疆中医药,2012,30(06):19-21.

Ying Gong (2012). Clinical Observation on *Shenling Baizhu* Powder Combined with Metformin for Type 2 Diabetes with Spleen Deficiency and Phlegm-Stasis Pattern. Xinjiang Journal of Traditional Chinese Medicine, 2012, 30(06): 19-21

R14 姬广慧.黄连温胆汤治疗腹型 2 型糖尿病伴胰岛素抵抗的临床观察[D].山东中医药大学,2017. (硕士学位论文,山东中医药大学).

Guanghui Ji (2017). Clinical Observation on *Huanglian Wendan* Decoction for Central Obesity-Type Type 2 Diabetes with Insulin Resistance. (Master Dissertation, Shandong University of Chinese Medicine).

R15 季聚良,车志英.大柴胡汤加减联合二甲双胍片治疗 2 型糖尿病 20 例[J].中医研究,2020,33(09):18-21.

Juliang Ji (2020). *Dachaihu* Decoction Combined with Metformin Tablets for Type 2 Diabetes: A Clinical Study of 20 Cases. Traditional Chinese Medicinal Research, 2020, 33(09): 18-21

R16 李华.复方葛根芩连汤治疗 2 型糖尿病 48 例临床观察[J].湖南中医杂志,2018,34(08):65-66.

Hua Li (2018). Clinical Observation on Compound *Gegen Qinlian* Decoction for 48 Cases of Type 2 Diabetes Mellitus. Hunan Journal of Traditional Chinese Medicine, 2018, 34(08): 65-66

DOI:10.16808/j.cnki.issn1003-7705.2018.08.029.

R17 李金华,肖琴,熊浪平.加味苓桂术甘汤治疗糖尿病前期临床观察[J].中医临床研究,2024,16(09):81-83.

Jinhua Li (2024). Clinical Observation on *Linggui Zhugan* Decoction for Prediabetes. Clinical Journal of Chinese Medicine, 2024, 16(09): 81-83

R18 李娟娟,冯素云.知柏地黄汤联合西药治疗对 2 型糖尿病患者血糖水平及胰岛

素抵抗的影响[J].中国当代医药,2023,30(21):48-51.
Juanjuan Li (2023). Effects of *Zhibai Dihuang* Decoction Combined with Conventional Medication on Glycemic Control and Insulin Resistance in Type 2 Diabetic Patients. China Contemporary Medicine, 2023, 30(21): 48-51
R19 李勤,吴瑞,郭丰年,等.加味苓桂术甘汤治疗肥胖型 2 型糖尿病[J].吉林中医药,2023,43(07):784-788.
Qin Li (2023). *Linggui Zhugan* Decoction for Obese Type 2 Diabetes. Jilin Journal of Chinese Medicine, 2023, 43(07): 784-788
DOI:10.13463/j.cnki.jlzyy.2023.07.010.
R20 梁厚策,王松林.苓桂术甘汤+干预生活方式联合二甲双胍治疗痰湿壅盛糖尿病肥胖随机平行对照研究[J].实用中医内科杂志,2016,30(11):40-42.
Houce Liang (2016). Randomized Parallel-Controlled Study on *Linggui Zhugan* Decoction plus Lifestyle Intervention Combined with Metformin for Obese Diabetes with Phlegm-Dampness Exuberance. Journal of Practical Traditional Chinese Internal Medicine, 2016, 30(11): 40-42
DOI:10.13729/j.issn.1671-7813.2016.11.17.
R21 罗学林,赵郴.知柏地黄汤联合二甲双胍治疗 2 型糖尿病 52 例临床观察[J].中医药导报,2005,(06):15-16+18.
Xuelin Luo (2005). Clinical Observation on *Zhibai Dihuang* Decoction Combined with Metformin for 52 Cases of Type 2 Diabetes Mellitus. Herald of Traditional Chinese Medicine, 2005, (06): 15-16+18
DOI:10.13862/j.cnki.cn43-1446/r.2005.06.008.
R22 马琳娜,薛毅,王韧舟.葛根芩连汤联合二甲双胍治疗 2 型糖尿病患者的疗效观察[J].世界中西医结合杂志,2022,17(10):2092-2095.
Linna Ma (2022). Therapeutic Efficacy Observation of *Gegen Qinlian* Decoction Combined with Metformin for Type 2 Diabetic Patients. World Journal of Integrated Traditional and Western Medicine, 2022, 17(10): 2092-2095
DOI:10.13935/j.cnki.sjzx.221037.
R23 潘赏赏,李力,王娴.黄连温胆汤联合盐酸二甲双胍对湿热内蕴型早期 2 型糖尿病患者的临床疗效[J].中成药,2021,43(02):557-559.
Shangshang Pan (2021). Clinical Efficacy of *Huanglian Wendan* Decoction Combined

with Metformin Hydrochloride for Early-Stage Type 2 Diabetes Patients with Dampness-Heat Internal Accumulation Pattern. Chinese Traditional Patent Medicine, 2021, 43(02): 557-559

R24 彭少林,汪栋材,张文妍,等.白虎加人参汤治疗气阴两虚、燥热偏盛型初发 2 型糖尿病疗效观察[J].新中医,2015,47(01):84-86.

Shaolin Peng (2015). Efficacy Observation of *Baihu Renshen* Decoction for Newly-Diagnosed Type 2 Diabetes with Qi-Yin Deficiency and Exuberant Dryness-Heat Pattern. Journal of New Chinese Medicine, 2015, 47(01): 84-86

DOI:10.13457/j.cnki.jncm.2015.01.039.

R25 容燕虹.白虎加人参汤治疗糖尿病(肺胃热盛)随机平行对照研究[J].实用中医内科杂志,2019,33(05):32-34.

Yanhong Rong (2019). Randomized Parallel-Controlled Study of *Baihu Renshen* Decoction for Diabetes Mellitus with Intense Lung-Stomach Heat Pattern. Journal of Practical Traditional Chinese Internal Medicine, 2019, 33(05): 32-34

DOI:10.13729/j.issn.1671-7813.z20190196.

R26 覃琴.参苓白术散治疗二甲双胍所致 2 型糖尿病患者腹泻的临床观察研究[D].成都中医药大学,2017. (硕士学位论文,成都中医药大学).

Qin Tan (2017). Clinical Observational Study on *Shenling Baizhu* Powder for Metformin-Induced Diarrhea in Type 2 Diabetic Patients. (Master Dissertation, Chengdu University of Chinese Medicine).

R27 覃琴,谢倩芸,何利黎,等.参苓白术散加减防治 2 型糖尿病用二甲双胍致胃肠道反应临床研究[J].实用中医药杂志,2021,37(06):913-916.

Qin Tan (2021). Clinical Study on *Shenling Baizhu* Powder for Preventing and Treating Metformin-Induced Gastrointestinal Reactions in Type 2 Diabetes. Journal of Practical Traditional Chinese Medicine, 2021, 37(06): 913-916

R28 王冬梅.大柴胡汤加减联合二甲双胍片治疗 2 型糖尿病的临床疗效[J].黑龙江医药科学,2022,45(04):26-28.

Dongmei Wang (2022). Clinical Efficacy of *Dachaihu* Decoction Combined with Metformin Tablets for Type 2 Diabetes Mellitus. Heilongjiang Medicine and Pharmacy, 2022, 45(04): 26-28

R29 王磊.加味葛根芩连汤治疗新诊 2 型糖尿病痰（湿）热互结证临床研究[J].

新中医,2021,53(12):16-20.

Lei Wang (2021). Clinical Study on *Gegen Qinlian* Decoction for Newly-Diagnosed Type 2 Diabetes with Phlegm-Dampness-Heat Intermingling Pattern. Journal of New Chinese Medicine, 2021, 53(12): 16-20

DOI:10.13457/j.cnki.jncm.2021.12.004.

R30 王明坤,程俐,赵鑫,等.黄连温胆汤治疗湿热蕴结型 2 型糖尿病临床研究[J].中西医结合研究,2021,13(06):369-372.

Mingkun Wang (2021). Clinical Study on *Huanglian Wendan* Decoction for Type 2 Diabetes with Dampness-Heat Congestion Pattern. Research of Integrated Traditional and Western Medicine, 2021, 13(06): 369-372

R31 王艳.黄连温胆汤在湿热内蕴型早期 2 型糖尿病患者中的临床应用[J].智慧健康,2022,8(07):124-127.

Yan Wang (2022). Clinical Application of *Huanglian Wendan* Decoction in Early-Stage Type 2 Diabetes Patients with Dampness-Heat Internal Accumulation Pattern. Smart Health, 2022, 8(07): 124-127

DOI:10.19335/j.cnki.2096-1219.2022.07.039.

R32 吴利.葛根芩连汤加减治疗湿热中阻型 2 型糖尿病的临床研究[D].重庆医科大学,2021. (硕士学位论文,重庆医科大学).

Li Wu (2021). Clinical Study on *Gegen Qinlian* Decoction for Type 2 Diabetes with Middle Jiao Dampness-Heat Pattern (Master Dissertation, Chongqing Medical University).

DOI:10.27674/d.cnki.gcyku.2021.000186.

R33 谢芳一.葛根芩连汤辨证加减治疗在 2 型糖尿病患者血糖控制中的效果观察[J].婚育与健康,2023,29(14):112-114.

Fangyi Xie (2023). Therapeutic Effects Observation of Pattern-Based *Gegen Qinlian* Decoction on Glycemic Control in Type 2 Diabetic Patients. Marriage & Health, 2023, 29(14): 112-114

R34 杨文军,王璞.黄连解毒汤对 2 型糖尿病肥胖患者的干预研究[J].山东中医杂志,2013,32(08):535-537.

Wenjun Yang (2013). Therapeutic Intervention Study of *Huanglian Jiedu* Decoction for Obese Patients with Type 2 Diabetes. Shandong Journal of Traditional Chinese

Medicine, 2013, 32(08): 535-537
DOI:10.16295/j.cnki.0257-358x.2013.08.006.

R35 杨雪芹.葛根芩连汤加味联合二甲双胍治疗 2 型糖尿病湿热困脾证的临床研究[D].湖北中医药大学,2021. (硕士学位论文,湖北中医药大学).
Xueqin Yang (2021). Clinical Study on *Gegen Qinlian* Decoction Combined with Metformin for Type 2 Diabetes with Dampness-Heat Encumbering Spleen Pattern (Master Dissertation, Hubei University of Chinese Medicine).
DOI:10.27134/d.cnki.ghbzc.2021.000196.

R36 于慧玲.葛根芩连汤治疗糖尿病患者的临床有效性及安全性探讨[J].智慧健康,2023,9(12):142-146.
Huiling Yu (2023). Clinical Efficacy and Safety Evaluation of *Gegen Qinlian* Decoction for Diabetic Patients. Smart Health, 2023, 9(12): 142-146
DOI:10.19335/j.cnki.2096-1219.2023.12.034.

R37 原红果,董全星,郭连萍.参苓白术散联合二甲双胍治疗 2 型糖尿病肥胖临床观察[J].实用中医药杂志,2023,39(05):978-980.
Hongguo Yuan (2023). Clinical Observation on *Shenling Baizhu* Powder Combined with Metformin for Obese Type 2 Diabetes. Journal of Practical Traditional Chinese Medicine, 2023, 39(05): 978-980

R38 张丽丽.黄连解毒汤联合西药治疗热盛伤津初发 2 型糖尿病随机平行对照研究[J].实用中医内科杂志,2017,31(11):34-37.
Lili Zhang (2017). Randomized Parallel-Controlled Study of *Huanglian Jiedu* Decoction Combined with Conventional Medication for Newly-Diagnosed Type 2 Diabetes with Intense Heat Damaging Fluids Pattern. Journal of Practical Traditional Chinese Internal Medicine, 2017, 31(11): 34-37
DOI:10.13729/j.issn.1671-7813.2017.11.12.

R39 张思颖.葛根芩连汤加减联合二甲双胍治疗 2 型糖尿病湿热蕴脾型临床观察[J].实用中医药杂志,2025,41(02):371-373.
Siying Zhang (2025). Clinical Observation on *Gegen Qinlian* Decoction Combined with Metformin for Type 2 Diabetes with Dampness-Heat Accumulation in Spleen Pattern. Journal of Practical Traditional Chinese Medicine, 2025, 41(02): 371-373

R40 周玉亭.大柴胡汤加味治疗肝胃郁热夹瘀型 2 型糖尿病合并代谢综合征的临

床疗效观察[D].安徽中医药大学,2023. (硕士学位论文,安徽中医药大学).
Yuting Zhou (2023). Clinical Efficacy Observation of *Dachaihu* Decoction for Type 2 Diabetes with Metabolic Syndrome Complicated by Liver-Stomach Stagnant Heat and Blood Stasis Pattern (Master Dissertation, Anhui University of Chinese Medicine).
DOI:10.26922/d.cnki.ganzc.2023.000307.
