# Supplementary material for: Efficacy and safety of traditional Chinese classic prescriptions combined with metformin in the treatment of type 2 diabetes mellitus: a Bayesian network meta-analysis
Source: Front Pharmacol. 2026 Feb 11;17:1693378. doi: 10.3389/fphar.2026.1693378 (PMC12932438; doi:10.3389/fphar.2026.1693378)
Supplement: Supplementary file 11 [file DataSheet12.pdf]

Traditional Chinese Classic Prescriptions combined with Metformin compared to Metformin alone for Type 2 Diabetes Mellitus. (FPG)									
Comparison	Risk of bias	Indirectness	Inconsistency	Imprecision				Publication bias	Certainty of evidence
				Direct comparison		Indirect comparison			
				MD (95%CrIs)	Result	MD (95%CrIs)	Result		
HLJDD+Met vs HLWDD+Met	very serious	Not serious	Not serious			-0.08 (-1.07, 0.93)	serious	Not serious	very low
HLJDD+Met vs DCHD+Met	very serious	Not serious	Not serious			-0.08 (-1.18, 1.03)	very serious	Not serious	very low
HLJDD+Met vs GGQLD+Met	very serious	Not serious	Not serious			-0.11 (-0.98, 0.76)	not serious	Not serious	very low
HLJDD+Met vs BHRSD+Met	very serious	Not serious	Not serious			-0.69 (-1.77, 0.39)	serious	Not serious	very low
HLJDD+Met vs ZBDHD+Met	very serious	Not serious	Not serious			-0.86 (-2, 0.28)	serious	Not serious	very low
HLJDD+Met vs LGZGD+Met	very serious	Not serious	Not serious			-0.87 (-1.94, 0.21)	serious	Not serious	very low
HLJDD+Met vs SLBZP+Met	very serious	Not serious	Not serious			-1.05 (-1.99, -0.1)	serious	Not serious	very low
HLJDD+Met vs Met	very serious	Not serious	Not serious	-1.46 (-2.24, -0.68)	serious			Not serious	very low
HLWDD+Met vs DCHD+Met	very serious	Not serious	Not serious			0 (-1, 1)	serious	Not serious	very low
HLWDD+Met vs GGQLD+Met	very serious	Not serious	Not serious			-0.03 (-0.77, 0.7)	not serious	Not serious	very low
HLWDD+Met vs BHRSD+Met	very serious	Not serious	Not serious			-0.61 (-1.59, 0.36)	serious	Not serious	very low
HLWDD+Met vs ZBDHD+Met	very serious	Not serious	Not serious			-0.78 (-1.84, 0.26)	serious	Not serious	very low
HLWDD+Met vs LGZGD+Met	very serious	Not serious	Not serious			-0.79 (-1.76, 0.17)	serious	Not serious	very low
HLWDD+Met vs SLBZP+Met	very serious	Not serious	Not serious			-0.97 (-1.8, -0.16)	serious	Not serious	very low
HLWDD+Met vs Met	very serious	Not serious	Not serious	-1.39 (-2.01, -0.77)	serious			Not serious	very low
DCHD+Met vs GGQLD+Met	very serious	Not serious	Not serious			-0.03 (-0.91, 0.84)	not serious	Not serious	very low
DCHD+Met vs BHRSD+Met	very serious	Not serious	Not serious			-0.61 (-1.7, 0.47)	serious	Not serious	very low
DCHD+Met vs ZBDHD+Met	very serious	Not serious	Not serious			-0.79 (-1.94, 0.36)	serious	Not serious	very low
DCHD+Met vs LGZGD+Met	very serious	Not serious	Not serious			-0.79 (-1.87, 0.29)	serious	Not serious	very low
DCHD+Met vs SLBZP+Met	very serious	Not serious	Not serious			-0.97 (-1.93, -0.03)	serious	Not serious	very low
DCHD+Met vs Met	very serious	Not serious	very serious	-1.39 (-2.18, -0.6)	serious			Not serious	very low
GGQLD+Met vs BHRSD+Met	very serious	Not serious	Not serious			-0.58 (-1.42, 0.26)	serious	Not serious	very low
GGQLD+Met vs ZBDHD+Met	very serious	Not serious	Not serious			-0.75 (-1.68, 0.17)	serious	Not serious	very low
GGQLD+Met vs LGZGD+Met	very serious	Not serious	Not serious			-0.76 (-1.59, 0.07)	serious	Not serious	very low
GGQLD+Met vs SLBZP+Met	very serious	Not serious	Not serious			-0.94 (-1.6, -0.28)	serious	Not serious	very low
GGQLD+Met vs Met	very serious	Not serious	very serious	-1.35 (-1.74, -0.97)	serious			Not serious	very low
BHRSD+Met vs ZBDHD+Met	very serious	Not serious	Not serious			-0.17 (-1.3, 0.95)	serious	Not serious	very low
BHRSD+Met vs LGZGD+Met	very serious	Not serious	Not serious			-0.18 (-1.23, 0.87)	serious	Not serious	very low
BHRSD+Met vs SLBZP+Met	very serious	Not serious	Not serious			-0.36 (-1.28, 0.56)	serious	Not serious	very low
BHRSD+Met vs Met	very serious	Not serious	Not serious	-0.77 (-1.52, -0.03)	serious			Not serious	very low
ZBDHD+Met vs LGZGD+Met	very serious	Not serious	Not serious			0 (-1.12, 1.11)	serious	Not serious	very low
ZBDHD+Met vs SLBZP+Met	very serious	Not serious	Not serious			-0.19 (-1.18, 0.8)	serious	Not serious	very low
ZBDHD+Met vsMet	very serious	Not serious	Not serious	-0.6 (-1.44, 0.24)	serious			Not serious	very low
LGZGD+Met vs SLBZP+Met	very serious	Not serious	Not serious			-0.18 (-1.1, 0.72)	serious	Not serious	very low
LGZGD+Met vs Met	very serious	Not serious	Not serious	-0.6 (-1.33, 0.14)	serious			Not serious	very low
SLBZP+Met vs Met	very serious	Not serious	Not serious	-0.41 (-0.95, 0.12)	not serious			Not serious	very low

Traditional Chinese Classic Prescriptions combined with Metformin compared to Metformin alone for Type 2 Diabetes Mellitus. (2hPG)									
**Comparison**	**Risk of bias**	**Indirectness**	**Inconsistency**	**Imprecision**				**Publication bias**	**Certainty of evidence**
				**Direct comparison**		**Indirect comparison**			
				**MD (95%CrIs)**	**Result**	**MD (95%CrIs)**	**Result**		
HLJDD+Met vs HLWDD+Met	very serious	Not serious	Not serious			-0.27 (-1.41, 0.86)	Not serious	Not serious	very low
HLJDD+Met vs DCHD+Met	very serious	Not serious	Not serious			0.24 (-0.98, 1.48)	Not serious	Not serious	very low
HLJDD+Met vs GGQLD+Met	very serious	Not serious	Not serious			0.01 (-0.99, 1.01)	Not serious	Not serious	very low
HLJDD+Met vs BHRSD+Met	very serious	Not serious	Not serious			-0.62 (-1.85, 0.62)	Serious	Not serious	very low
HLJDD+Met vs ZBDHD+Met	very serious	Not serious	Not serious			-0.05 (-1.31, 1.21)	Not serious	Not serious	very low
HLJDD+Met vs LGZGD+Met	very serious	Not serious	Not serious			-0.59 (-1.85, 0.68)	Serious	Not serious	very low
HLJDD+Met vs SLBZP+Met	very serious	Not serious	Not serious			-0.75 (-1.81, 0.3)	Serious	Not serious	very low
HLJDD+Met vs Met	very serious	Not serious	Not serious	-1.34 (-2.26, -0.42)	Serious			Not serious	very low
HLWDD+Met vs DCHD+Met	very serious	Not serious	Not serious			-0.03 (-1.08, 1.03)	Not serious	Not serious	very low
HLWDD+Met vs GGQLD+Met	very serious	Not serious	Not serious			-0.25 (-1.04, 0.5)	Not serious	Not serious	very low
HLWDD+Met vs BHRSD+Met	very serious	Not serious	Not serious			-0.88 (-1.96, 0.18)	Serious	Not serious	very low
HLWDD+Met vs ZBDHD+Met	very serious	Not serious	Not serious			-0.32 (-1.42, 0.77)	Not serious	Not serious	very low
HLWDD+Met vs LGZGD+Met	very serious	Not serious	Not serious			-0.85 (-1.95, 0.23)	Serious	Not serious	very low
HLWDD+Met vs SLBZP+Met	very serious	Not serious	Not serious			-1.02 (-1.88, -0.19)	Serious	Not serious	very low
HLWDD+Met vs Met	very serious	Not serious	Not serious	-1.6 (-2.28, -0.95)	Serious			Not serious	very low
DCHD+Met vs GGQLD+Met	very serious	Not serious	Not serious			-0.23 (-1.14, 0.66)	Not serious	Not serious	very low
DCHD+Met vs BHRSD+Met	very serious	Not serious	Not serious			-0.86 (-2.02, 0.31)	Serious	Not serious	very low
DCHD+Met vs ZBDHD+Met	very serious	Not serious	Not serious			-0.29 (-1.49, 0.89)	Not serious	Not serious	very low
DCHD+Met vs LGZGD+Met	very serious	Not serious	Not serious			-0.82 (-2.03, 0.35)	Serious	Not serious	very low
DCHD+Met vs SLBZP+Met	very serious	Not serious	Not serious			-0.99 (-1.97, -0.04)	Serious	Not serious	very low
DCHD+Met vs Met	very serious	Not serious	very serious	-1.58 (-2.4, -0.77)	Serious			Not serious	low
GGQLD+Met vs BHRSD+Met	very serious	Not serious	Not serious			-0.63 (-1.54, 0.3)	Serious	Not serious	very low
GGQLD+Met vs ZBDHD+Met	very serious	Not serious	Not serious			-0.06 (-1, 0.89)	Not serious	Not serious	very low
GGQLD+Met vs LGZGD+Met	very serious	Not serious	Not serious			-0.59 (-1.54, 0.35)	Serious	Not serious	very low
GGQLD+Met vs SLBZP+Met	very serious	Not serious	Not serious			-0.76 (-1.41, -0.12)	Not serious	Not serious	very low
GGQLD+Met vs Met	very serious	Not serious	serious	-1.35 (-1.73, -0.96)	Serious			Not serious	low
BHRSD+Met vs ZBDHD+Met	very serious	Not serious	Not serious			0.57 (-0.64, 1.77)	Serious	Not serious	very low
BHRSD+Met vs LGZGD+Met	very serious	Not serious	Not serious			0.03 (-1.18, 1.22)	Not serious	Not serious	very low
BHRSD+Met vs SLBZP+Met	very serious	Not serious	Not serious			-0.14 (-1.13, 0.84)	Not serious	Not serious	very low
BHRSD+Met vs Met	very serious	Not serious	Not serious	-0.72 (-1.56, 0.1)	Serious			Not serious	very low
ZBDHD+Met vs LGZGD+Met	very serious	Not serious	Not serious			-0.54 (-1.76, 0.68)	Serious	Not serious	very low
ZBDHD+Met vs SLBZP+Met	very serious	Not serious	Not serious			-0.7 (-1.72, 0.3)	Serious	Not serious	very low
ZBDHD+Met vsMet	very serious	Not serious	Not serious	-1.29 (-2.15, -0.42)	Serious			Not serious	very low
LGZGD+Met vs SLBZP+Met	very serious	Not serious	Not serious			-0.17 (-1.17, 0.84)	Not serious	Not serious	very low
LGZGD+Met vs Met	very serious	Not serious	Not serious	-0.75 (-1.61, 0.11)	Serious			Not serious	very low
SLBZP+Met vs Met	very serious	Not serious	very serious	-0.59 (-1.1, -0.07)	Not serious			Not serious	very low

Traditional Chinese Classic Prescriptions combined with Metformin compared to Metformin alone for Type 2 Diabetes Mellitus. (HbA1c)									
Comparison	Risk of bias	Indirectness	Inconsistency	Imprecision				Publication bias	Certainty of evidence
				Direct comparison		Indirect comparison			
				MD (95%CrIs)	Result	MD (95%CrIs)	Result		
HLJDD+Met vs HLWDD+Met	very serious	Not serious	Not serious			-0.19 (-1.23, 0.84)	serious	Not serious	very low
HLJDD+Met vs DCHD+Met	very serious	Not serious	Not serious			0.74 (-0.43, 1.94)	serious	Not serious	very low
HLJDD+Met vs GGQLD+Met	very serious	Not serious	Not serious			0.11 (-0.8, 1.02)	very serious	Not serious	very low
HLJDD+Met vs BHRSD+Met	very serious	Not serious	Not serious			-0.53 (-1.66, 0.59)	very serious	Not serious	very low
HLJDD+Met vs ZBDHD+Met	very serious	Not serious	Not serious			0.11 (-1.02, 1.26)	very serious	Not serious	very low
HLJDD+Met vs LGZGD+Met	very serious	Not serious	Not serious			-0.35 (-1.6, 0.89)	very serious	Not serious	very low
HLJDD+Met vs SLBZP+Met	very serious	Not serious	Not serious			-0.06 (-1.11, 1)	very serious	Not serious	very low
HLJDD+Met vs Met	very serious	Not serious	Not serious	-0.97 (-1.75, -0.19)	serious			Not serious	very low
HLWDD+Met vs DCHD+Met	very serious	Not serious	Not serious			0.94 (-0.17, 2.06)	serious	Not serious	very low
HLWDD+Met vs GGQLD+Met	very serious	Not serious	Not serious			0.3 (-0.52, 1.12)	very serious	Not serious	very low
HLWDD+Met vs BHRSD+Met	very serious	Not serious	Not serious			-0.34 (-1.39, 0.72)	very serious	Not serious	very low
HLWDD+Met vs ZBDHD+Met	very serious	Not serious	Not serious			0.31 (-0.76, 1.38)	very serious	Not serious	very low
HLWDD+Met vs LGZGD+Met	very serious	Not serious	Not serious			-0.16 (-1.34, 1.02)	serious	Not serious	very low
HLWDD+Met vs SLBZP+Met	very serious	Not serious	Not serious			0.14 (-0.84, 1.12)	serious	Not serious	very low
HLWDD+Met vs Met	very serious	Not serious	serious	-0.77 (-1.45, -0.1)	serious			Not serious	very low
DCHD+Met vs GGQLD+Met	very serious	Not serious	Not serious			-0.63 (-1.64, 0.37)	serious	Not serious	very low
DCHD+Met vs BHRSD+Met	very serious	Not serious	Not serious			-1.28 (-2.48, -0.08)	serious	Not serious	very low
DCHD+Met vs ZBDHD+Met	very serious	Not serious	Not serious			-0.63 (-1.85, 0.58)	serious	Not serious	very low
DCHD+Met vs LGZGD+Met	very serious	Not serious	Not serious			-1.1 (-2.43, 0.22)	serious	Not serious	very low
DCHD+Met vs SLBZP+Met	very serious	Not serious	Not serious			-0.8 (-1.94, 0.33)	serious	Not serious	very low
DCHD+Met vs Met	very serious	Not serious	very serious	-1.71 (-2.61, -0.83)	Not serious			Not serious	very low
GGQLD+Met vs BHRSD+Met	very serious	Not serious	Not serious			-0.64 (-1.57, 0.3)	serious	Not serious	very low
GGQLD+Met vs ZBDHD+Met	very serious	Not serious	Not serious			0 (-0.94, 0.96)	very serious	Not serious	very low
GGQLD+Met vs LGZGD+Met	very serious	Not serious	Not serious			-0.46 (-1.54, 0.62)	very serious	Not serious	very low
GGQLD+Met vs SLBZP+Met	very serious	Not serious	Not serious			-0.17 (-1.01, 0.69)	very serious	Not serious	very low
GGQLD+Met vs Met	very serious	Not serious	very serious	-1.08 (-1.54, -0.61)	serious			Not serious	very low
BHRSD+Met vs ZBDHD+Met	very serious	Not serious	Not serious			0.65 (-0.52, 1.81)	very serious	Not serious	very low
BHRSD+Met vs LGZGD+Met	very serious	Not serious	Not serious			0.18 (-1.09, 1.44)	very serious	Not serious	very low
BHRSD+Met vs SLBZP+Met	very serious	Not serious	Not serious			0.48 (-0.6, 1.55)	very serious	Not serious	very low
BHRSD+Met vs Met	very serious	Not serious	serious	-0.43 (-1.25, 0.37)	serious			Not serious	very low
ZBDHD+Met vs LGZGD+Met	very serious	Not serious	Not serious			-0.47 (-1.74, 0.8)	very serious	Not serious	very low
ZBDHD+Met vs SLBZP+Met	very serious	Not serious	Not serious			-0.17 (-1.26, 0.92)	very serious	Not serious	very low
ZBDHD+Met vsMet	very serious	Not serious	serious	-1.08 (-1.91, -0.26)	serious			Not serious	very low
LGZGD+Met vs SLBZP+Met	very serious	Not serious	Not serious			0.3 (-0.91, 1.5)	very serious	Not serious	very low
LGZGD+Met vs Met	very serious	Not serious	Not serious	-0.61 (-1.59, 0.36)	serious			Not serious	very low
SLBZP+Met vs Met	very serious	Not serious	very serious	-0.91 (-1.62, -0.21)	serious			Not serious	very low

Traditional Chinese Classic Prescriptions combined with Metformin compared to Metformin alone for Type 2 Diabetes Mellitus. (TC)									
Comparison	Risk of bias	Indirectness	Inconsistency	Imprecision				Publication bias	Certainty of evidence
				Direct comparison		Indirect comparison			
				MD (95%CrIs)	Result	MD (95%CrIs)	Result		
HLJDD+Met vs HLWDD+Met	very serious	Not serious	Not serious			-1.21 (-2.09, -0.35)	serious	Not serious	very low
HLJDD+Met vs GGQLD+Met	very serious	Not serious	Not serious			-0.79 (-1.59, -0.03)	serious	Not serious	very low
HLJDD+Met vs BHRSD+Met	very serious	Not serious	Not serious			-0.99 (-2.07, 0.09)	serious	Not serious	very low
HLJDD+Met vs LGZGD+Met	very serious	Not serious	Not serious			-0.43 (-1.41, 0.51)	very serious	Not serious	very low
HLJDD+Met vs SLBZP+Met	very serious	Not serious	Not serious			-0.92 (-1.73, -0.13)	serious	Not serious	very low
HLJDD+Met vs Met	very serious	Not serious	Not serious	-1.37 (-2.05, -0.7)	Not serious			Not serious	very low
HLWDD+Met vs GGQLD+Met	very serious	Not serious	Not serious			0.42 (-0.27, 1.08)	serious	Not serious	very low
HLWDD+Met vs BHRSD+Met	very serious	Not serious	Not serious			0.22 (-0.77, 1.23)	very serious	Not serious	very low
HLWDD+Met vs LGZGD+Met	very serious	Not serious	Not serious			0.78 (-0.1, 1.65)	serious	Not serious	very low
HLWDD+Met vs SLBZP+Met	very serious	Not serious	Not serious			0.3 (-0.4, 0.98)	serious	Not serious	very low
HLWDD+Met vs Met	very serious	Not serious	Not serious	-0.16 (-0.7, 0.39)	serious			Not serious	very low
GGQLD+Met vs BHRSD+Met	very serious	Not serious	Not serious			-0.19 (-1.1, 0.74)	very serious	Not serious	very low
GGQLD+Met vs LGZGD+Met	very serious	Not serious	Not serious			0.36 (-0.43, 1.15)	serious	Not serious	very low
GGQLD+Met vs SLBZP+Met	very serious	Not serious	Not serious			-0.12 (-0.7, 0.45)	serious	Not serious	very low
GGQLD+Met vs Met	very serious	Not serious	serious	-0.58 (-0.95, -0.18)	serious			Not serious	low
BHRSD+Met vs LGZGD+Met	very serious	Not serious	Not serious			0.56 (-0.54, 1.63)	very serious	Not serious	very low
BHRSD+Met vs SLBZP+Met	very serious	Not serious	Not serious			0.07 (-0.87, 1.01)	very serious	Not serious	very low
BHRSD+Met vs Met	very serious	Not serious	Not serious	-0.38 (-1.22, 0.46)	serious			Not serious	very low
LGZGD+Met vs SLBZP+Met	very serious	Not serious	Not serious			-0.49 (-1.29, 0.33)	serious	Not serious	very low
LGZGD+Met vs Met	very serious	Not serious	very serious	-0.94 (-1.62, -0.24)	serious			Not serious	low
SLBZP+Met vs Met	very serious	Not serious	very serious	-0.45 (-0.87, -0.02)	serious			Not serious	low

Traditional Chinese Classic Prescriptions combined with Metformin compared to Metformin alone for Type 2 Diabetes Mellitus. (TG)									
Comparison	Risk of bias	Indirectness	Inconsistency	Imprecision				Publication bias	Certainty of evidence
				Direct comparison		Indirect comparison			
				MD (95%CrIs)	Result	MD (95%CrIs)	Result		
HLJDD+Met vs HLWDD+Met	very serious	Not serious	Not serious			-0.28 (-1.2, 0.65)	very serious	Not serious	very low
HLJDD+Met vs DCHD+Met	very serious	Not serious	Not serious			-0.47 (-1.33, 0.36)	serious	Not serious	very low
HLJDD+Met vs GGQLD+Met	very serious	Not serious	Not serious			-0.27 (-1.13, 0.58)	very serious	Not serious	very low
HLJDD+Met vs BHRSD+Met	very serious	Not serious	Not serious			-0.65 (-1.86, 0.54)	very serious	Not serious	very low
HLJDD+Met vs LGZGD+Met	very serious	Not serious	Not serious			0.43 (-0.7, 1.4)	very serious	Not serious	very low
HLJDD+Met vs SLBZP+Met	very serious	Not serious	Not serious			-0.47 (-1.33, 0.36)	serious	Not serious	very low
HLJDD+Met vs Met	very serious	Not serious	very serious	-0.61 (-1.32, 0.08)	serious			Not serious	low
HLWDD+Met vs DCHD+Met	very serious	Not serious	Not serious			-0.02 (-1.18, 1.12)	very serious	Not serious	very low
HLWDD+Met vs GGQLD+Met	very serious	Not serious	Not serious			0.01 (-0.78, 0.78)	very serious	Not serious	very low
HLWDD+Met vs BHRSD+Met	very serious	Not serious	Not serious			-0.37 (-1.52, 0.76)	very serious	Not serious	very low
HLWDD+Met vs LGZGD+Met	very serious	Not serious	Not serious			0.71 (-0.35, 1.62)	serious	Not serious	very low
HLWDD+Met vs SLBZP+Met	very serious	Not serious	Not serious			-0.19 (-0.97, 0.56)	very serious	Not serious	very low
HLWDD+Met vs Met	very serious	Not serious	Not serious	-0.33 (-0.94, 0.27)	serious			Not serious	very low
DCHD+Met vs GGQLD+Met	very serious	Not serious	Not serious			0.03 (-1.06, 1.13)	very serious	Not serious	very low
DCHD+Met vs BHRSD+Met	very serious	Not serious	Not serious			-0.35 (-1.73, 1.03)	very serious	Not serious	very low
DCHD+Met vs LGZGD+Met	very serious	Not serious	Not serious			0.73 (-0.59, 1.9)	very serious	Not serious	very low
DCHD+Met vs SLBZP+Met	very serious	Not serious	Not serious			-0.17 (-1.26, 0.91)	very serious	Not serious	very low
DCHD+Met vs Met	very serious	Not serious	Not serious	-0.31 (-1.29, 0.67)	very serious			Not serious	very low
GGQLD+Met vs BHRSD+Met	very serious	Not serious	Not serious			-0.38 (-1.47, 0.71)	very serious	Not serious	very low
GGQLD+Met vs LGZGD+Met	very serious	Not serious	Not serious			0.7 (-0.29, 1.55)	serious	Not serious	very low
GGQLD+Met vs SLBZP+Met	very serious	Not serious	Not serious			-0.2 (-0.89, 0.48)	serious	Not serious	very low
GGQLD+Met vs Met	very serious	Not serious	serious	-0.34 (-0.83, 0.15)	serious			Not serious	low
BHRSD+Met vs LGZGD+Met	very serious	Not serious	Not serious			1.08 (-0.23, 2.25)	serious	Not serious	very low
BHRSD+Met vs SLBZP+Met	very serious	Not serious	Not serious			0.18 (-0.91, 1.26)	very serious	Not serious	very low
BHRSD+Met vs Met	very serious	Not serious	Not serious	0.04 (-0.93, 1.01)	very serious			Not serious	very low
LGZGD+Met vs SLBZP+Met	very serious	Not serious	Not serious			-0.9 (-1.75, 0.08)	serious	Not serious	very low
LGZGD+Met vs Met	very serious	Not serious	very serious	-1.04 (-1.75, -0.19)	serious			Not serious	low
SLBZP+Met vs Met	very serious	Not serious	very serious	-0.14 (-0.61, 0.34)	serious			Not serious	low

Traditional Chinese Classic Prescriptions combined with Metformin compared to Metformin alone for Type 2 Diabetes Mellitus. (LDL-c)									
Comparison	Risk of bias	Indirectness	Inconsistency	Imprecision				Publication bias	Certainty of evidence
				Direct comparison		Indirect comparison			
				MD (95%CrIs)	Result	MD (95%CrIs)	Result		
HLJDD+Met vs HLWDD+Met	very serious	Not serious	Not serious			-1 (-1.78, -0.23)	serious	Not serious	very low
HLJDD+Met vs DCHD+Met	very serious	Not serious	Not serious			-0.92 (-2.02, 0.11)	serious	Not serious	very low
HLJDD+Met vs GGQLD+Met	very serious	Not serious	Not serious			-0.78 (-1.53, -0.09)	serious	Not serious	very low
HLJDD+Met vs BHRSD+Met	very serious	Not serious	Not serious			-1.06 (-2.09, -0.11)	serious	Not serious	very low
HLJDD+Met vs LGZGD+Met	very serious	Not serious	Not serious			-0.93 (-1.83, -0.1)	serious	Not serious	very low
HLJDD+Met vs SLBZP+Met	very serious	Not serious	Not serious			-0.81 (-1.68, -0.02)	serious	Not serious	very low
HLJDD+Met vs Met	very serious	Not serious	very serious	-1.22 (-1.85, -0.66)	Not serious			Not serious	very low
HLWDD+Met vs DCHD+Met	very serious	Not serious	Not serious			0.08 (-0.97, 1.06)	very serious	Not serious	very low
HLWDD+Met vs GGQLD+Met	very serious	Not serious	Not serious			0.23 (-0.46, 0.84)	serious	Not serious	very low
HLWDD+Met vs BHRSD+Met	very serious	Not serious	Not serious			-0.05 (-1.03, 0.84)	very serious	Not serious	very low
HLWDD+Met vs LGZGD+Met	very serious	Not serious	Not serious			0.07 (-0.76, 0.85)	very serious	Not serious	very low
HLWDD+Met vs SLBZP+Met	very serious	Not serious	Not serious			0.2 (-0.61, 0.92)	very serious	Not serious	very low
HLWDD+Met vs Met	very serious	Not serious	very serious	-0.22 (-0.76, 0.25)	serious			Not serious	very low
DCHD+Met vs GGQLD+Met	very serious	Not serious	Not serious			0.14 (-0.83, 1.12)	very serious	Not serious	very low
DCHD+Met vs BHRSD+Met	very serious	Not serious	Not serious			-0.14 (-1.32, 1.04)	very serious	Not serious	very low
DCHD+Met vs LGZGD+Met	very serious	Not serious	Not serious			-0.01 (-1.09, 1.08)	very serious	Not serious	very low
DCHD+Met vs SLBZP+Met	very serious	Not serious	Not serious			0.12 (-0.95, 1.17)	very serious	Not serious	very low
DCHD+Met vs Met	very serious	Not serious	Not serious	-0.3 (-1.19, 0.58)	very serious			Not serious	very low
GGQLD+Met vs BHRSD+Met	very serious	Not serious	Not serious			-0.28 (-1.17, 0.61)	very serious	Not serious	very low
GGQLD+Met vs LGZGD+Met	very serious	Not serious	Not serious			-0.15 (-0.9, 0.6)	very serious	Not serious	very low
GGQLD+Met vs SLBZP+Met	very serious	Not serious	Not serious			-0.03 (-0.74, 0.67)	very serious	Not serious	very low
GGQLD+Met vs Met	very serious	Not serious	Not serious	-0.44 (-0.86, -0.03)	serious			Not serious	very low
BHRSD+Met vs LGZGD+Met	very serious	Not serious	Not serious			0.13 (-0.87, 1.14)	very serious	Not serious	very low
BHRSD+Met vs SLBZP+Met	very serious	Not serious	Not serious			0.25 (-0.73, 1.23)	very serious	Not serious	very low
BHRSD+Met vs Met	very serious	Not serious	Not serious	-0.16 (-0.95, 0.63)	very serious			Not serious	very low
LGZGD+Met vs SLBZP+Met	very serious	Not serious	Not serious			0.13 (-0.73, 0.97)	very serious	Not serious	very low
LGZGD+Met vs Met	very serious	Not serious	Not serious	-0.29 (-0.92, 0.33)	serious			Not serious	very low
SLBZP+Met vs Met	very serious	Not serious	Not serious	-0.41 (-0.98, 0.16)	serious			Not serious	very low

Traditional Chinese Classic Prescriptions combined with Metformin compared to Metformin alone for Type 2 Diabetes Mellitus. (HDL-c)									
**Comparison**	**Risk of bias**	**Indirectness**	**Inconsistency**	**Imprecision**				**Publication bias**	**Certainty of evidence**
				**Direct comparison**		**Indirect comparison**			
				**MD (95%CrIs)**	**Result**	**MD (95%CrIs)**	**Result**		
HLJDD+Met vs HLWDD+Met	very serious	Not serious	Not serious			0.19 (-0.26, 0.62)	very serious	Not serious	very low
HLJDD+Met vs DCHD+Met	very serious	Not serious	Not serious			0.3 (-0.2, 0.81)	serious	Not serious	very low
HLJDD+Met vs GGQLD+Met	very serious	Not serious	Not serious			0.04 (-0.35, 0.4)	very serious	Not serious	very low
HLJDD+Met vs BHRSD+Met	very serious	Not serious	Not serious			0.24 (-0.26, 0.74)	very serious	Not serious	very low
HLJDD+Met vs LGZGD+Met	very serious	Not serious	Not serious			0.29 (-0.21, 0.79)	very serious	Not serious	very low
HLJDD+Met vs Met	very serious	Not serious	Not serious	0.34 (0.05, 0.63)	serious			Not serious	very low
HLWDD+Met vs DCHD+Met	very serious	Not serious	Not serious			0.11 (-0.41, 0.64)	very serious	Not serious	very low
HLWDD+Met vs GGQLD+Met	very serious	Not serious	Not serious			-0.14 (-0.56, 0.25)	very serious	Not serious	very low
HLWDD+Met vs BHRSD+Met	very serious	Not serious	Not serious			0.05 (-0.46, 0.58)	very serious	Not serious	very low
HLWDD+Met vs LGZGD+Met	very serious	Not serious	Not serious			0.1 (-0.41, 0.63)	very serious	Not serious	very low
HLWDD+Met vs Met	very serious	Not serious	Not serious	0.15 (-0.16, 0.48)	serious			Not serious	very low
DCHD+Met vs GGQLD+Met	very serious	Not serious	Not serious			-0.26 (-0.75, 0.2)	serious	Not serious	very low
DCHD+Met vs BHRSD+Met	very serious	Not serious	Not serious			-0.06 (-0.63, 0.52)	very serious	Not serious	very low
DCHD+Met vs LGZGD+Met	very serious	Not serious	Not serious			-0.01 (-0.59, 0.58)	very serious	Not serious	very low
DCHD+Met vs Met	very serious	Not serious	Not serious	0.04 (-0.37, 0.45)	very serious			Not serious	very low
GGQLD+Met vs BHRSD+Met	very serious	Not serious	Not serious			0.2 (-0.25, 0.68)	very serious	Not serious	very low
GGQLD+Met vs LGZGD+Met	very serious	Not serious	Not serious			0.25 (-0.21, 0.74)	very serious	Not serious	very low
GGQLD+Met vs Met	very serious	Not serious	very serious	0.3 (0.07, 0.55)	serious			Not serious	low
BHRSD+Met vs LGZGD+Met	very serious	Not serious	Not serious			0.05 (-0.52, 0.62)	very serious	Not serious	very low
BHRSD+Met vs Met	very serious	Not serious	Not serious	0.1 (-0.3, 0.5)	very serious			Not serious	very low
LGZGD+Met vs Met	very serious	Not serious	Not serious	0.05 (-0.36, 0.46)	very serious			Not serious	very low

Traditional Chinese Classic Prescriptions combined with Metformin compared to Metformin alone for Type 2 Diabetes Mellitus. (FINS)									
**Comparison**	**Risk of bias**	**Indirectness**	**Inconsistency**	**Imprecision**				**Publication bias**	**Certainty of evidence**
				**Direct comparison**		**Indirect comparison**			
				**MD (95%CrIs)**	**Result**	**MD (95%CrIs)**	**Result**		
HLJDD+Met vs HLWDD+Met	very serious	Not serious	Not serious			1.76 (-0.52, 4.34)	serious	Not serious	very low
HLJDD+Met vs GGQLD+Met	very serious	Not serious	Not serious			1.25 (-1.15, 3.64)	very serious	Not serious	very low
HLJDD+Met vs ZBDHD+Met	very serious	Not serious	Not serious			8.85 (5.35, 12.39)	Not serious	Not serious	very low
HLJDD+Met vs LGZGD+Met	very serious	Not serious	Not serious			-0.14 (-3.76, 3.54)	very serious	Not serious	very low
HLJDD+Met vs SLBZP+Met	very serious	Not serious	Not serious			6.06 (2.55, 9.58)	Not serious	Not serious	very low
HLJDD+Met vs Met	very serious	Not serious	Not serious	-0.94 (-2.99, 1.14)	very serious			Not serious	very low
HLWDD+Met vs GGQLD+Met	very serious	Not serious	Not serious			-0.49 (-2.47, 1.03)	very serious	Not serious	very low
HLWDD+Met vs ZBDHD+Met	very serious	Not serious	Not serious			7.09 (3.82, 10.09)	Not serious	Not serious	very low
HLWDD+Met vs LGZGD+Met	very serious	Not serious	Not serious			-1.9 (-5.26, 1.24)	very serious	Not serious	very low
HLWDD+Met vs SLBZP+Met	very serious	Not serious	Not serious			4.28 (1.11, 7.29)	Not serious	Not serious	very low
HLWDD+Met vs Met	very serious	Not serious	serious	-2.69 (-4.12, -1.61)	Not serious			Not serious	very low
GGQLD+Met vs ZBDHD+Met	very serious	Not serious	Not serious			7.59 (4.5, 10.75)	Not serious	Not serious	very low
GGQLD+Met vs LGZGD+Met	very serious	Not serious	Not serious			-1.39 (-4.58, 1.89)	very serious	Not serious	very low
GGQLD+Met vs SLBZP+Met	very serious	Not serious	Not serious			4.8 (1.74, 7.89)	Not serious	Not serious	very low
GGQLD+Met vs Met	very serious	Not serious	very serious	-2.2 (-3.38, -0.95)	very serious			Not serious	low
ZBDHD+Met vs LGZGD+Met	very serious	Not serious	Not serious			-8.98 (-13.13, -4.82)	Not serious	Not serious	very low
ZBDHD+Met vs SLBZP+Met	very serious	Not serious	Not serious			-2.8 (-6.79, 1.24)	very serious	Not serious	very low
ZBDHD+Met vsMet	very serious	Not serious	Not serious	-9.79 (-12.66, -6.93)	Not serious			Not serious	very low
LGZGD+Met vs SLBZP+Met	very serious	Not serious	Not serious			6.18 (2.09, 10.31)	very serious	Not serious	very low
LGZGD+Met vs Met	very serious	Not serious	Not serious	-0.81 (-3.82, 2.17)	very serious			Not serious	very low
SLBZP+Met vs Met	very serious	Not serious	Not serious	-6.99 (-9.82, -4.17)	very serious			Not serious	very low

Traditional Chinese Classic Prescriptions combined with Metformin compared to Metformin alone for Type 2 Diabetes Mellitus. (HOMA-IR)									
**Comparison**	**Risk of bias**	**Indirectness**	**Inconsistency**	**Imprecision**				**Publication bias**	**Certainty of evidence**
				**Direct comparison**		**Indirect comparison**			
				**MD (95%CI)**	**Result**	**MD (95%CI)**	**Result**		
HLJDD+Met vs HLWDD+Met	very serious	Not serious	Not serious			-0.64 (-10.35, 9.08)	very serious	Not serious	very low
HLJDD+Met vs DCHD+Met	very serious	Not serious	Not serious			-0.39 (-12.24, 11.46)	very serious	Not serious	very low
HLJDD+Met vs GGQLD+Met	very serious	Not serious	Not serious			2.22 (-5.68, 10.19)	very serious	Not serious	very low
HLJDD+Met vs ZBDHD+Met	very serious	Not serious	Not serious			1.13 (-8.6, 10.86)	very serious	Not serious	very low
HLJDD+Met vs LGZGD+Met	very serious	Not serious	Not serious			0.34 (-11.57, 12.28)	very serious	Not serious	very low
HLJDD+Met vs SLBZP+Met	very serious	Not serious	Not serious			0.01 (-8.82, 8.85)	very serious	Not serious	very low
HLJDD+Met vs Met	very serious	Not serious	very serious	-1.09 (-7.94, 5.76)	very serious			Not serious	low
HLWDD+Met vs DCHD+Met	very serious	Not serious	Not serious			0.23 (-11.58, 12.08)	very serious	Not serious	very low
HLWDD+Met vs GGQLD+Met	very serious	Not serious	Not serious			2.86 (-5.05, 10.83)	very serious	Not serious	very low
HLWDD+Met vs ZBDHD+Met	very serious	Not serious	Not serious			1.77 (-7.94, 11.48)	very serious	Not serious	very low
HLWDD+Met vs LGZGD+Met	very serious	Not serious	Not serious			0.97 (-10.94, 12.88)	very serious	Not serious	very low
HLWDD+Met vs SLBZP+Met	very serious	Not serious	Not serious			0.64 (-8.17, 9.51)	very serious	Not serious	very low
HLWDD+Met vs Met	very serious	Not serious	Not serious	-0.46 (-7.33, 6.37)	very serious			Not serious	very low
DCHD+Met vs GGQLD+Met	very serious	Not serious	Not serious			2.62 (-7.8, 13.14)	very serious	Not serious	very low
DCHD+Met vs ZBDHD+Met	very serious	Not serious	Not serious			1.53 (-10.27, 13.33)	very serious	Not serious	very low
DCHD+Met vs LGZGD+Met	very serious	Not serious	Not serious			0.74 (-13.02, 14.51)	very serious	Not serious	very low
DCHD+Met vs SLBZP+Met	very serious	Not serious	Not serious			0.39 (-10.73, 11.59)	very serious	Not serious	very low
DCHD+Met vs Met	very serious	Not serious	Not serious	-0.69 (-10.3, 8.98)	very serious			Not serious	very low
GGQLD+Met vs ZBDHD+Met	very serious	Not serious	Not serious			-1.1 (-9.07, 6.83)	very serious	Not serious	very low
GGQLD+Met vs LGZGD+Met	very serious	Not serious	Not serious			-1.89 (-12.39, 8.64)	very serious	Not serious	very low
GGQLD+Met vs SLBZP+Met	very serious	Not serious	Not serious			-2.21 (-9.07, 4.62)	very serious	Not serious	very low
GGQLD+Met vs Met	very serious	Not serious	very serious	-3.31 (-7.33, 0.65)	very serious			Not serious	low
ZBDHD+Met vs LGZGD+Met	very serious	Not serious	Not serious			-0.8 (-12.66, 11.13)	very serious	Not serious	very low
ZBDHD+Met vs SLBZP+Met	very serious	Not serious	Not serious			-1.12 (-9.95, 7.72)	very serious	Not serious	very low
ZBDHD+Met vsMet	very serious	Not serious	very serious	-2.24 (-9.04, 4.62)	very serious			Not serious	low
LGZGD+Met vs SLBZP+Met	very serious	Not serious	Not serious			-0.32 (-11.55, 10.88)	very serious	Not serious	very low
LGZGD+Met vs Met	very serious	Not serious	Not serious	-1.43 (-11.16, 8.28)	very serious			Not serious	very low
SLBZP+Met vs Met	very serious	Not serious	Not serious	-1.1 (-6.7, 4.5)	very serious			Not serious	very low
