# Supplementary material for: Efficacy and safety of traditional Chinese classic prescriptions combined with metformin in the treatment of type 2 diabetes mellitus: a Bayesian network meta-analysis
Source: Front Pharmacol. 2026 Feb 11;17:1693378. doi: 10.3389/fphar.2026.1693378 (PMC12932438; doi:10.3389/fphar.2026.1693378)
Supplement: Supplementary file 12 [file DataSheet8.pdf]

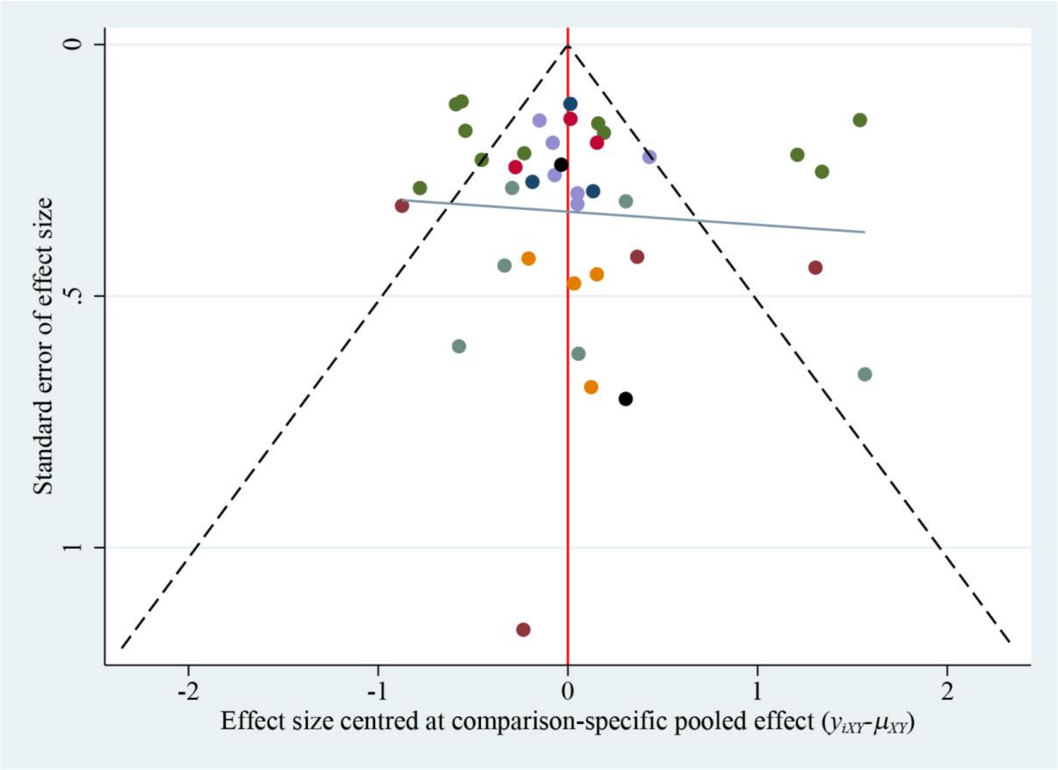

Comparison-correction funnel plot (FPG)

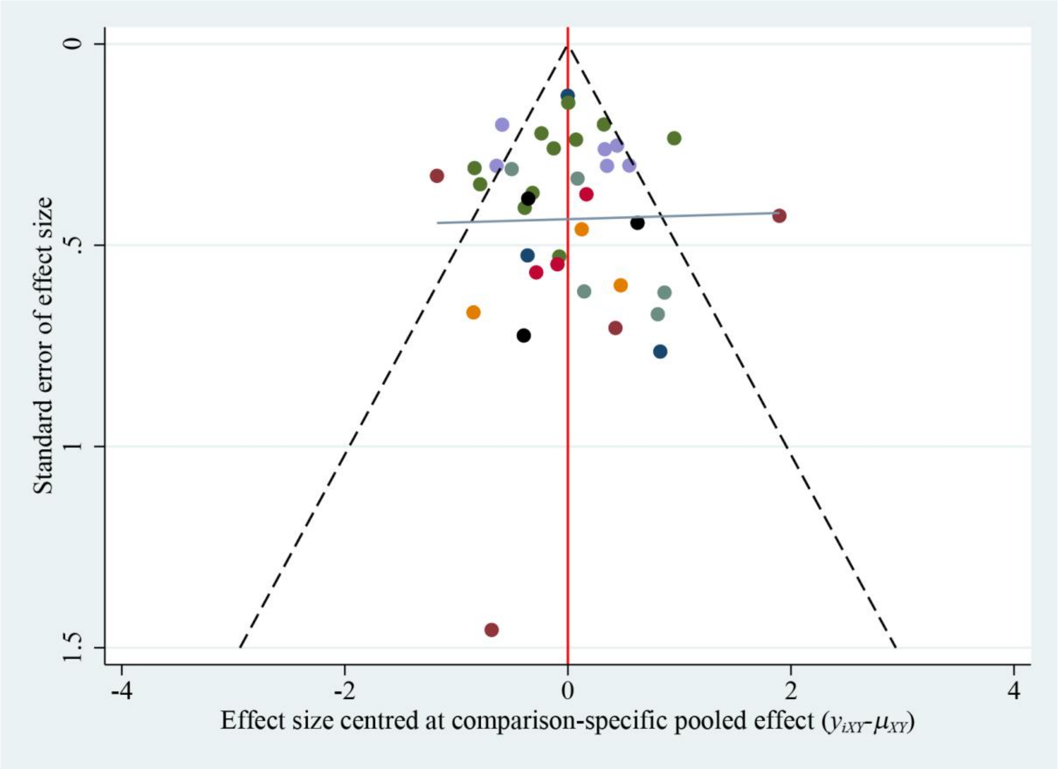

Comparison-correction funnel plot (2hPG)

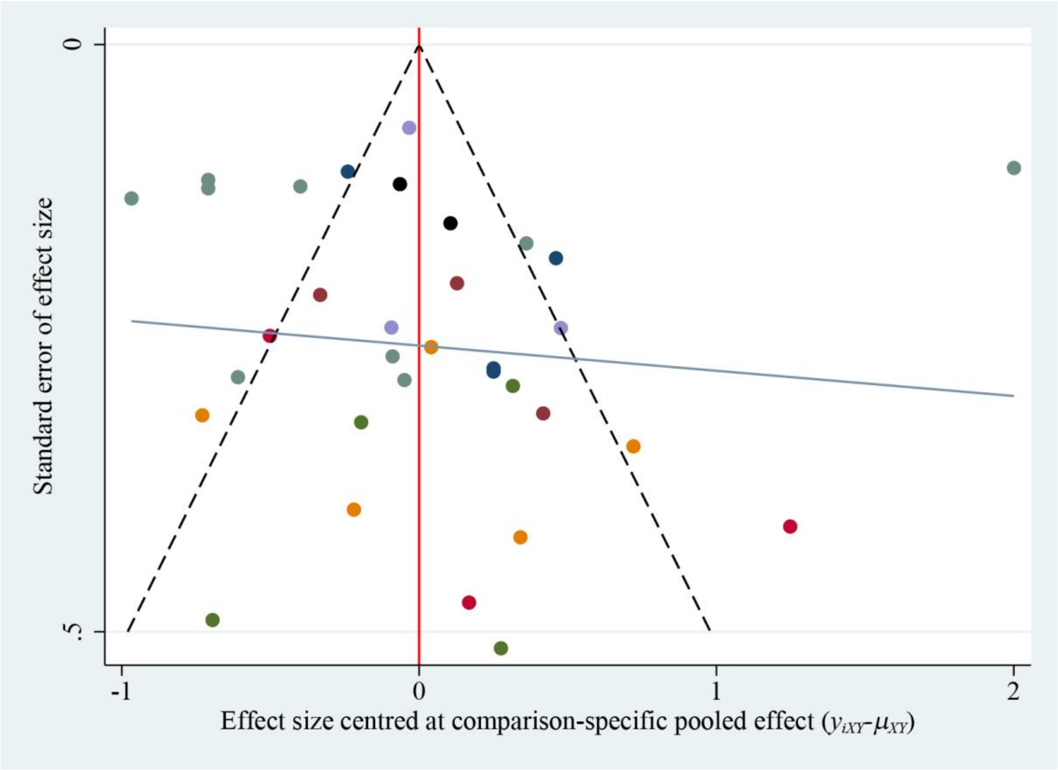

Comparison-correction funnel plot (HbA1c)

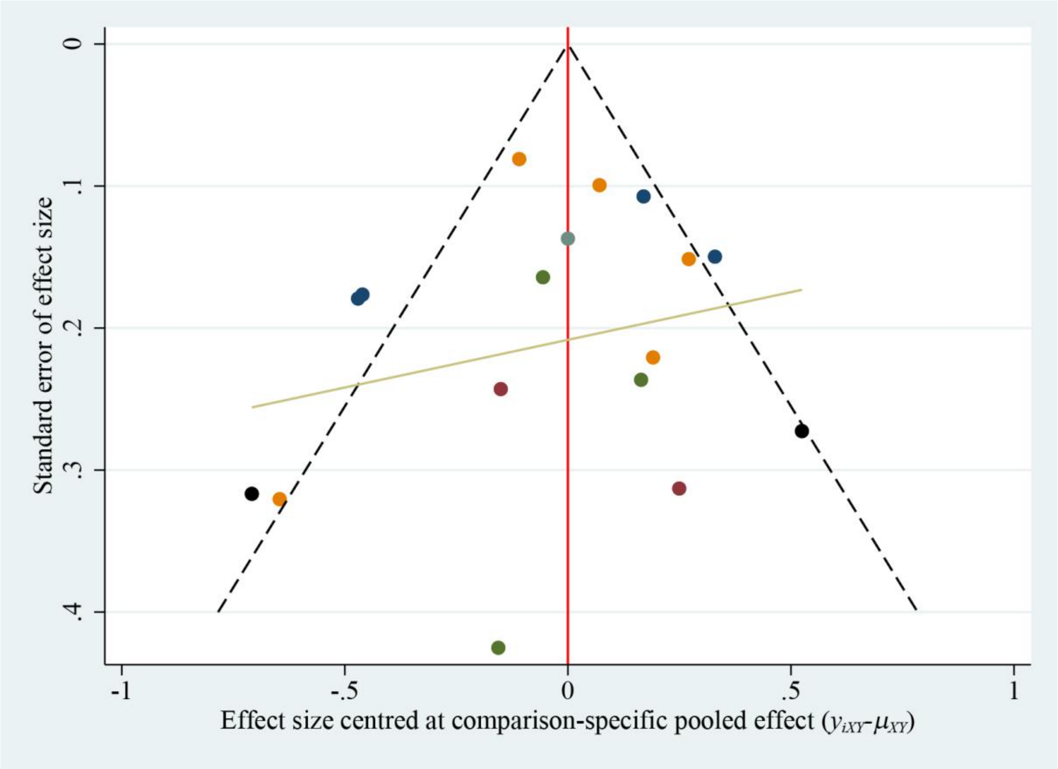

Comparison-correction funnel plot (TC)

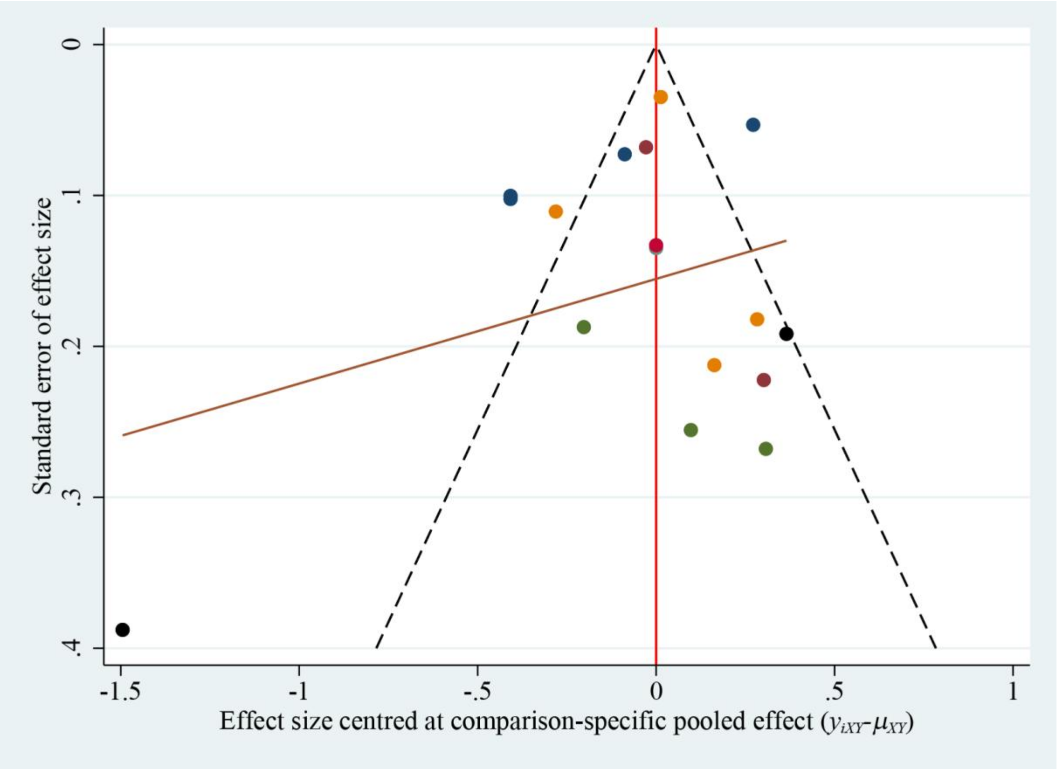

Comparison-correction funnel plot (TG)

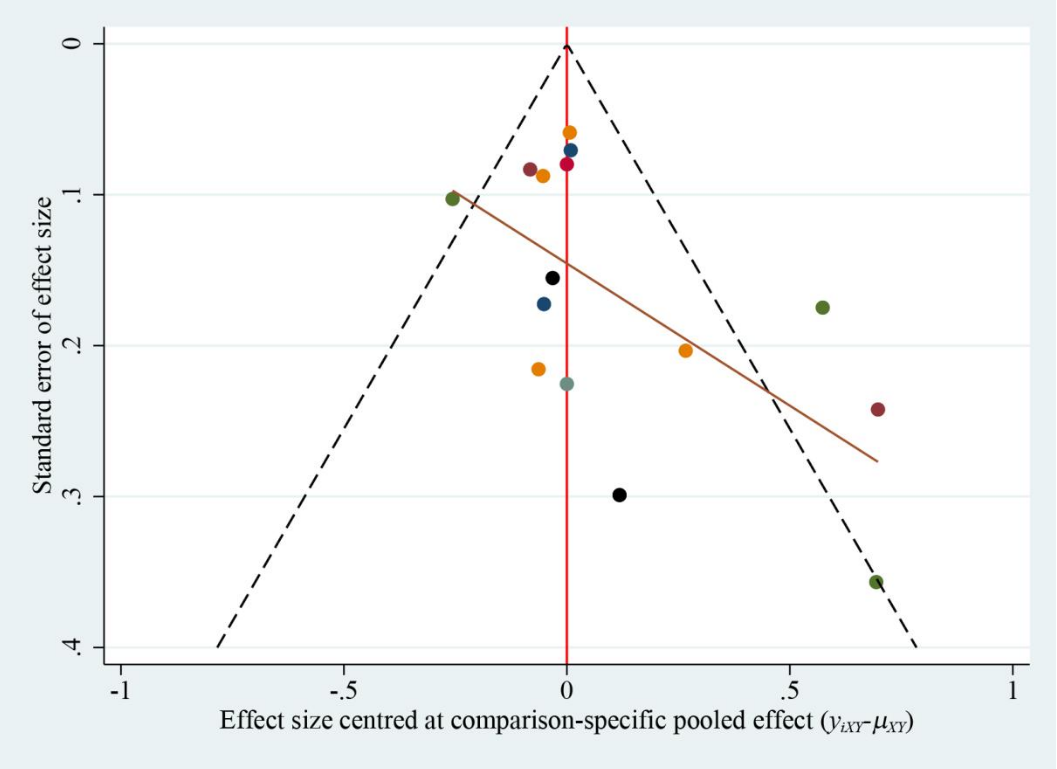

Comparison-correction funnel plot (LDL-c)

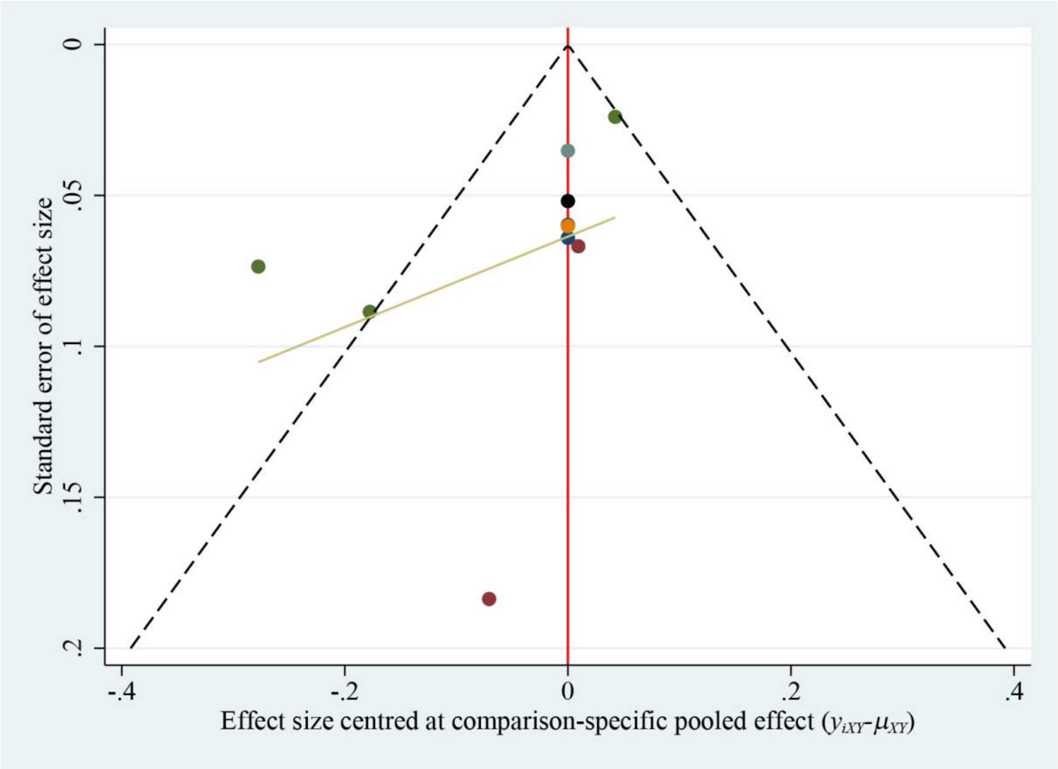

Comparison-correction funnel plot (HDL-c)

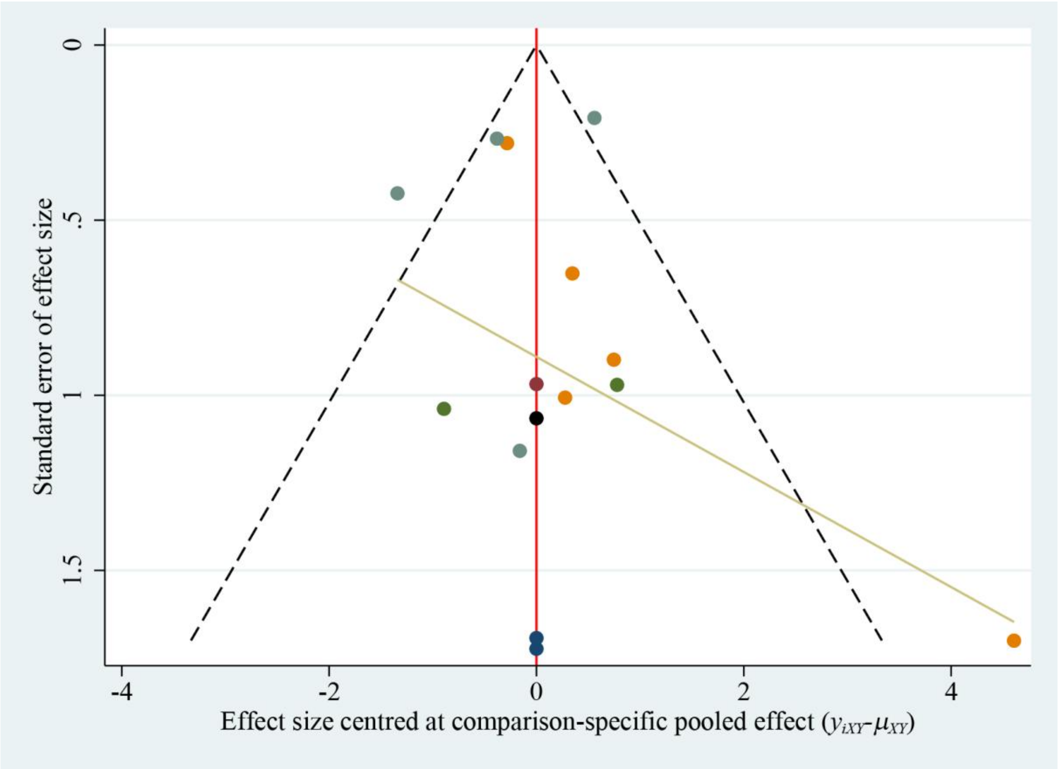

Comparison-correction funnel plot (FINS)

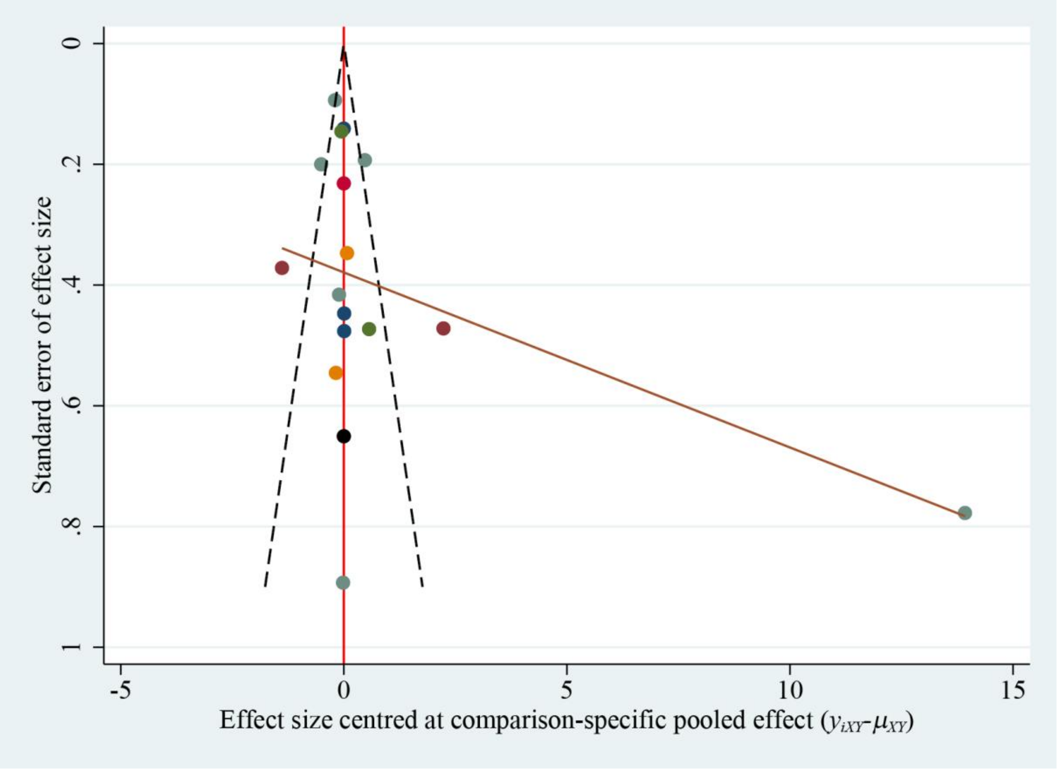

Comparison-correction funnel plot (HOMA-IR)
