# Supplementary material for: Efficacy and safety of traditional Chinese classic prescriptions combined with metformin in the treatment of type 2 diabetes mellitus: a Bayesian network meta-analysis
Source: Front Pharmacol. 2026 Feb 11;17:1693378. doi: 10.3389/fphar.2026.1693378 (PMC12932438; doi:10.3389/fphar.2026.1693378)

Network Contribution Matrix (FPG)

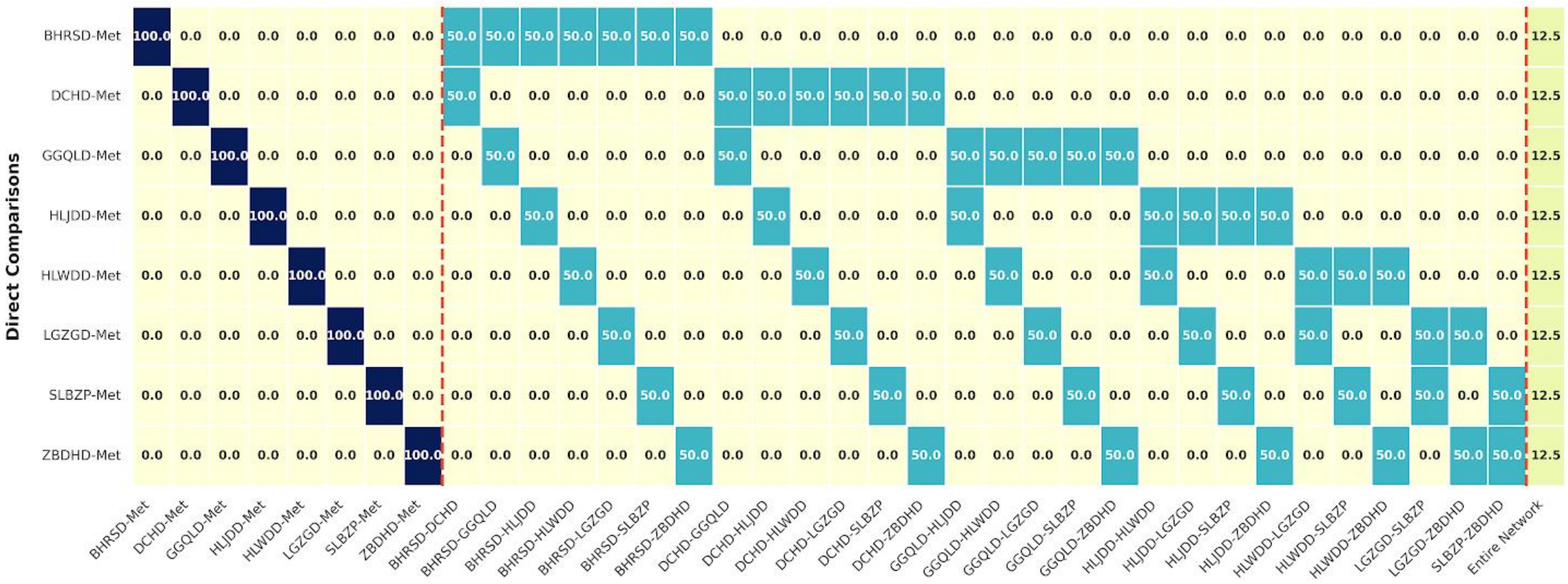

Network Contribution Matrix (HbA1c)

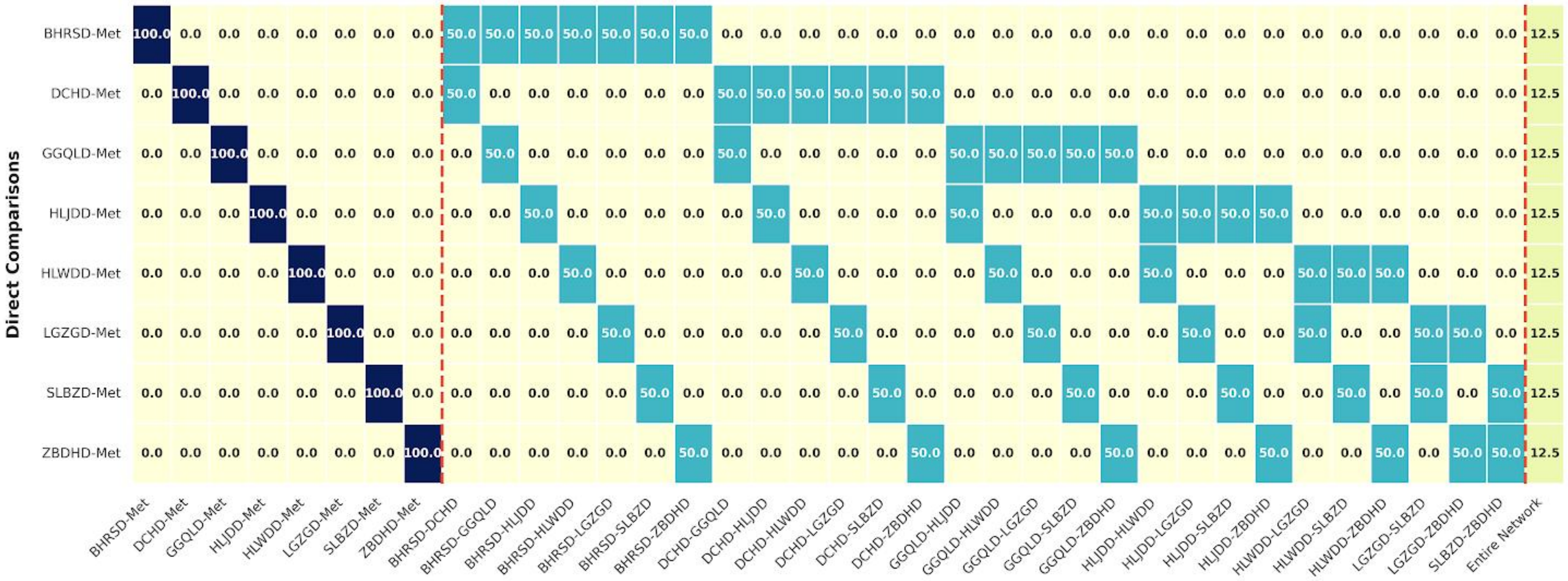

Network Contribution Matrix (2hPG)

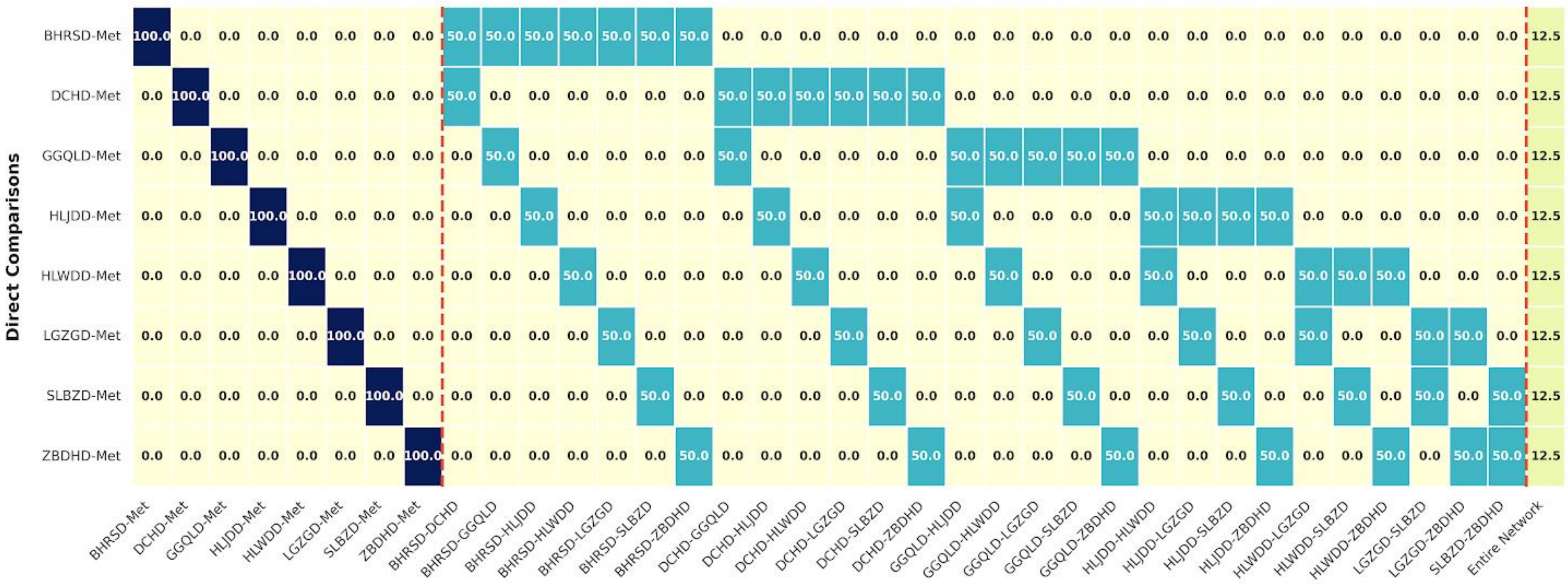

Network Contribution Matrix (TC)

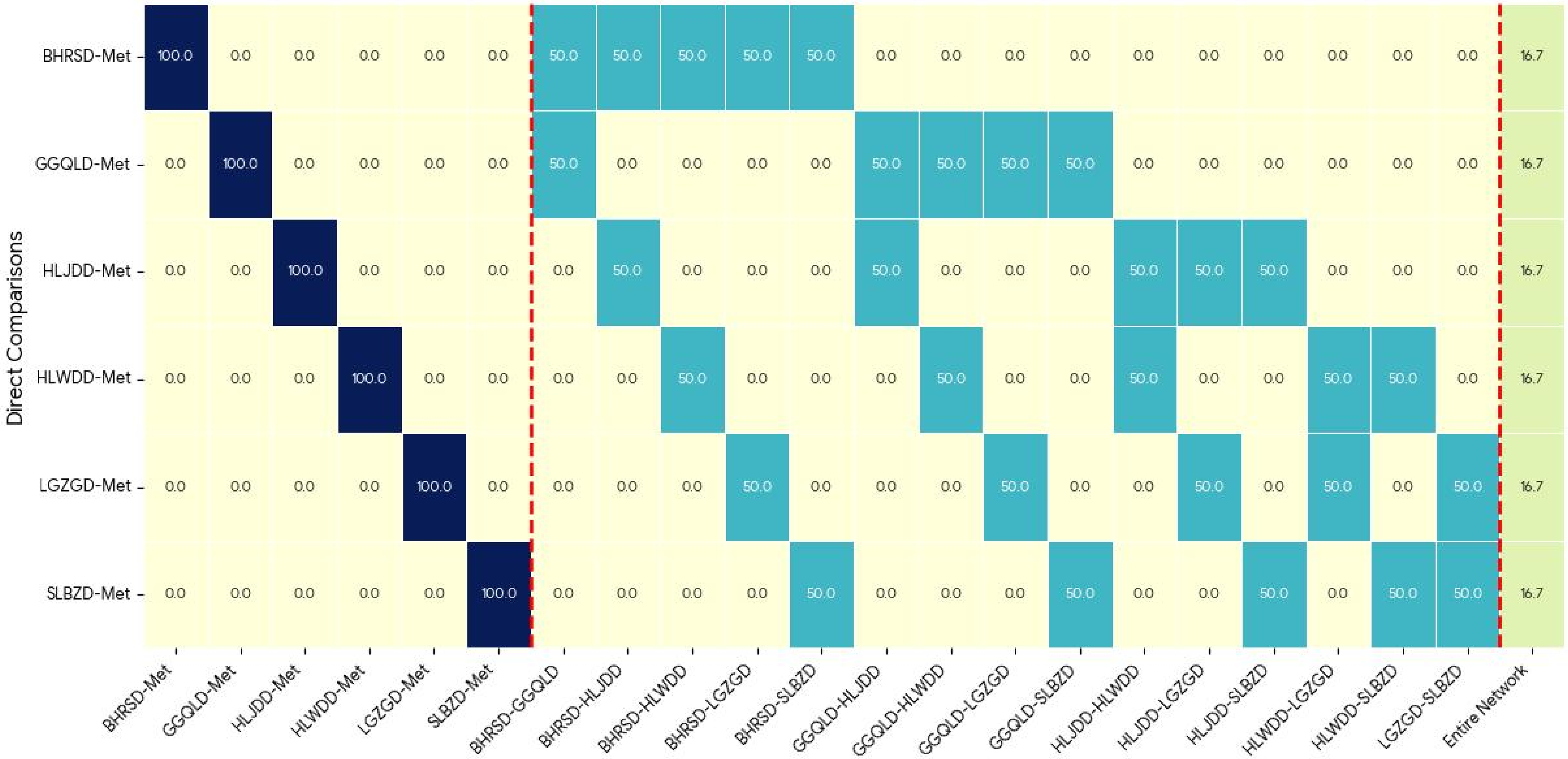

Network Contribution Matrix (TG)

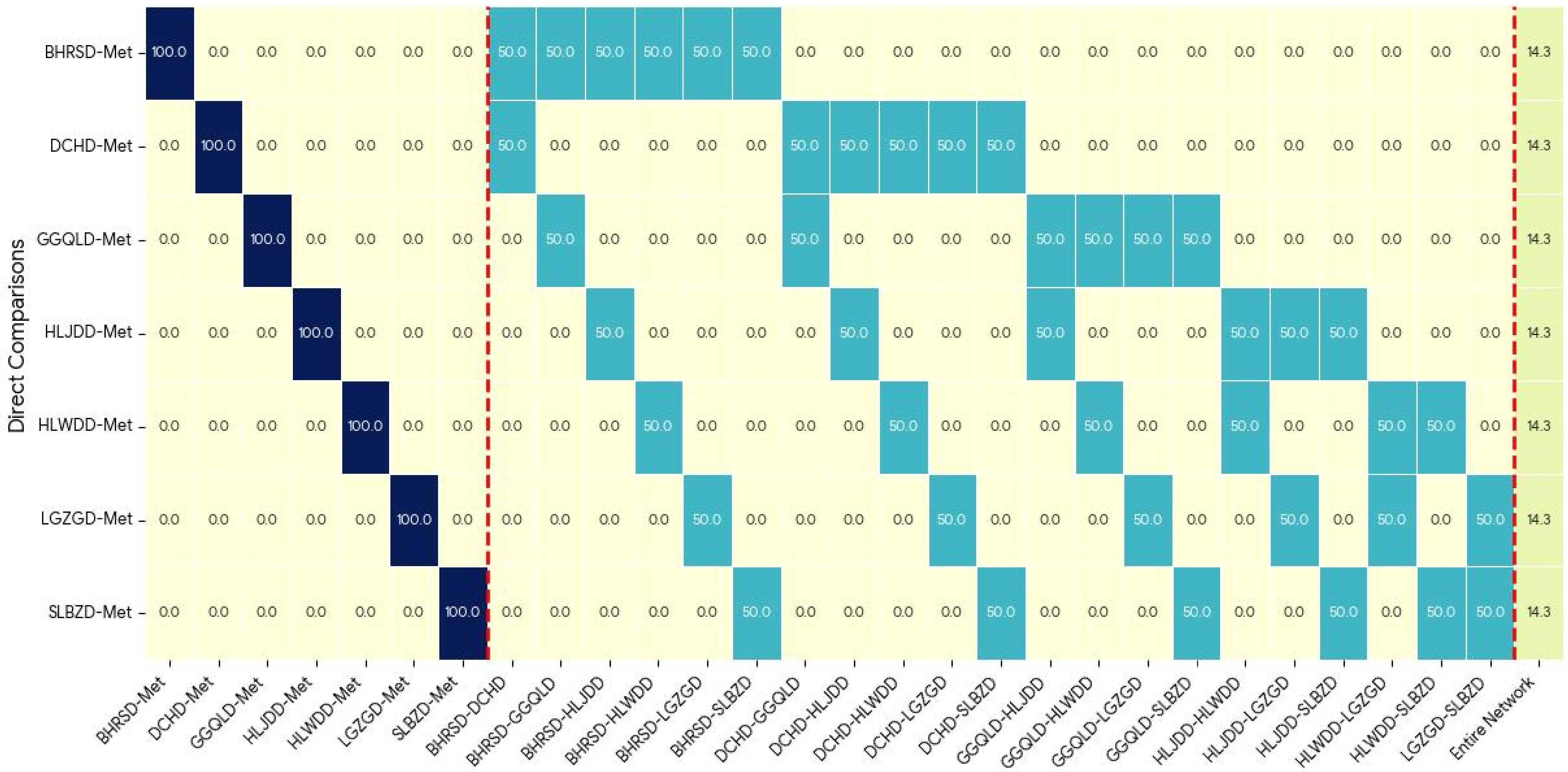

Network Contribution Matrix (LDL-c)

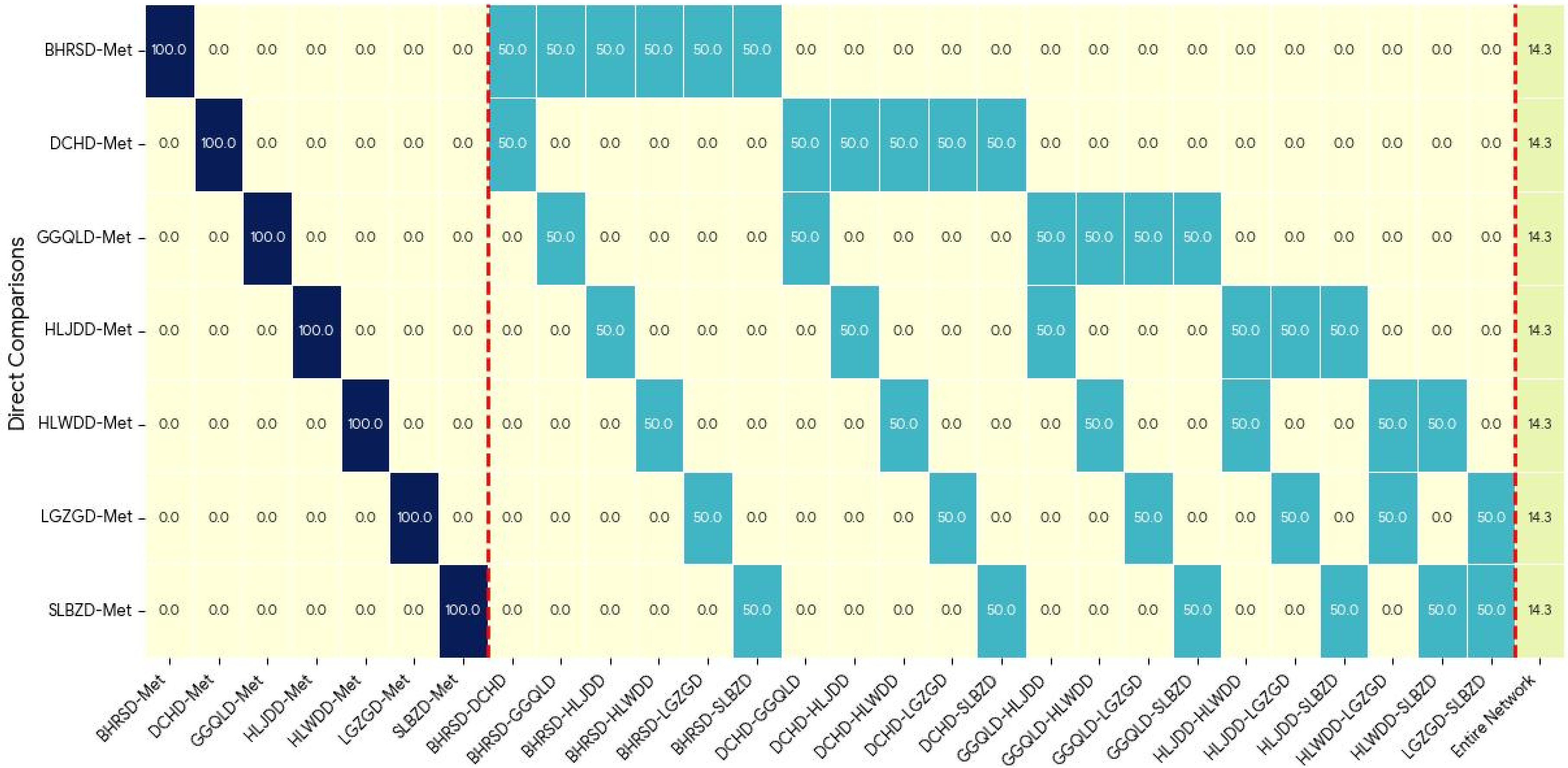

Network Contribution Matrix (HDL-c)

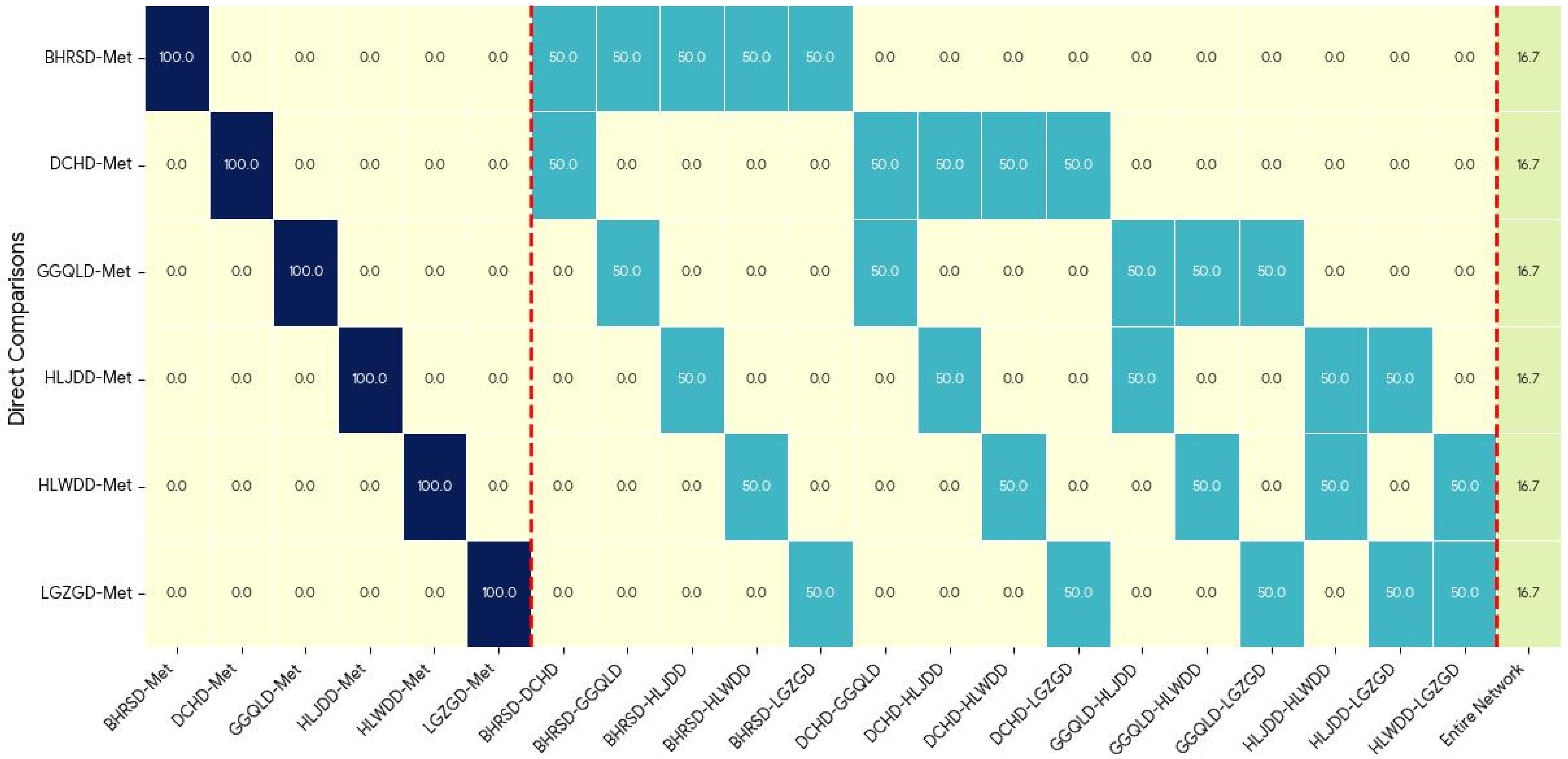

Network Contribution Matrix (FINS)

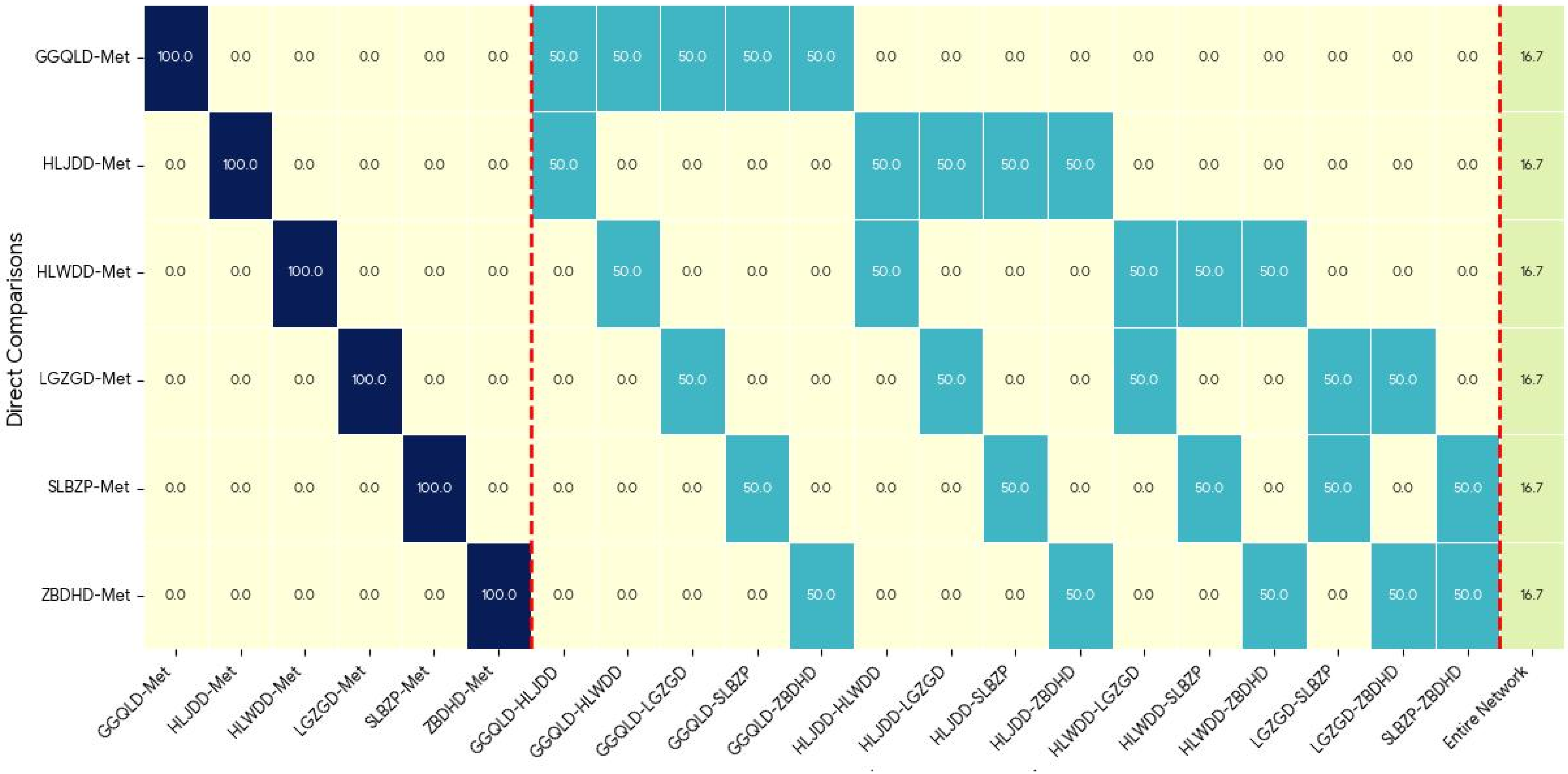

Network Contribution Matrix (HOMA-IR)

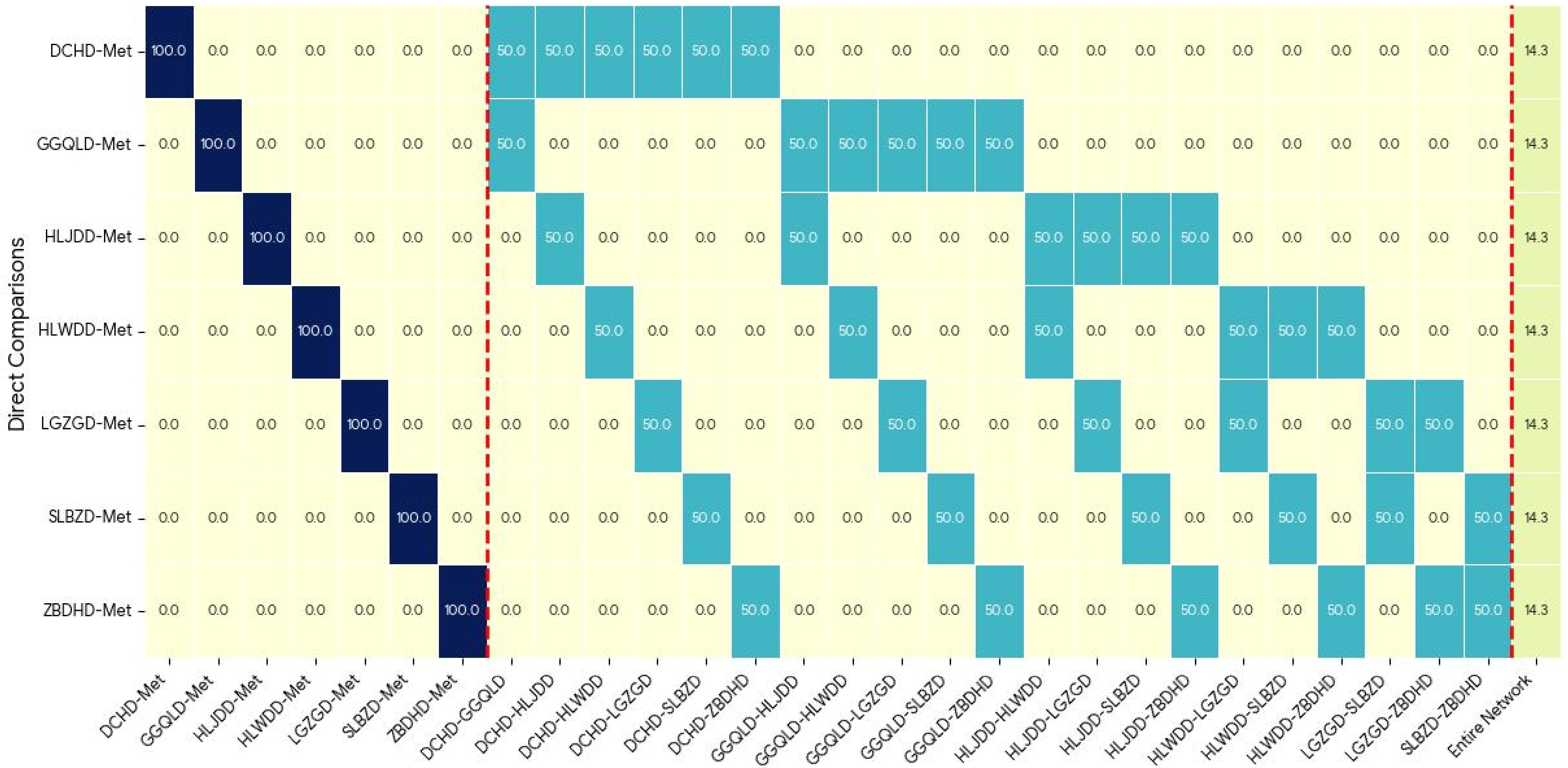

Supplement: Supplementary file 13 [file DataSheet10.pdf]
